# Rh(III)-Catalyzed Regioselective [4 + 2] Annulation of 2‑Benzyl-2H-indazole-6-carboxylic Acids with Ynamides to Access Indazole-Fused Pyrans

**DOI:** 10.1021/acs.joc.5c02242

**Published:** 2025-12-18

**Authors:** Hung-Sheng Hsieh, Kuan-Miao Liu, Chi-Min Chao, Indrajeet J. Barve, Chung-Ming Sun

**Affiliations:** † Department of Applied Chemistry, 34914 National Yang-Ming Chiao-Tung University,1001 Ta-Hsueh Road, Hsinchu 300-10, Taiwan; ‡ Department of Medicinal and Applied Chemistry, Kaohsiung Medical University, 100, Shih-Chuan first Road, Kaohsiung 807-08, Taiwan; § Department of Medical Applied Chemistry, 34899 Chung Shan Medical University, Taichung 402-01, Taiwan; ∥ Department of Medical Education, Chung Shan Medical University Hospital, Taichung 402, Taiwan; ⊥ Department of Chemistry, MES Abasaheb Garware College, Pune 411004, Maharashtra, India

## Abstract

We report a Rh (III)-catalyzed C7–H activation/[4 + 2] annulation of 2-benzyl-2*H*-indazole-6-carboxylic acids with ynamides, enabling the regioselective synthesis of indazole-fused pyrans. Mechanistic studies highlight the pivotal role of the dual directing groups (indazole and carboxylic acid) in facilitating selective C7–H bond activation, even in the presence of the competing C5–H bond. This method provides a straightforward and efficient approach for accessing structurally diverse indazole-fused pyran derivatives under mild reaction conditions. In the photoluminescence study, the synthesized compounds exhibited tunable fluorescence (410–520 nm), covering most of the visible spectrum, along with a positive solvatochromic shift in solvents of varying polarity.

## Graphical abstract

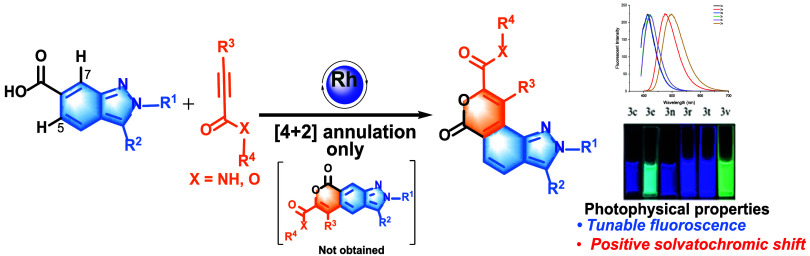

## Introduction

Indazole, a bicyclic, nitrogen-containing scaffold, is an important class of heterocycles. Although its occurrence in nature has been scarce, it has recently demonstrated significant pharmacological potential in drug discovery.^1^ For example, compound **I**, an S1P2 receptor antagonist, exhibits promising antifibrotic activity and could be used to treat fatal lung diseases.^2^ Indazole **II** acts as a sodium channel modulator, demonstrating neuroprotective effects that help reduce brain damage caused by epileptic seizures.^3^ Indazole **III** has shown antiangiogenic activity and holds potential for use in cancer treatment (Figure 1).^4^ Additionally, indazole can serve as a bioisostere for indole and benzimidazole.^5^

**Figure 1:**
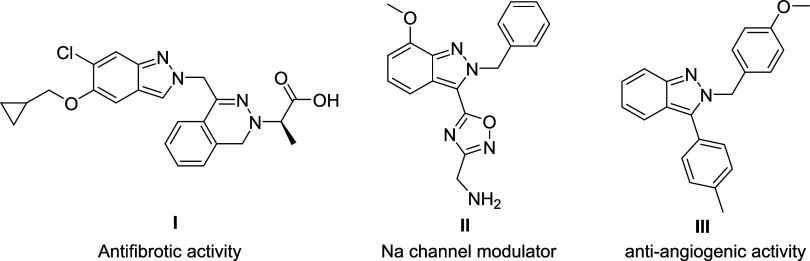
Biologically active 2H-indazoles.

The usefulness and versatility of the indazole framework have prompted synthetic chemists to pursue its functionalization, enabling access to novel derivatives with promising pharmacological properties. A literature survey revealed that most efforts to functionalize indazole have focused on the C3 position, predominantly through transition-metal-catalyzed C3–H activation or radical reactions. Significant attention has also been directed toward 2*H*-indazole group-directed C2′–H functionalization of 2-arylindazoles.^6^ Recently, transition-metal-catalyzed C2′–H/C3–H activation/cyclization cascade reactions of 2-arylindazoles with diverse substrates have emerged as a powerful strategy for constructing indazole-fused heterocycles. For instance, Kumar et al. demonstrated a Rh­(III)-catalyzed C2′–H/C3–H activation/annulation cascade between 2*H*-indazoles and alkynes to synthesize indazolo­[2,3-*a*] quinolines. Similarly, Mayakrishnan reported the synthesis of indazolo­[2,1-*a*] cinnolin-7-ium salts via Rh­(III)-catalyzed C2′–H functionalization followed by C–N bond formation using 2H-indazoles and alkynes.^7^ Expanding substrate scope, Guo developed a Rh­(III)-catalyzed reaction of 2-arylindazoles with maleimides, employing additives such as ADA or DIPEA to afford indazolo­[2,3-*a*] pyrrolo­[3,4-*c*]­quinolinones and spiroindolo­[1,2-*b*]­indazole-11,3′-pyrrolidinones.^8^ Nipate achieved Ru­(II)-catalyzed annulation of 2-aryl-2*H*-indazoles with vinylene carbonate to synthesize indazolo-fused quinolines, while Ghosh reported a related Rh­(III)-catalyzed protocol.^9^ Shao introduced a photocatalytic strategy using cobaloxime as a proton-reduction cocatalyst to enable annulation between 2-aryl-2*H*-indazoles and alkenes. Complementing this approach, Das utilized visible-light photocatalysis with piperidine-1-sulfonyl chloride as a radical precursor to facilitate the annulation of 2-arylindazoles with electron-deficient alkenes, yielding indazolo-fused quinolines.^10^ Further diversifying synthetic methodologies, Devulapally disclosed a Rh­(III)-catalyzed 1,4-addition/intramolecular annulation of 2-arylindazoles with enones to access indazolo-fused phenanthridinones.^11^ Guo also demonstrated a Rh­(III)-catalyzed C–H functionalization/cyclization cascade between 2-arylindazoles and α-diazo carbonyl compounds for the synthesis of indazoloquinolines (Scheme 1).^12^ Moreover, various strategies for the preparation of fused pyran scaffolds via C–H activation/functionalization cascade were reported.^13^

**Scheme 1:**
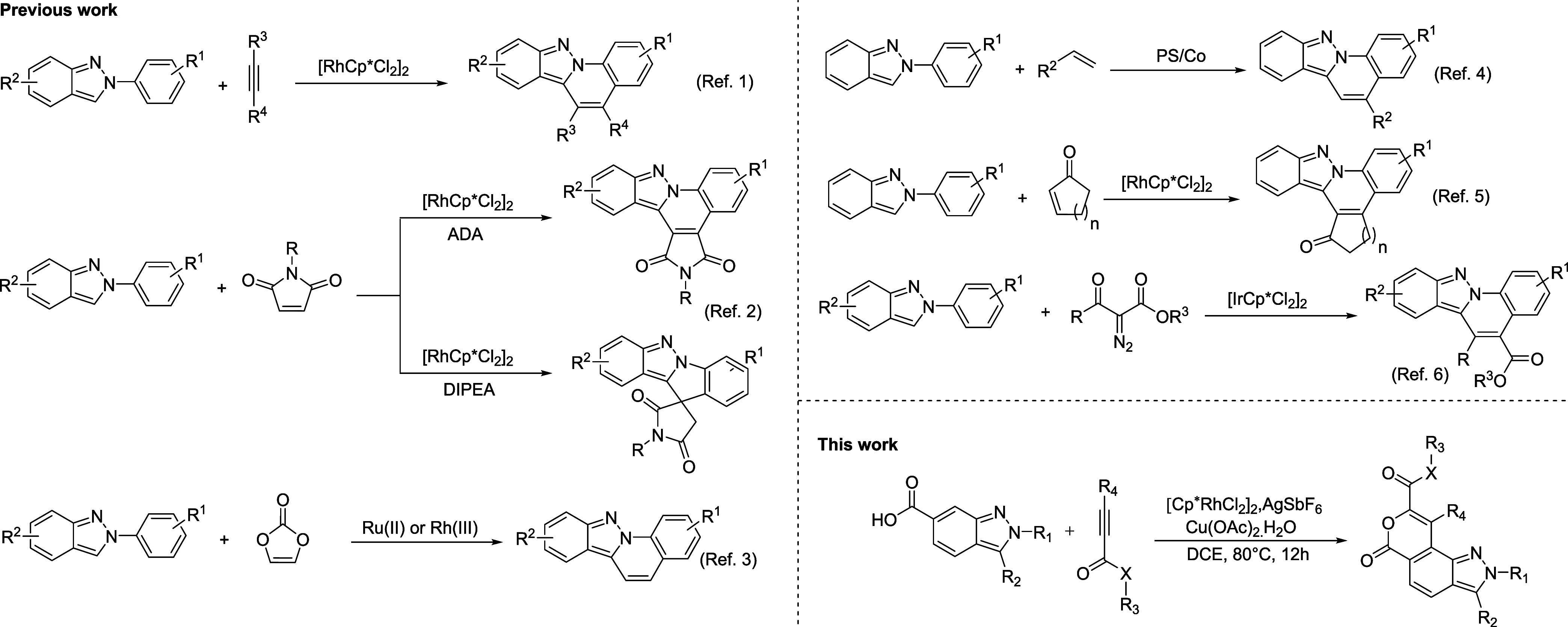
C–H Functionalization of 2H-Indazoles

The utility of 2*H*-indazole derivatives and the limited methods available for functionalizing the benzene ring of the indazole core to access an intriguing indazole-fused architecture motivated us to develop a new synthetic strategy. Herein, we report a regioselective Rh­(III)-catalyzed reaction between 2-benzyl-2*H*-indazole-6-carboxylic acids and ynamides for the synthesis of indazole-fused pyrans. This transformation proceeds through a dual directing group-assisted C7–H activation/ [4 + 2] annulation cascade, facilitated by the cooperative roles of the indazole and carboxylic acid groups.

## Results and Discussion

We began our investigation using 2-benzyl-2*H*-indazole-6-carboxylic acid **1a** and *N*-propylnon-3-ynamide **2a** as model substrates. Initially, treatment of **1a** with **2a** in the presence of catalysts such as Pd­(OAc)_2_ or PtCl_2_, using Cu­(OAc)_2_·H_2_O in 1,2-dichloroethane (DCE) at 50 °C for 6 h, did not yield the desired product, and the starting materials were recovered (Table 1, entries 1 and 2). When [Cp*IrCl_2_]_2_ was employed under the same reaction conditions, the desired product **3a** was obtained in 33% yield, accompanied by the formation of hydro-oxycarbonylated product **4a** (8%) via an intermolecular addition reaction (Table 1, entry 3). The reaction between **1a** and **2a** using [Cp*RuCl_2_]_2_ as the catalyst provided **3a** in 51% yield (Table 1, entry 4). Fortunately, the desired product **3a** was obtained in 70% yield when the catalyst was switched to [Cp*RhCl_2_]_2_ (Table 1, entry 5). The reaction was less efficient when other additives or oxidants, such as Zn­(OAc)_2_ and Ag_2_O, were employed (Table 1, entries 6 and 7). Subsequent solvent screening revealed that DCE was significantly more effective than other alternative solvents (Table 1, entries 8 and 9). Conducting the reaction at a higher temperature (80 °C) resulted in a slight yield increase compared with that of entry 5 (Table 1, entry 10). Notably, when AgSbF_6_ was employed as an additive, indazolo-fused pyran **3a** was isolated in 76% yield (Table 1, entry 11). A marked improvement in yield (90 and 93%) was observed when **1a** and **2a** were reacted in polar protic solvents (methanol and ethanol) at 50 °C (Table 1, entries 12 and 13). Treatment of **1a** with **2a** under microwave irradiation at 50 °C for 1 h to obtain **3a** in 51% (Table 1, entry 14). The desired product **3a** (60%) was formed electrochemically when (Butyl)_4_N^+^BF_4_^–^ was used as a supporting electrolyte and Pt electrode in a *t-*AmOH:H_2_O solvent mixture using a 5 mA constant current at 50 °C (Table 1, entry 15). Optimization studies identified the ideal conditions for synthesizing **3a** as follows: [Cp*RhCl_2_] _2_ (2.5 mol %) as the catalyst, Cu­(OAc)_2_·H_2_O (2.2 equiv) as the oxidant, and AgSbF_6_ (10 mol %) as an additive in ethanol at 50 °C for 6 h.

**Table 1:**
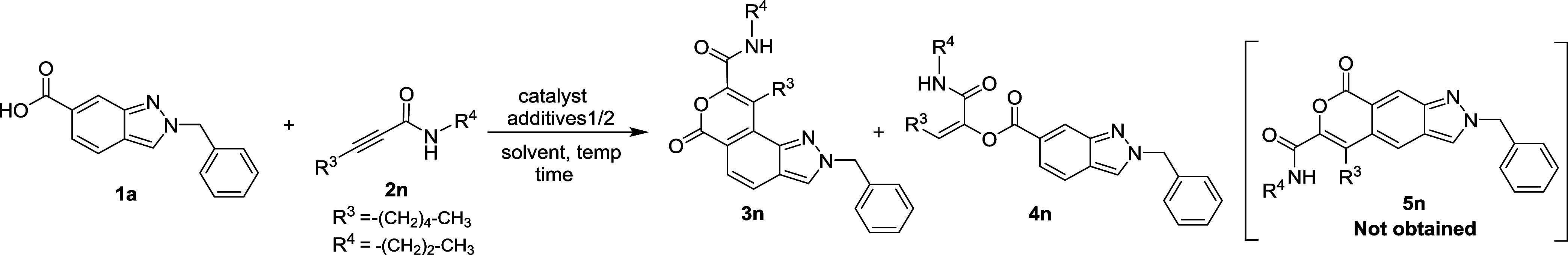
Optimization of Reaction Conditions^a^

						yield (%)^b^	
entry	catalyst	oxidant	additive	solvent	time (h)	**3n**	**4n**
1	Pd(OAc)_2_	Cu(OAc)_2_·H_2_O	-	DCE	6	0	0
2	PtCl_2_	Cu(OAc)_2_·H_2_O	-	DCE	6	0	0
3	[Cp*IrCl_2_]_2_	Cu(OAc)_2_·H_2_O	-	DCE	6	33	8
4	[Cp*RuCl_2_]_2_	Cu(OAc)_2_·H_2_O	-	DCE	6	51	0
5	[Cp*RhCl_2_]_2_	Cu(OAc)_2_·H_2_O	-	DCE	6	70	0
6	[Cp*RhCl_2_]_2_	Air	Zn(OAc)_2_	DCE	6	65	0
7	[Cp*RhCl_2_]_2_	Ag_2_O	-	DCE	6	41	3
8	[Cp*RhCl_2_]_2_	Cu(OAc)_2_·H_2_O	-	TFE	6	39	0
9	[Cp*RhCl_2_]_2_	Cu(OAc)_2_·H_2_O	-	CH_3_CN	6	45	0
10^b^	[Cp*RhCl_2_]_2_	Cu(OAc)_2_·H_2_O	-	DCE	6	73	0
11	[Cp*RhCl_2_]_2_	Cu(OAc)_2_·H_2_O	AgSbF_6_	DCE	6	76	0
12^c^	[Cp*RhCl_2_]_2_	Cu(OAc)_2_·H_2_O	AgSbF_6_	MeOH	6	90	0
13^c^	[Cp*RhCl_2_]_2_	Cu(OAc)_2_·H_2_O	AgSbF_6_	EtOH	6	**93**	0
14^d^	[Cp*RhCl_2_]_2_	Cu(OAc)_2_·H_2_O	AgSbF_6_	EtOH	1	51	0
15^e^	[Cp*RhCl_2_]_2_	CsOAc	(Butyl)_4_N^+^BF_4_^–^	*t-*AmOH:H_2_O	18	60	0

a Reaction conditions: **1a** (0.16 mmol), **2a** (0.19 mmol), catalyst (2.5 mmol %), additive 1 (2.2 equiv), additive 2 (10 mmol %), solvent (2 mL), 50 °C.

b 80 °C.

c 50 °C.

d microwave irradiation.

e Pt electrode, 5 mA, 50 °C.

After establishing the optimal reaction conditions, the substrate scope for the synthesis of indazole-fused pyrans **3** was investigated. Substrate **1**, bearing a halogen (Br), an electron-donating group (OMe), and an electron-withdrawing group (CF_3_) at the *para* position of the *N*-methyl phenyl ring (R^1^), yielded the desired products **3b**, **3c**, and **3d** in excellent yields. Notably, the methoxy-substituted substrate **1e** at the C3 position (*R* ^2^) reacted smoothly with **2a**, affording **3e** in 89% yield. The bromo-substituted **1f** was tolerated well under standard reaction conditions, providing **3f** in 66% yield. Next, the feasibility of ynamide **2** was tested. Accordingly, the reaction of *N*-methylbut-2-ynamide **2g**, *N*-isobutylbut-2-ynamide **2h**, *N*-cyclopropylbut-2-ynamide **2i**, and *N*-cyclopentylbut-2-ynamide **2j** with **1a** successfully afforded the products **3g**-**3j** in excellent yields. *N*-Benzylbut-2-ynamides bearing electron-donating (OMe), electron-withdrawing (CF_3_), and halogen (Cl) substituents at the *para* position on the benzene ring (**2k**–**2m**) also reacted efficiently with **1a**, yielding **3k**, **3l**, and **3m** in excellent yields. Concurrent changes in the *R* ^3^ and *R* ^4^ substituents using *N*-propyloct-2-ynamide **2n** yielded **3n** in 93% yield. To further expand the scope of the strategy, other alkynes were also investigated. However, when an internal alkyne, 3-phenylprop-2-yn-1-ol **2p**, was reacted with **1a**, the desired product **3p** was obtained in good yield. Unfortunately, treatment of electrophilic dimethyl acetylenedicarboxylate (DMAD) with **1a** did not deliver dicarboxylated pyran-fused indazole **3q**. Furthermore, alkyne esters were also compatible with the optimized reaction condition. Consequently, the reaction of **1a** with methyl oct-2-ynoate gave **3r** in 78% yield. Indazole **1**, possessing Br, OMe, and CF_3_ groups at the *para* position on the *N*-benzyl phenyl ring, smoothly reacted with methyl oct-2-ynoate to furnish **3s**, **3t**, and **3u** in good yields. The desired product **3v** was obtained in good yield when methoxy-substituted indazole at C3 was reacted with methyl oct-2-ynoate. The use of ethyl but-2-ynoate in the reaction provided **3w** in a 75% yield. When 2-cyclohexyl indazole carboxylic acid **1x** and 2-methylnaphthalene **1y** were reacted with **2a**, the desired products **3x** and **3y** were formed in 86% and 82% yields, respectively. Acid **1** bearing aromatic heterocycles such as the picolyl group at the *N*-benzyl group successfully participated in the reaction, affording **3z** in 79% yield. Gladly, when **1a** was reacted with an aromatic ynamide such as *N*-methyl-3-phenylpropiolamide **2aa**, the corresponding product **3aa** was obtained in 81% yield (Table 2). The representative examples 2-benzyl-9-methyl-6-oxo-N-propyl-2,6-dihydropyrano [3,4-g] indazole-8-carboxamide **3o** and ethyl 2-benzyl-9-methyl-6-oxo-2,6-dihydropyrano­[3,4-*g*]­indazole-8-carboxylate **3w** were analyzed and confirmed by spectroscopic methods. The two doublets at 7.94 and 7.77 ppm appeared in the ^1^H NMR spectrum of **3o**, whereas **3w** showed two doublets at 7.90 and 7.76 ppm and an exchangeable proton for the NH group at 7.11 ppm. The structures of **3o** and **3w** were ultimately validated by X-ray crystallography (Figure 2). In both cases, the newly formed 2*H*-pyran-2-one ring acquires a boat conformation; therefore, molecules become nonplanar.

**Figure 2:**
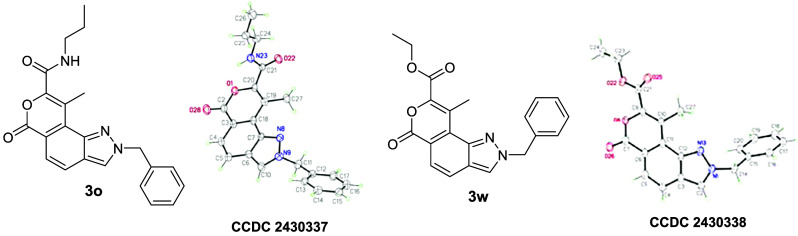
ORTEP diagram of **3o** and **3w**.

**Table 2:**
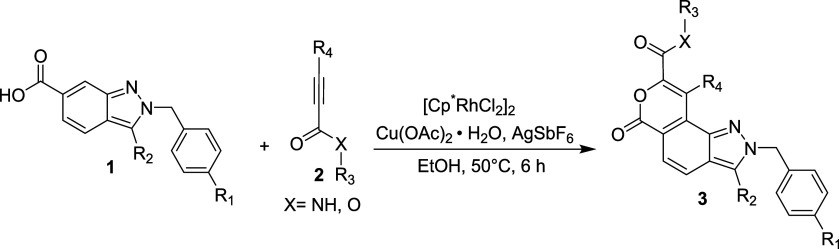

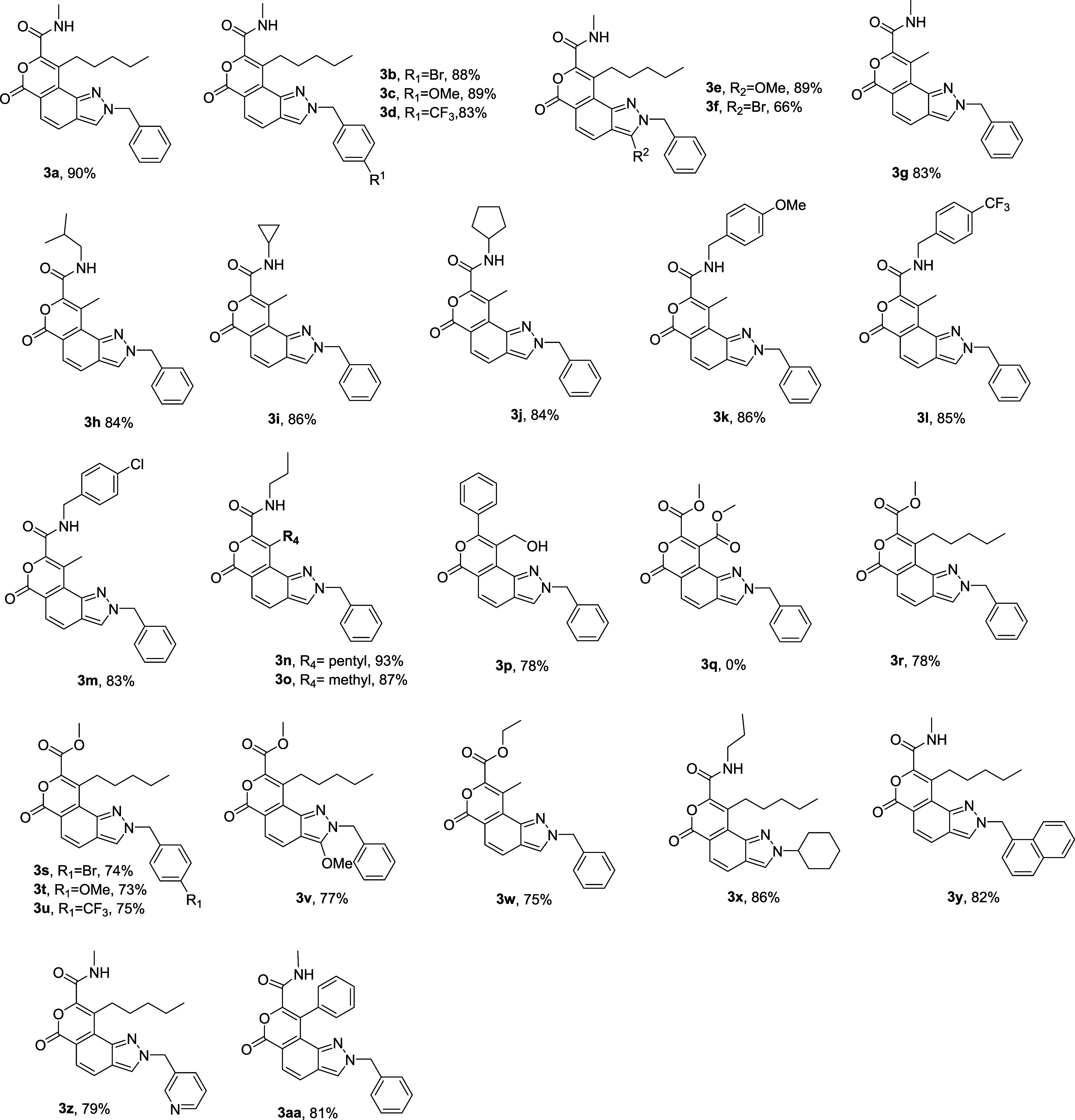
Substrate Scope of Indazolo-Fused Pyran **3** ^a^

a Reaction conditions: **1a** (0.16 mmol), **2a** (0.19 mmol), [Cp*RhCl_2_]_2_ (2.5 mmol %), CuOAc_2_.H_2_O (2.2 equiv), AgSbF_6_ (10 mmol %), EtOH (2 mL), 50 °C, 6 h.

To understand the mechanistic details of the formation of **3a**, control and deuteration experiments were conducted. When methyl 2-benzyl-2*H*-indazole-6-carboxylate **3x** was reacted with **2a** under the standard reaction conditions, the desired product was not obtained. This result indicated that the carboxylic acid group is essential as a directing group for the Rh-mediated C–H activation step. Similarly, the reaction of 2-phenyl-2*H*-indazole-6-carboxylic acid **6a** with **2a** under the optimized reaction conditions failed to yield the desired product. When the above reaction was carried out in CD_3_OD:EtOH 79%, H/D exchange was observed at the *ortho* protons of the 2-*N*-aryl moiety (Scheme 2a). This outcome indicates that the metal was chelated with lone pairs of nitrogen on the indazole and the *ortho* proton of the aryl moiety. In the deuterium exchange study, treatment of **1a** under standard reaction conditions using a deuterated cosolvent (CD_3_OD) yielded **1a′-d**_**1**_, which exhibited 19% H/D exchange at the C7–H position. This result suggests that C–H bond cleavage may be a reversible process (Scheme 2b). Additionally, the intermolecular competition reaction between **1a** and an equimolar mixture of **2a** and **2r** under optimal conditions produced **3a** and **3r** in 45 and 36% yields, respectively, indicating a preference for the electron-rich alkynamide **2a**.

**Scheme 2:**
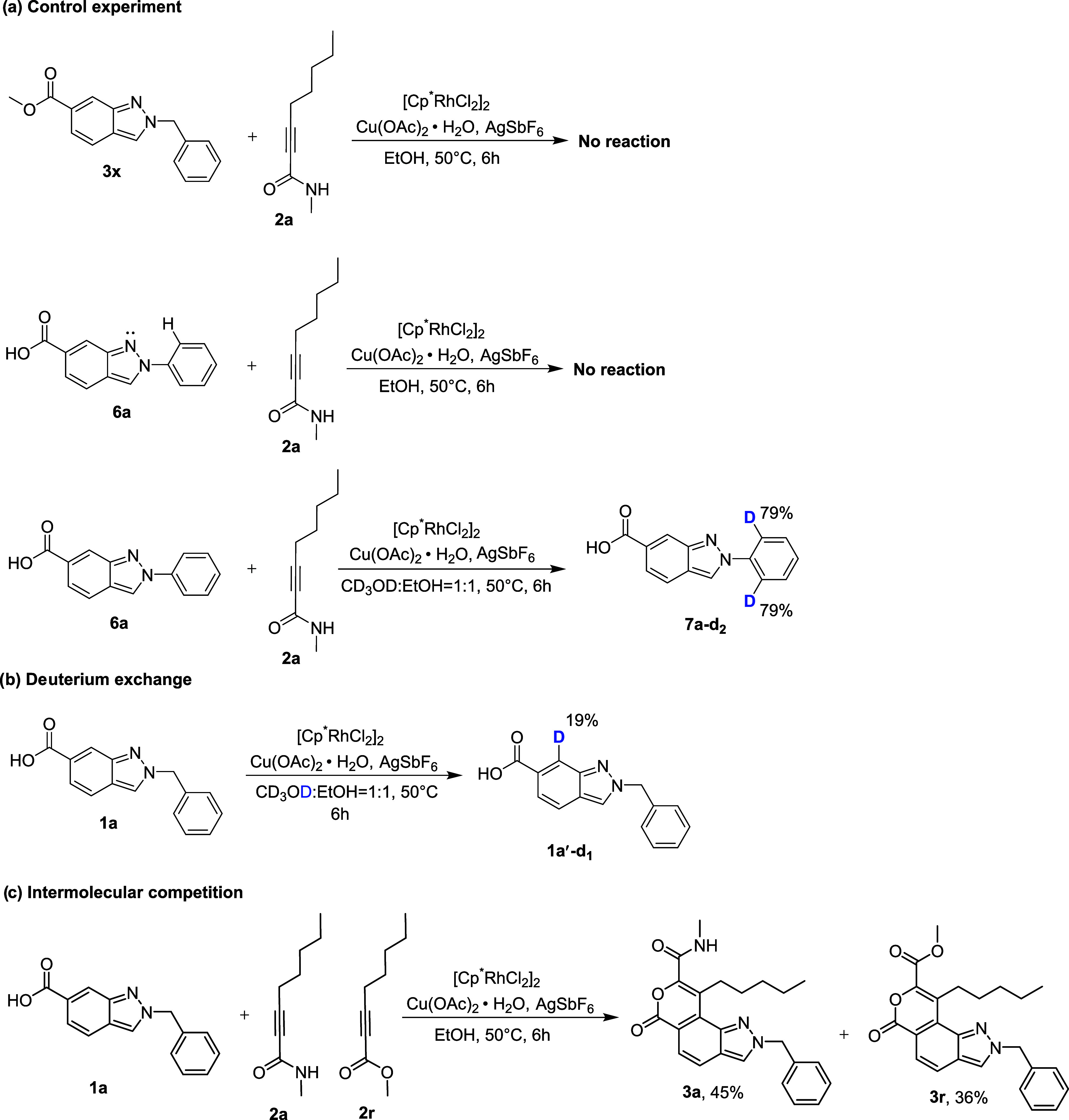
Mechanistic Study

Based on control experiments and experimental observations, a plausible mechanism for the formation of **3a** is outlined in Scheme 3. The reaction begins with the dimeric [Cp*RhCl_2_]_2_ catalyst reacting with Cu (OAc)_2_ or AgSbF_6_, generating active monomeric Cp*Rh­(III)­L_2_ species **I**. Subsequently, the indazole and carboxylic acid double-directing group assists in regioselective C7–H bond activation, yielding five-membered rhodacycle intermediate **B**. Coordination and precomplexation of the Rh metal with the carbonyl of **2a** form intermediate **C**.^14^ Next, insertion of the electronically polarized triple bond of ynamide **2a** into the Rh–C bond at the slightly positively charged β position then produces intermediate **D**. Finally, intermediate **D** undergoes reductive elimination to furnish **3a** and a [Cp*Rh­(I)] species. The [Cp*Rh­(I)] species formed is then oxidized by Cu­(OAc)_2_ (Cu­(II)) to regenerate the active [Cp*Rh­(III)­(OAc)_2_] catalyst with the concomitant reduction of Cu­(II) to Cu­(I). The molecular oxygen (O_2_) in the air reoxidizes Cu­(I) species to Cu­(I) species.^15^

**Scheme 3:**
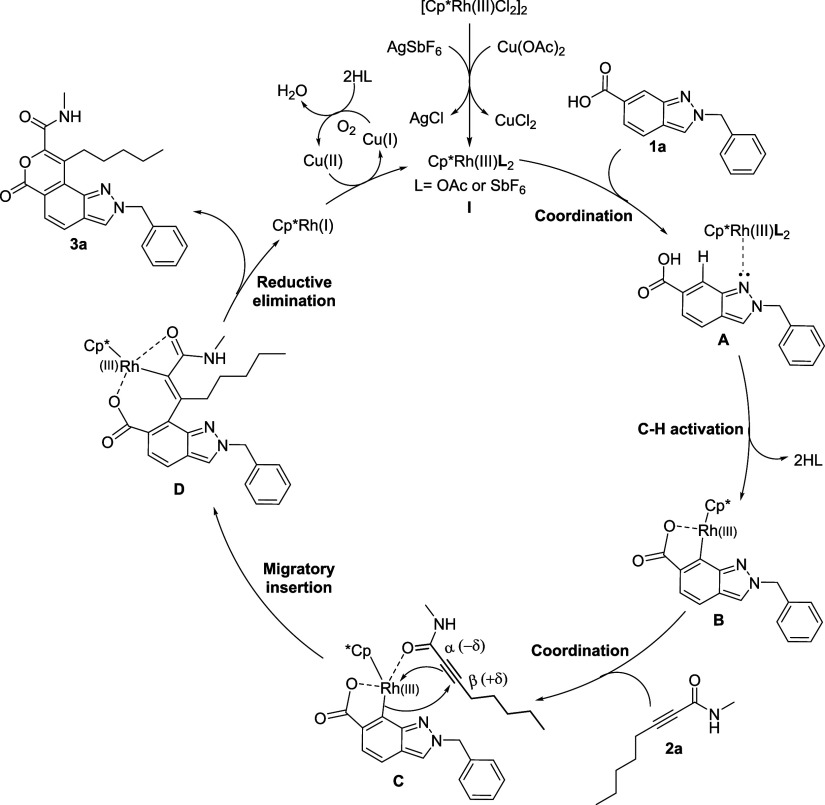
Plausible Mechanism for the Formation of **3a**

Indazole derivatives are widely used for the development of fluorescent materials, probes, and dyes due to their fluorescence properties.^16^ Owing to the fluorescent nature of most of the synthesized indazole-fused pyrans **3**, absorption and fluorescence measurements for the compounds **3c**, **3e**, **3n**, **3r**, **3t**, and **3v** were carried out by ultraviolet (UV) absorption and photoluminescence (PL) spectroscopy (for details, see the SI, Figures S1–S6 and Table S1). The fluorescence emission ranged from 410 nm (blue) to 520 nm (green), depending on the different substituents introduced into the indazole-fused pyrans, and **3** was observed. This tunability highlights the strong influence of the molecular structure on the photophysical behavior (Figure 3). Among them, compound **3v** was selected for further study due to its most red-shifted emission and stability in solution. Solubility tests indicated that this green-emitting compound is soluble in organic solvents such as dichloromethane, tetrahydrofuran, and toluene, but insoluble in water, reflecting its hydrophobic character. To assess its environmental sensitivity, the emission behavior was examined in solvents of varying polarity. A distinct positive solvatochromic shift was observed: the emission maximum red-shifted from 410 nm in toluene to approximately 520 nm in dimethyl sulfoxide (DMSO) (Figure 4). This behavior suggests that the compound undergoes significant intramolecular charge transfer (ICT) in the excited state with increased stabilization in more polar solvents. The compound also demonstrated strong and consistent fluorescence across different solvent environments, supporting its suitability for applications in polarity-sensitive fluorescence sensing and bioimaging. Its stable emission and structural tunability offer great potential for future development as an environmental or biological probe.

**Figure 3:**
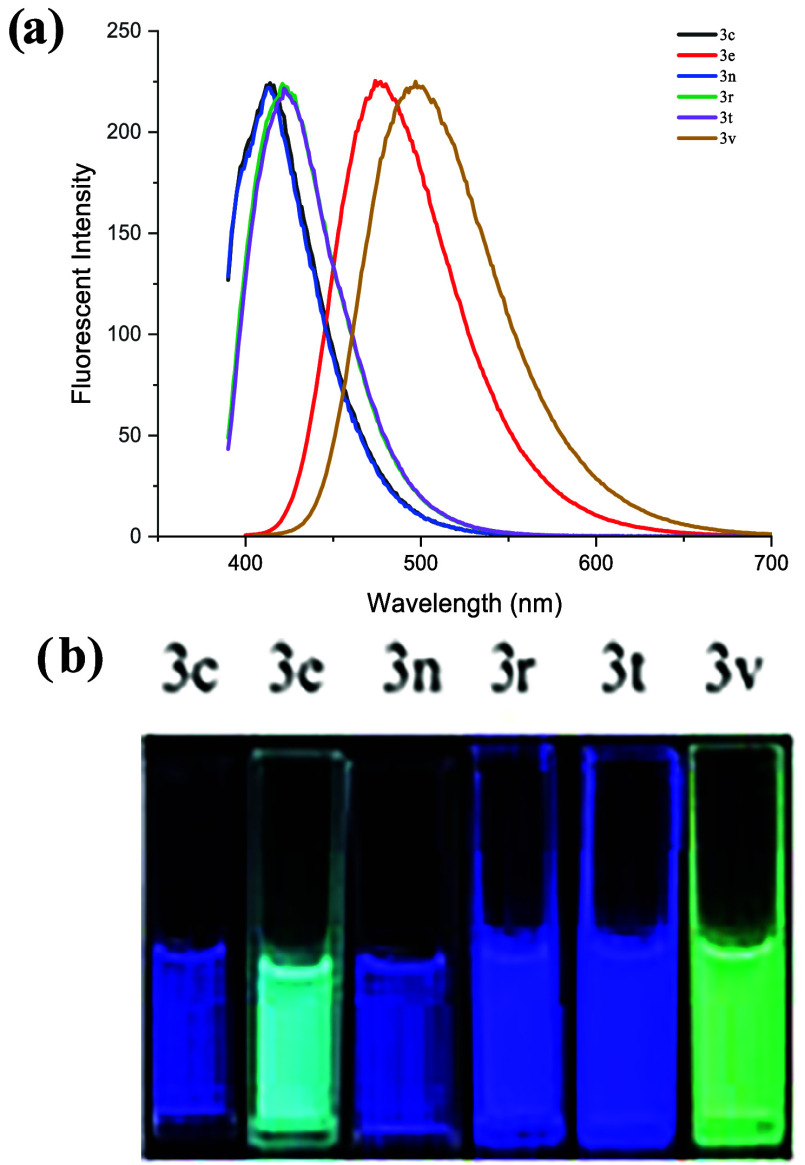
Spectral properties of indazole-fused pyrans **3** in acetone (10^–5^ M). (a) Fluorescence emission spectra. (b) Photograph of **3c**, **3e**, **3n**, **3r**, **3t**, and **3v** in acetone solution under UV lamp (365 nm).

**Figure 4:**
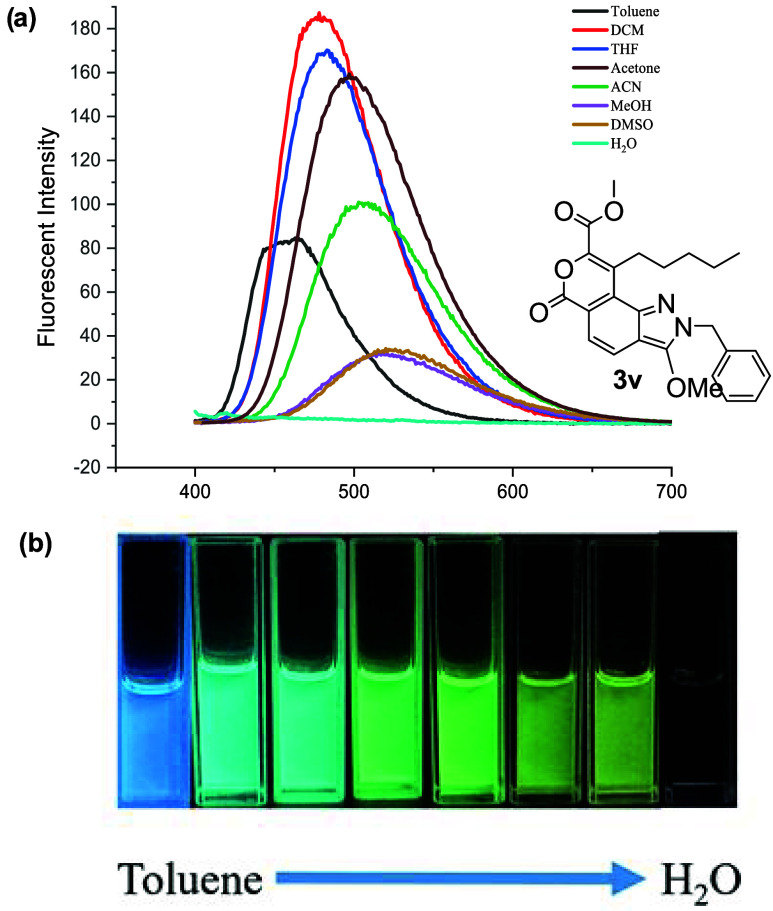
Spectral properties of compound **3v** in various solvents (10^–5^ M). (a) Fluorescence emission spectra. (b) Photograph of **3v** in various solvents under a UV lamp (365 nm).

Molecular hybridization is an important strategy to design multitarget drug candidates via a combination of two or more pharmacophoric moieties from different bioactive compounds into a single entity called a hybrid molecule.^17^ Tryptamine is a privileged structure found in naturally occurring alkaloids, various commercial drugs, and the human brain. Due to its similarity to the neurotransmitter serotonin, it can interact with serotonin receptors (5-HT). Apart from this, it can also interact with dopamine and α-adrenergic receptors. Researchers are developing tryptamine-based hybrid scaffolds to treat Alzheimer’s disease.^18^ In accordance with it, the potential synthetic application of the synthesized product was accomplished via derivatizing **3w** with tryptamine to yield an indazole-fused pyran-linked tryptamine hybrid **9w**. Initially, **3w** was hydrolyzed using conc. HCl to obtain acid **8w**. The acid **8w** was then coupled with tryptamine in the presence of EDCI and HOBT to furnish the indazole-fused pyran-linked tryptamine hybrid **9w** (Scheme 4).

**Scheme 4:**
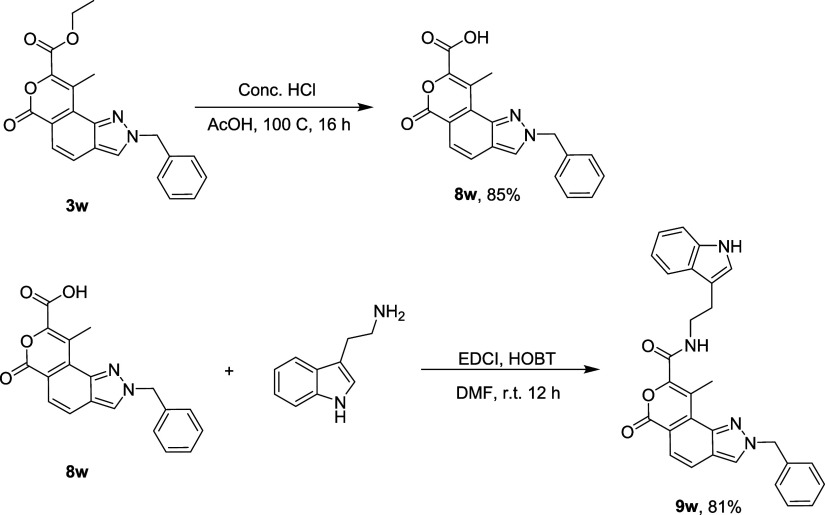
Postsynthetic Modification of **3w**

## Conclusion

In summary, we have established a Rh (III)-catalyzed protocol for the synthesis of indazole-fused pyrans through a regioselective C7–H activation/[4 + 2] annulation strategy. The cooperative action of the indazole and carboxylic acid as directing groups ensures precise C7–H bond activation only, circumventing the challenge posed by the adjacent C5–H bond. The method’s versatility is further demonstrated by its compatibility with ynoates, yielding the desired products in good yields. This approach not only expands the toolbox for indazole functionalization but also opens new avenues for the development of indazole-fused heterocycles with potential applications in drug discovery and materials science. Photoluminescence study of the representative compounds showed a bathochromic shift in fluorescence emissions. Among them, the green-emitting compound at 520 nm was chosen for further exploration due to its strong and stable fluorescence. Its pronounced solvatochromic behavior and photostability may prove to be promising candidates for applications in fluorescence sensing and advanced material design.

## Supplementary Material



## Data Availability

The data underlying this study are available in the published article and its Supporting Information.

## Notes

Experimental procedures; characterization data and spectral data; and X-ray data for compounds **3o** and **3w** (PDF)

## Notes

This article is dedicated to Professor Iwao Ojima on his 80th birthday.
