# Supplementary material for: Rh(III)-Catalyzed Regioselective [4 + 2] Annulation of 2‑Benzyl-2H-indazole-6-carboxylic Acids with Ynamides to Access Indazole-Fused Pyrans
Source: J Org Chem. 2025 Dec 18;91(1):200–8. doi: 10.1021/acs.joc.5c02242 (PMC12797292; doi:10.1021/acs.joc.5c02242)
Supplement: Supplementary file 1 [file jo5c02242_si_001.pdf]

Supplementary Information

Rh(III)-Catalyzed Regioselective [4+2] Annulation of 2-Benzyl-2H-indazole-6-carboxylic Acids with Ynamides to Access Indazole-Fused Pyrans

Hung-Sheng Hsieh,[1,3] Kuan-Miao Liu,[3,4] Chi-Min Chao,[3,4] Indrajeet J. Barve,[5] and Chung-Ming Sun[1,2]*

[1] *Department of Applied Chemistry, National Yang-Ming Chiao-Tung University,1001 Ta-Hsueh Road, Hsinchu 300-10, Taiwan*

[2] *Department of Medicinal and Applied Chemistry, Kaohsiung Medical University, 100, Shih-Chuan 1st Road, Kaohsiung 807-08, Taiwan*

[3] *Department of Medical Applied Chemistry, Chung Shan Medical University, Taichung 402-01, Taiwan*

[4] *Department of Medical Education, Chung Shan Medical University Hospital, Taichung, 402, Taiwan*

[5] *Department of Chemistry, MES Abasaheb Garware College, Pune 411004, Maharashtra, India*

E-mail: cmsun@nycu.edu.tw

Table of Contents

**General Information**	**S2**
**Analytical Methods**	**S2**
**General procedure for the synthesis of 2**	**S2**
**A representative procedure for the synthesis of 3a**	**S2-S3**
**Mechanistic Study**	**S3-S6**
**Spectral properties**	**S6-S8**
**Post-synthetic modification of 3w**	**S9-S13**
**Characterization Data of 3a-3z, 3aa, 4n and 3a'**	**S14-S25**
**Spectral Data of 3a-3z, 3aa, 4n and 3a'**	**S26-S67**
**X-Ray Crystallographic Data of 3o**	**S68-S79**
**X-Ray Crystallographic Data of 3w**	**S79-S85**

General Information

All reactions were carried out in oven-dried round bottoms. All other chemicals were also purchased from Sigma-Aldrich and TCI and were used as received. All the solvents were distilled before use.

Analytical Methods

Analytical thin-layer chromatography (TLC) was performed using 0.25mm silica gel coated plates. Flash chromatography was performed using the indicated solvent and silica gel 60 (230-400 mesh). ^{1}H NMR and ^{13}C NMR spectra were recorded on 400 MHz spectrometers. Chemical shifts are reported in parts per million (ppm) on the δ scale from an internal standard (TMS). High resolution mass spectra (HRMS) were recorded in ESI mode using TOF mass spectrometer. Single crystal X-ray diffraction data was collected in Rigaku XtaLAB Synergy DW diffractometer. The benzoic acids were purchased from commercial sources.

General procedure for the synthesis of 2

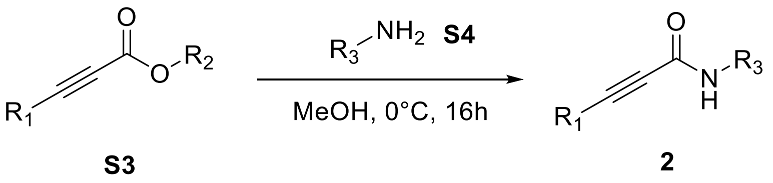

To an oven-dried 50 mL round-bottom flask equipped with magnetic stir bar, a solution of alkyl ester **S3** (1equiv.) in 4 mL MeOH, alkyl amine **S4** (1.2 equiv.) was added dropwise at 0 °C for 16 h. After removal of the solvent under reduced pressure, purification was performed by flash column chromatography on silica gel with hexane/ethyl acetate (3:1) as eluent to afford corresponding products.

A representative procedure for the synthesis of 3a

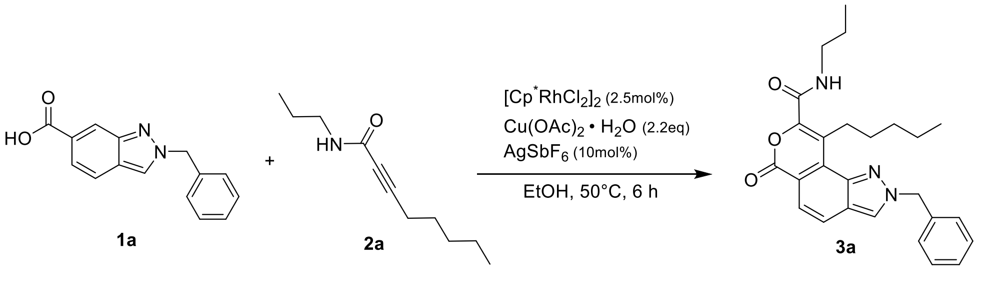

To an oven-dried seal tube equipped with magnetic stir bar, **1a** (50 mg, 0.2 mmol), **2a**

(36.6 mg, 0.24 mmol), [Cp*RhCl$_2$]$_2$ (3 mg, 2.5 mol%) and Cu(OAc)$_2$·H$_2$O (86.9 mg, 0.44 mmol) and AgSbF$_6$ (6.8 mg, 10 mol%) were dissolved in EtOH (2 mL). The resulting solution was stirred at 50°C (oil bath) for 6 h. After removal of the reaction solvent under reduced pressure, purification was performed by flash column chromatography on silica gel using hexane/ethyl acetate (3:1) as an eluent to afford the desired product **3a**.

Gram-scale synthesis of 3v

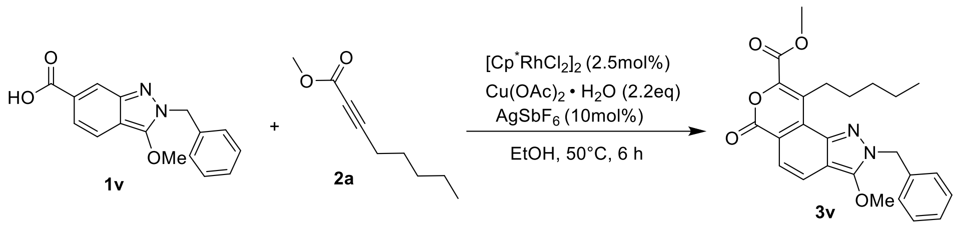

To an oven-dried seal tube equipped with a magnetic stir bar, **1a** (1 g, 3.54 mmol), **2a** (0.65 g, 4.25 mmol), [Cp*RhCl$_2$]$_2$ (54 mg, 2.5 mol%) and Cu(OAc)$_2$·H$_2$O (1.5 g, 7.78 mmol) and AgSbF$_6$ (0.12 g, 10 mol%) were dissolved in EtOH (30 mL). The resulting solution was stirred at 50°C (oil bath) for 6 h. After completion of the reaction, the solvent was removed under reduced pressure. The crude product was purified by flash column chromatography on silica gel using hexane/ethyl acetate (3:1) as an eluent to afford 3v in 74% yield (1.1 g).

Preparation of a single crystal of 3o and 3w

In a 5 mL sample vial, 5 mg of **3o** or **3w** was dissolved in ethyl acetate (2.5 mL), and 1 mL of *n*-hexane was added to it. The vial was kept at room temperature for 5 days. The slow evaporation of the solvent yielded crystals suitable for single-crystal X-ray diffraction.

Mechanistic Study

a) Deuterium exchange

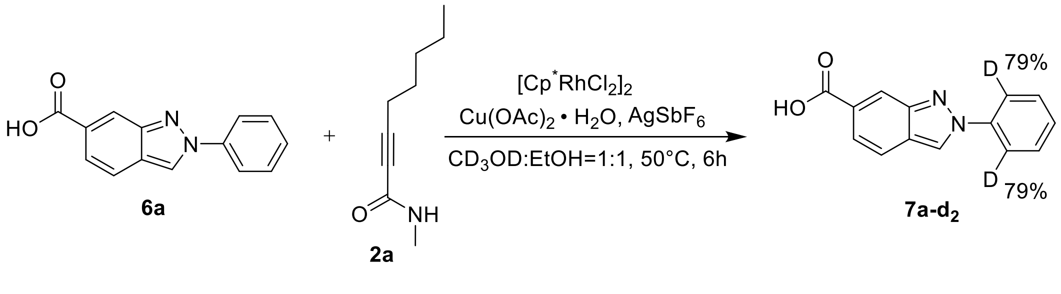

To an oven-dried seal tube equipped with magnetic stir bar, **6a** (50 mg, 0.2 mmol), **2a** (35 mg, 0.23 mmol) $[Cp^*RhCl_2]_2$ (3 mg, 2.5 mol%) and $Cu(OAc)_2 \cdot H_2O$ (86.9 mg, 0.44 mmol) and $AgSbF_6$ (6.8 mg, 10 mol%) were dissolved in EtOH (1mL) and CD_3OD (1mL). The resulting solution was stirred at 50°C (oil bath) for 6 h. After removal of the solvent under reduced pressure, purification was performed by flash column chromatography on silica gel with hexane/ethyl acetate (3:1) as eluent to afford **7a-d₂**. The H/D exchange was found to be 79 % at the ortho protons of the 2-*N*-aryl moiety.

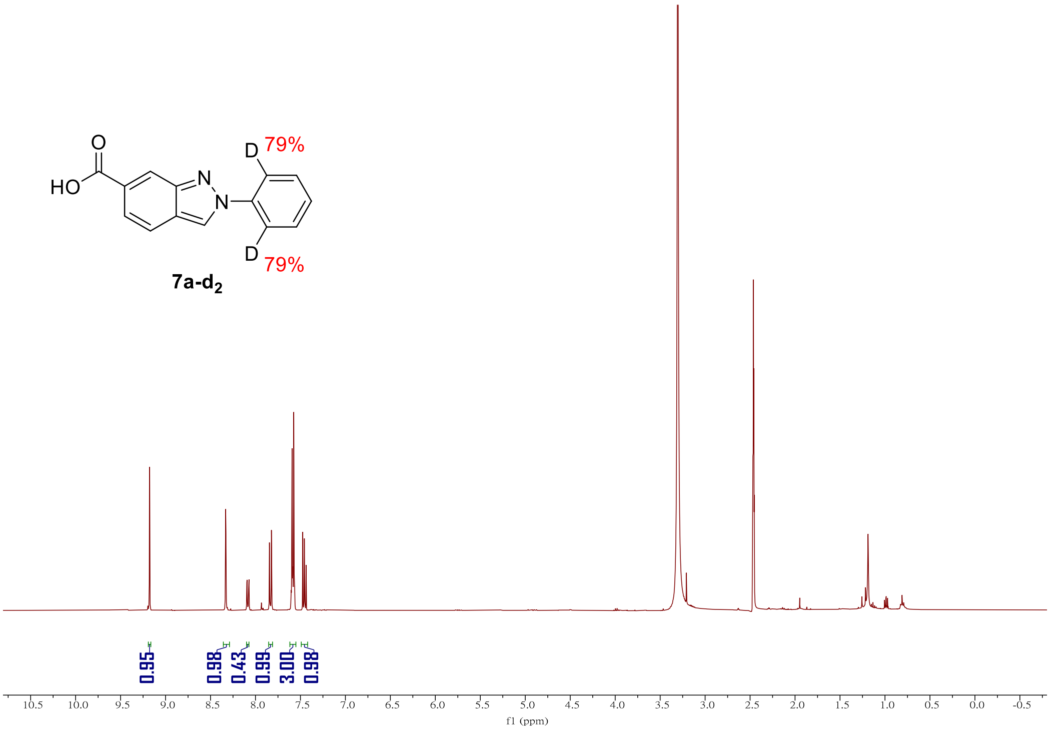

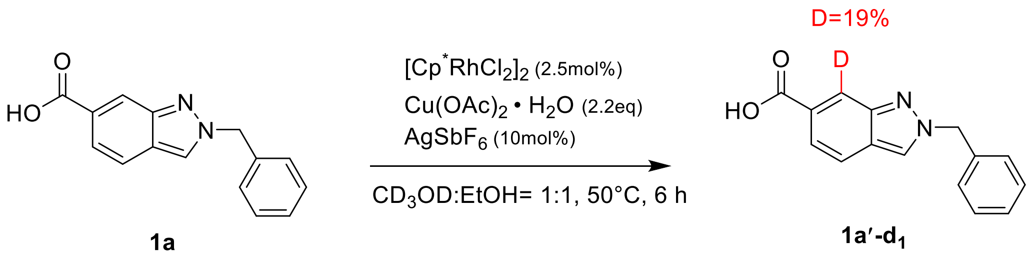

To an oven-dried seal tube equipped with magnetic stir bar, **1a** (50 mg, 0.2 mmol), $[Cp^*RhCl_2]_2$ (3 mg, 2.5 mol%) and $Cu(OAc)_2 \cdot H_2O$ (86.9 mg, 0.44 mmol) and $AgSbF_6$ (6.8 mg, 10 mol%) were dissolved in EtOH (1mL) and CD_3OD (1mL). The resulting solution was stirred at 50°C (oil bath) for 6 h. After removal of the solvent under reduced pressure, purification was performed by flash column chromatography on silica gel with hexane/ethyl acetate (3:1) as eluent to afford corresponding product. The H/D exchange was found to be 19 % at the protons attached to C-7 in the recovered **1a'-d₁**.

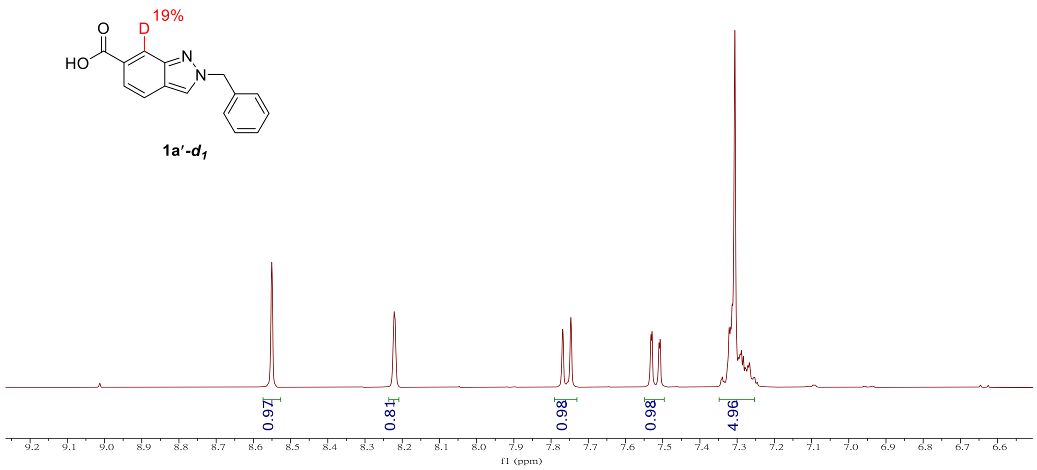

b) Intermolecular competition experiment

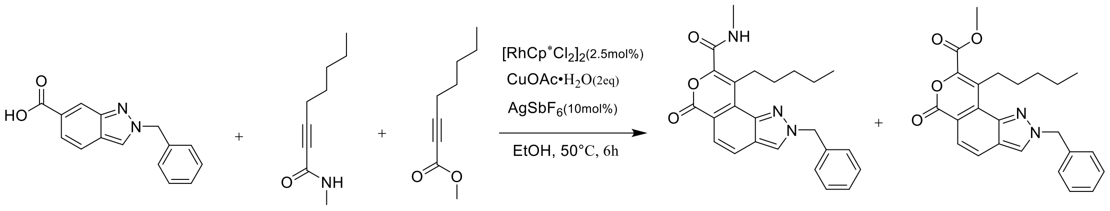

To an oven-dried seal tube equipped with magnetic stir bar, **1a** (50 mg, 0.2 mmol), **2n** (30.3 mg, 0.2 mmol), **2r** (30.4 mg, 0.2 mmol), $[Cp^*RhCl_2]_2$ (3 mg, 2.5 mol%) and $Cu(OAc)_2 \cdot H_2O$ (86.9 mg, 0.44 mmol) and $AgSbF_6$ (6.8 mg, 10 mol%)were dissolved in EtOH (2 mL). The resulting solution was stirred at 50 °C (oil bath) for 6 h. After removal of the solvent under reduced pressure, purification was performed by flash column chromatography on silica gel with hexane/ethyl acetate (3:1) as eluent to afford the **3a** and **3r** in 45% and 36% yields.

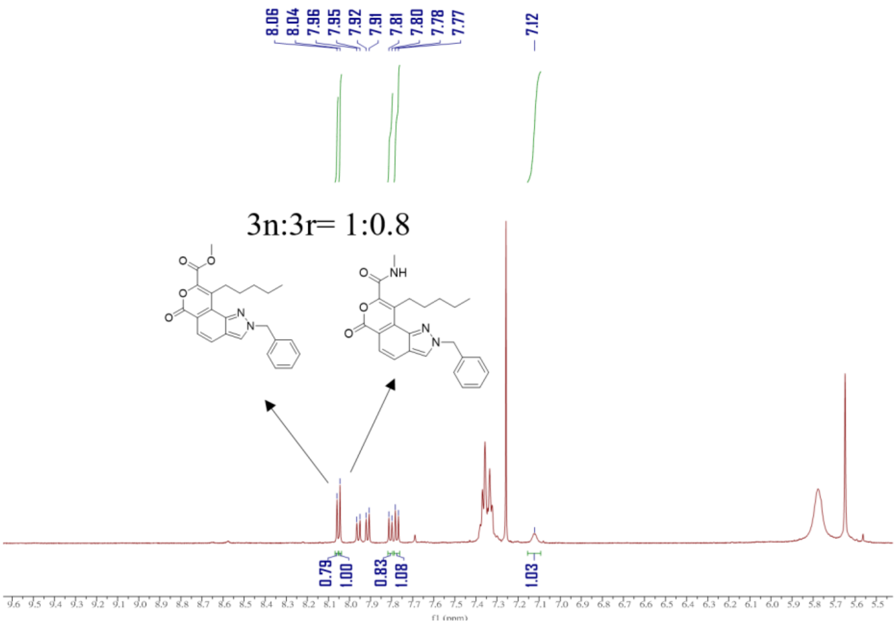

Photophysical properties

UV-visible absorption and fluorescence emission spectra

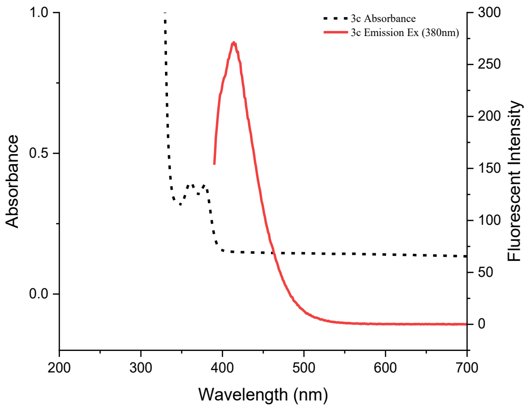

Figure S1. UV-visible absorption and fluorescence emission spectra of **3c** in acetone at RT (10^{-5}M).

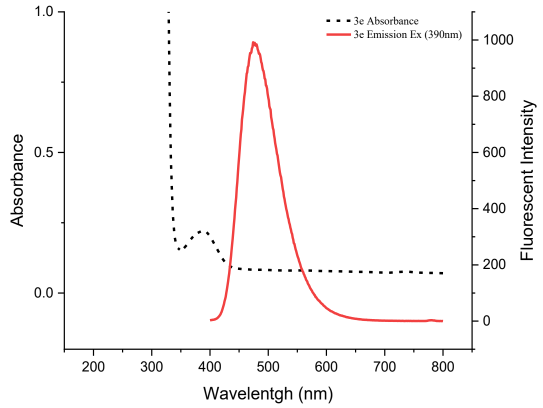

Figure S2. UV-visible absorption and fluorescence emission spectra of **3e** in acetone at RT (10^{-5}M).

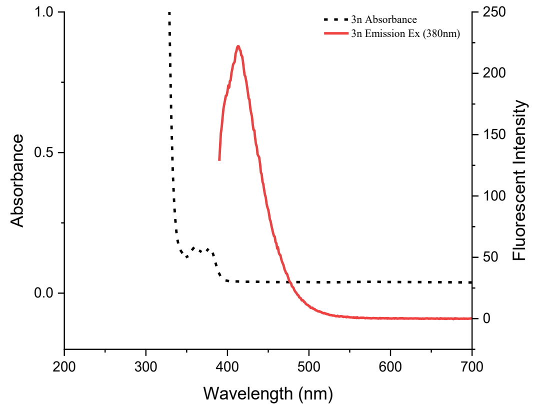

Figure S3. UV-visible absorption and fluorescence emission spectra of **3n** in acetone at RT (10^{-5}M).

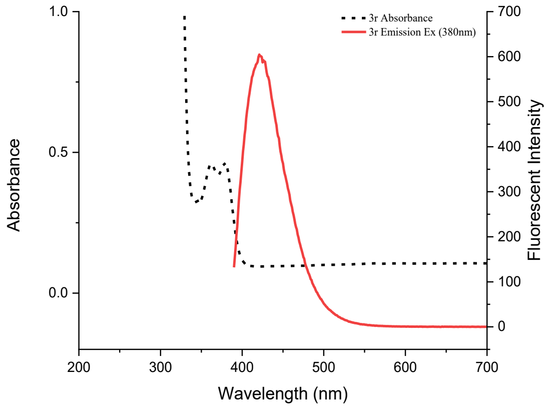

Figure S4. UV-visible absorption and fluorescence emission spectra of **3r** in acetone at RT (10^{-5}M).

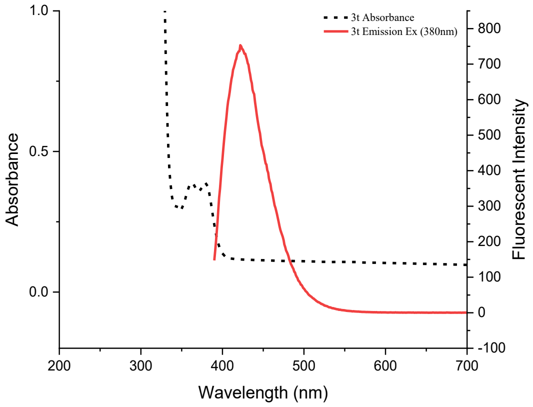

Figure S5. UV-visible absorption and fluorescence emission spectra of **3t** in acetone at RT (10^{-5}M).

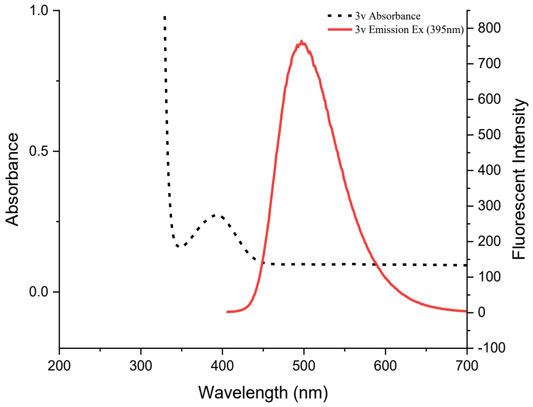

Figure S6. UV-visible absorption and fluorescence emission spectra of **3v** in acetone at RT (10^{-5}M).

Table S1. Spectral Properties of selected compound in acetone at RT (10^{-5}M)

Compound	λ_{abs}^{max} [nm]	λ_{em}^{max} [nm]	Stokes shift [nm]
**3c**	377	414	37
**3e**	389	474	85
**3n**	361	413	52
**3r**	379	421	42
**3t**	380	422	42
**3v**	393	497	104

Post-synthetic modification of 3w

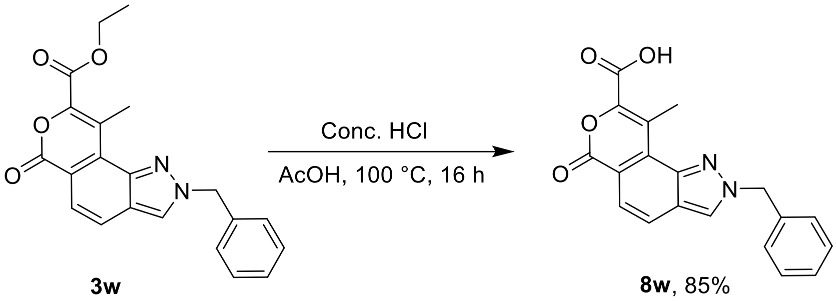

3w **8w**, 85%

To an oven-dried 50 mL round-bottom flask equipped with a magnetic stir bar, was added ester **3w** (100 mg, 0.27 mmol) and AcOH (5 mL). To the above solution, Conc. HCl (0.8 mL) was added, and the reaction was heated at 100 °C (oil bath) for 16 h. After completion of the reaction, the solvent was removed under reduced pressure. The residue was washed with water (20 mL x 2) to obtain crude product **8w** (78.4 mg, 85%).

2-Benzyl-9-methyl-6-oxo-2,6-dihydropyrano[3,4-g]indazole-8-carboxylic acid (8w)

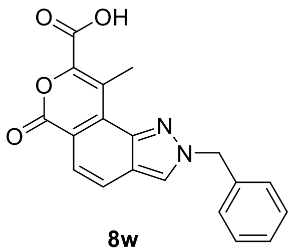

8w

Flash chromatography for purification: hexane/ethyl acetate = 1:2. White solid; Yield = 65 mg (85%); ^{1}H NMR (600 MHz, DMSO-d_6) δ 8.73 (s, 1H), 8.00 (d, J = 8.8 Hz, 1H), 7.73 (d, J = 8.7 Hz, 1H), 7.39 – 7.28 (m, 5H), 5.77 (s, 2H), 3.03 (s, 3H). ^{13}C NMR (151 MHz, DMSO-d_6) δ 162.8, 160.7, 144.9, 141.5, 136.7, 130.8, 129.1, 128.5, 128.4, 126.0, 125.6, 124.3, 120.2, 120.0, 119.7, 57.3, 16.0. HRMS (ESI) m/z: [M+H]$^+$ Calcd for $C_{19}H_{15}N_2O_4$ 335.1026, found 335.1027.

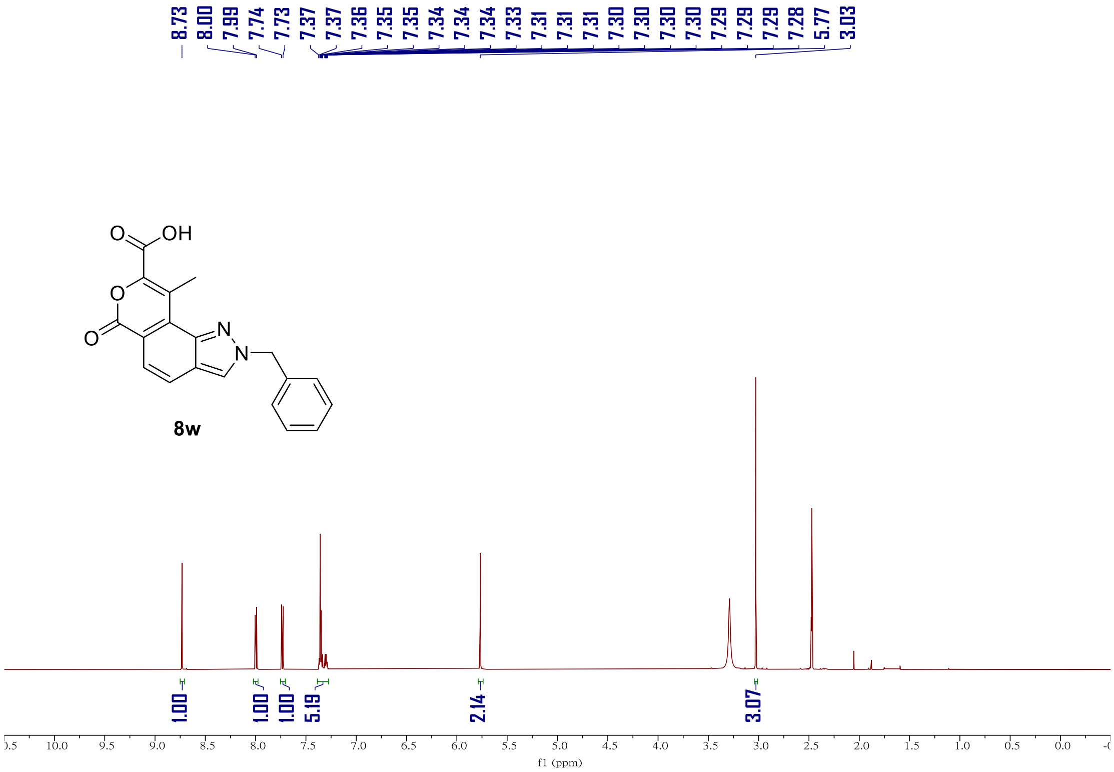

^{1}H spectrum (600 MHz) of compound **8w** in DMSO-d_6

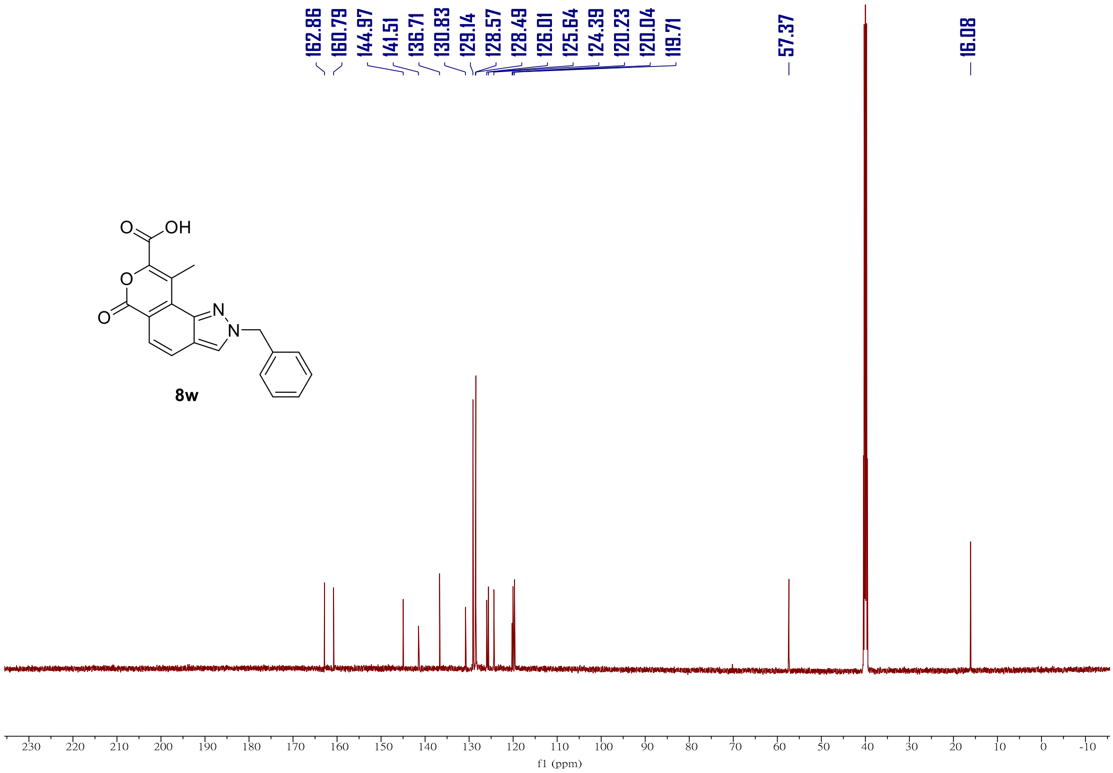

^{13}C{^{1}H} spectrum (151 MHz) of compound **8w** in DMSO- d_6

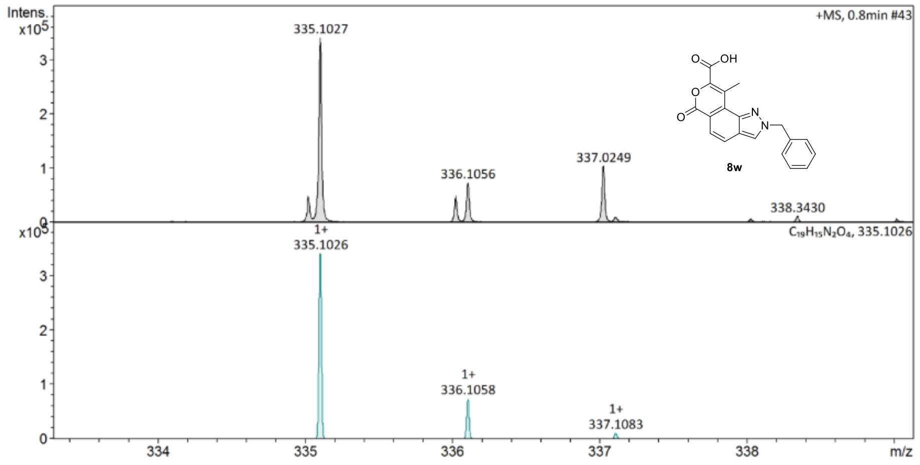

Display Report

Meas. m/z	#	Ion Formula	m/z	err [ppm]	mSigma	# Sigma	Score	rdb	e⁻ Conf	N-Rule	Adduct
335.1027	1	C19H15N2O4	335.1026	-0.1	0.4	1	100.00	13.5	even	ok	M+H

HRMS Mass (ESI) spectrum of compound **8w**

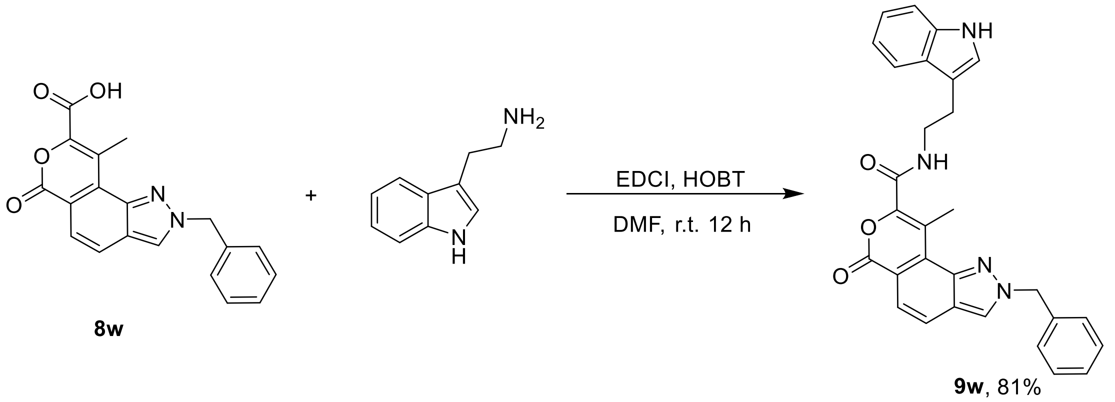

To an oven-dried 50 mL round-bottom flask equipped with a magnetic stir bar, was added **8w** (70 mg, 0.2 mmol) and DMF (4 mL). To the above solution was added EDCI (49 mg, 0.3 mmol), HOBT (23 mg, 1.6 mmol), and tryptamine (41 mg, 0.24 mmol) and the reaction was stirred at room temperature for 12 h. After completion of the reaction, the solvent was removed under reduced pressure. The residue was diluted with water (10 mL) and extracted with ethyl acetate (20 mL x 2). The crude product was purified by flash column chromatography on silica gel using ethyl acetate/hexane (1:1) as an eluent to afford **9w** (80 mg, 81%).

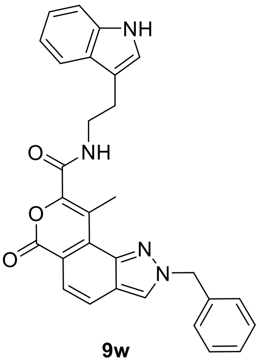

9w

^{1}H NMR (600 MHz, $CDCl_3$) δ 8.11 (s, 1H), 7.97 (s, 1H), 7.88 (d, J = 8.7 Hz, 1H), 7.74 (d, J = 8.7 Hz, 1H), 7.66 (d, J = 7.9 Hz, 1H), 7.37 (dq, J = 14.8, 7.8, 7.3 Hz, 6H), 7.21 (dt, J = 15.5, 6.7 Hz, 2H), 7.13 (d, J = 12.3 Hz, 2H), 5.66 (s, 2H), 3.75 (q, J = 6.8 Hz, 2H), 3.30 (s, 3H), 3.10 (t, J = 7.1 Hz, 2H). $^{13}C\{^1H\}$ NMR (151 MHz, $CDCl_3$) δ 161.1, 160.9, 145.3, 142.0, 136.4, 135.0, 132.4, 129.0, 128.7, 128.3, 127.2, 125.8, 123.1, 122.5, 122.1, 122.1, 120.4, 120.3, 119.8, 119.4, 118.7, 112.8, 111.2, 58.2, 39.8, 25.3, 15.4. HRMS (ESI) m/z: $[M+H]^+$ Calcd for $C_{29}H_{25}N_4O_3$ 477.1921, found 477.1921.

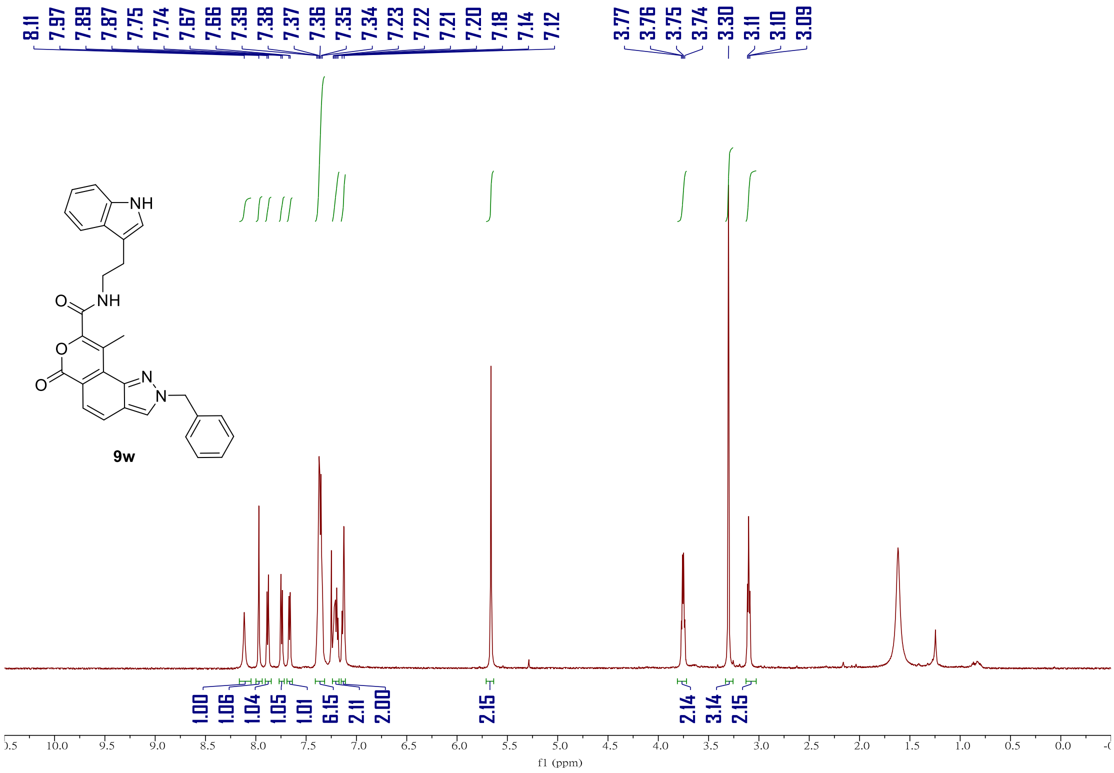

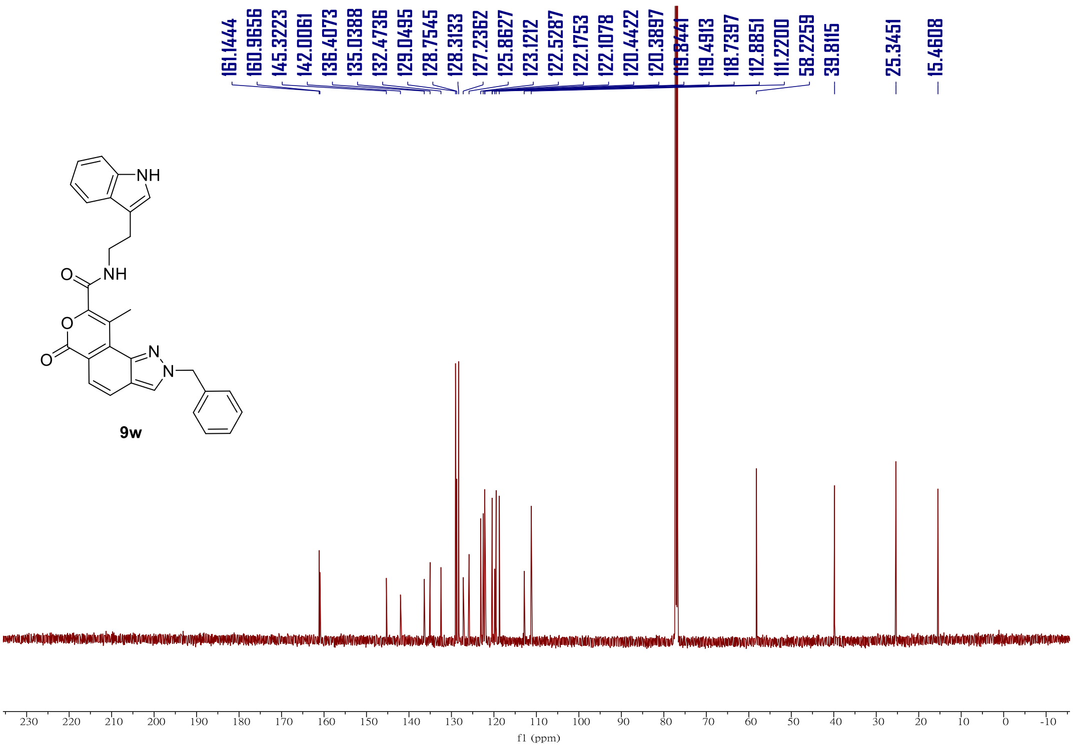

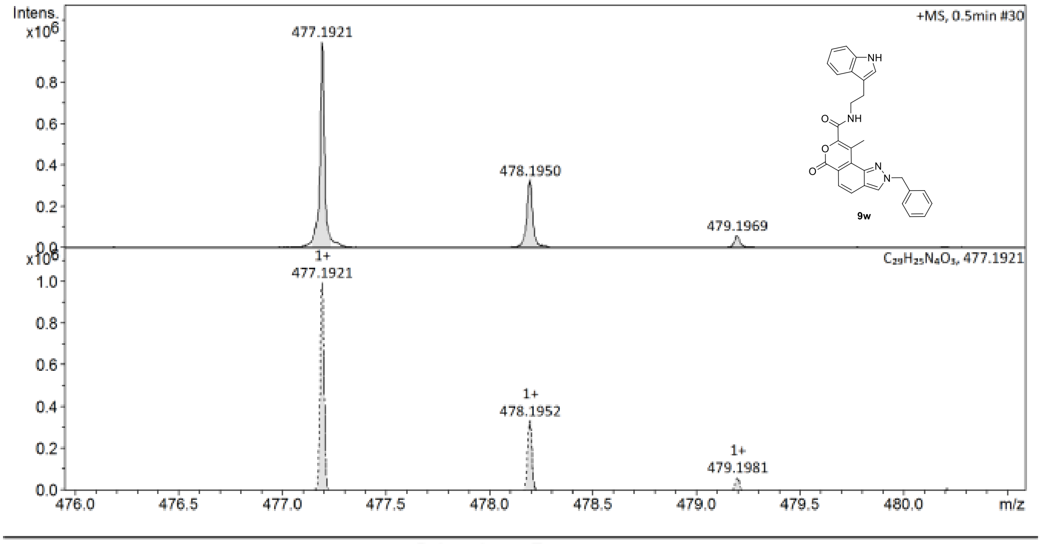

Display Report

Meas. m/z	#	Ion Formula	m/z	err [ppm]	mSigma	# Sigma	Score	rdb	e⁻ Conf	N-Rule	Adduct
477.1921	1	C29H25N4O3	477.1921	0.0	2.7	1	100.00	19.5	even	ok	M+H

Characterization Data of 3a-3z, 3aa, 4n and 3a'

2-benzyl-N-methyl-6-oxo-9-pentyl-2,6-dihydropyrano[3,4-g]indazole-8-carboxamide (3a)

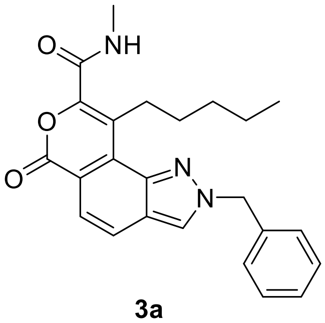

3a

Flash chromatography for purification: hexane/ethyl acetate = 3:1. White solid; Yield = 83 mg (90%); ^{1}H NMR (600 MHz, $CDCl_3$) δ 8.03 (s, 1H), 7.90 (d, J = 8.7 Hz, 1H), 7.75 (d, J = 8.7 Hz, 1H), 7.35 (p, J = 8.1 Hz, 5H), 7.13 (d, J = 5.5 Hz, 1H), 5.64 (s, 2H), 3.94 – 3.86 (m, 2H), 2.98 (d, J = 4.9 Hz, 3H), 1.74 – 1.65 (m, 2H), 1.53 (p, J = 7.5 Hz, 2H), 1.40 – 1.32 (m, 2H), 0.89 (d, J = 7.3 Hz, 3H). $^{13}C\{^1H\}$ NMR (101 MHz, $CDCl_3$) δ 161.5, 161.2, 144.7, 142.0, 135.3, 132.0, 129.1, 128.8, 128.2, 125.9, 125.0, 123.3, 122.6, 120.6, 120.3, 58.3, 32.4, 30.3, 27.2, 26.3, 22.7, 14.4.; HRMS (ESI) m/z: $[M+H]^+$ Calcd for $C_{24}H_{26}N_3O_3$ 404.1969, found 404.1972.

2-(4-bromobenzyl)-N-methyl-6-oxo-9-pentyl-2,6-dihydropyrano[3,4-g]indazole-8-carboxamide (3b)

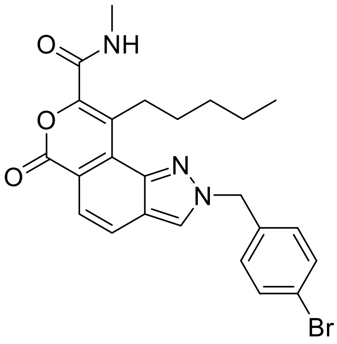

3b

Flash chromatography for purification: hexane/ethyl acetate = 3:1. White solid; Yield = 80 mg (88%); ^{1}H NMR (400 MHz, $CDCl_3$) δ 8.04 (s, 1H), 7.93 (d, J = 8.8 Hz, 1H), 7.77 (s, 1H), 7.49 (d, J = 8.4 Hz, 2H), 7.19 (d, J = 8.4 Hz, 2H), 7.14 – 7.07 (m, 1H), 5.59 (s, 2H), 3.91 – 3.83 (m, 2H), 2.97 (s, 3H), 1.67 (ddt, J = 11.2, 8.0, 3.7 Hz, 2H), 1.53 – 1.45 (m, 2H), 1.33 (dq, J = 14.5, 7.2 Hz, 2H), 0.88 (t, J = 7.3 Hz, 3H). $^{13}C\{^1H\}$ NMR (101 MHz, $CDCl_3$) δ 161.4, 160.7, 144.9, 140.9, 135.2, 132.3, 131.8, 128.8, 126.0, 124.9, 123.8, 122.9, 122.5, 120.9, 119.7, 55.2, 32.4, 30.4, 27.2, 26.3, 22.8, 14.4.; HRMS (ESI) m/z: $[M+Na]^+$ Calcd for $C_{24}H_{24}BrN_3NaO_3$ 504.0893, found 504.0886.

2-(4-methoxybenzyl)-N-methyl-6-oxo-9-pentyl-2,6-dihydropyrano[3,4-g]indazole-8-carboxamide (3c)

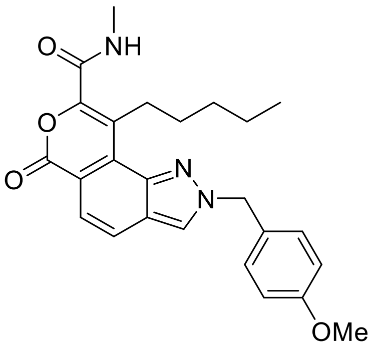

3C

Flash chromatography for purification: hexane/ethyl acetate = 3:1. White solid; Yield = 83 mg (89%); ^{1}H NMR (400 MHz, $CDCl_3$) δ 7.99 (s, 1H), 7.89 (d, *J* = 8.8 Hz, 1H), 7.73 (s, 1H), 7.30 (d, *J* = 8.6 Hz, 2H), 7.13 (d, *J* = 5.0 Hz, 1H), 6.89 (d, *J* = 8.6 Hz, 2H), 5.57 (s, 2H), 3.96 – 3.87 (m, 2H), 3.79 (s, 3H), 2.98 (d, *J* = 4.9 Hz, 3H), 1.75 – 1.66 (m, 2H), 1.55 (dt, *J* = 14.1, 7.1 Hz, 2H), 1.38 (h, *J* = 7.2 Hz, 2H), 0.91 (d, *J* = 7.3 Hz, 3H). $^{13}C\{^1H\}$ NMR (101 MHz, $CDCl_3$) δ 161.5, 161.2, 160.0, 144.6, 141.9, 132.0, 129.8, 127.2, 125.9, 125.0, 123.0, 122.6, 120.6, 120.2, 114.4, 57.9, 55.4, 32.4, 30.3, 27.3, 26.3, 22.8, 14.4.; HRMS (ESI) m/z: $[M+H]^+$ Calcd for $C_{25}H_{28}N_3O_4$ 434.2074, found 434.2075.

N-methyl-6-oxo-9-pentyl-2-(4-(trifluoromethyl)benzyl)-2,6-dihydropyrano[3,4-g]indazole-8-carboxamide (3d)

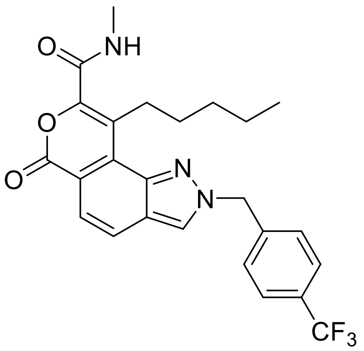

3d

Flash chromatography for purification: hexane/ethyl acetate = 3:1. White solid; Yield = 78 mg (83%); ^{1}H NMR (400 MHz, $CDCl_3$) δ 8.09 (s, 1H), 7.92 (d, *J* = 8.8 Hz, 1H), 7.78 (d, *J* = 8.3 Hz, 1H), 7.61 (d, *J* = 7.9 Hz, 2H), 7.40 (d, *J* = 8.0 Hz, 2H), 7.13 (d, *J* = 21.3 Hz, 1H), 5.67 (s, 2H), 3.85 (d, *J* = 4.7 Hz, 2H), 2.97 (d, *J* = 5.0 Hz, 3H), 1.65 (td, *J* = 7.2, 6.5, 3.7 Hz, 2H), 1.47 (dt, *J* = 14.6, 6.4 Hz, 2H), 1.35 – 1.22 (m, 2H), 0.85 (d, *J* = 7.5 Hz, 3H). $^{13}C\{^1H\}$ NMR (101 MHz, $CDCl_3$) δ 161.4, 161.1, 145.0, 142.1, 139.3, 132.1, 131.5, 131.2, 130.8, 128.2, 127.9, 126.1, 126.1, 126.0, 126.0, 125.2, 124.8, 123.7, 122.5, 122.5, 121.0, 120.6, 57.6, 32.4, 30.3, 27.2, 26.3, 22.7, 14.3.; HRMS (ESI) m/z: $[M+H]^+$ Calcd for $C_{25}H_{25}F_3N_3O_3$ 472.1843, found 472.1840.

2-benzyl-3-methoxy-N-methyl-6-oxo-9-pentyl-2,6-dihydropyrano[3,4-g]indazole-8-carboxamide (3e)

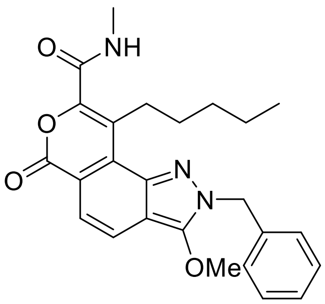

3e

Flash chromatography for purification: hexane/ethyl acetate = 3:1. Yellow solid; Yield = 81 mg (89%); ^{1}H NMR (400 MHz, $CDCl_3$) δ 7.82 (d, *J* = 9.0 Hz, 1H), 7.73 (d, *J* = 8.9 Hz, 1H), 7.33 (d, *J* = 5.1 Hz, 5H), 7.10 (d, *J* = 15.4 Hz, 1H), 5.47 (s, 2H), 4.27 (s, 3H), 3.94 – 3.86 (m, 2H), 2.98 (d, *J* = 4.9 Hz, 3H), 1.74 – 1.65 (m, 2H), 1.55 – 1.47 (m, 2H), 1.35 (dt, *J* = 14.8, 7.3 Hz, 2H), 0.89 (t, *J* = 7.3 Hz, 4H). $^{13}C\{^1H\}$ NMR (101 MHz, $CDCl_3$) δ 161.5, 161.2, 147.0, 143.0, 141.9, 135.7, 131.9, 128.8, 128.3, 128.1, 125.3, 121.8, 120.6, 118.2, 110.3, 61.1, 52.8, 32.4, 30.5, 27.1, 26.2, 22.8, 14.3.; HRMS (ESI) m/z: $[M+H]^+$ Calcd for $C_{25}H_{28}N_3O_4$ 434.2074, found 434.2075.

2-benzyl-3-bromo-N-methyl-6-oxo-9-pentyl-2,6-dihydropyrano[3,4-g]indazole-8-carboxamide (3f)

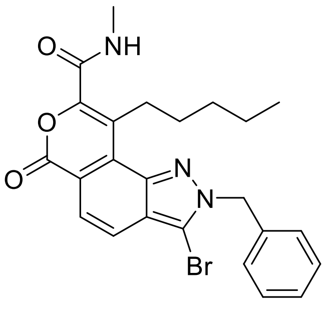

3f

Flash chromatography for purification: hexane/ethyl acetate = 3:1. White solid; Yield = 50 mg (66%); ^{1}H NMR (400 MHz, $CDCl_3$) δ 7.94 (d, *J* = 8.8 Hz, 1H), 7.64 (d, *J* = 8.8 Hz, 1H), 7.39 – 7.29 (m, 5H), 7.16 – 7.06 (m, 1H), 5.71 (s, 2H), 3.91 – 3.79 (m, 2H), 2.98 (d, *J* = 4.9 Hz, 3H), 1.66 (dt, *J* = 11.1, 4.4 Hz, 2H), 1.55 – 1.47 (m, 2H), 1.34 (dt, *J* = 14.8, 7.3 Hz, 2H), 0.88 (t, *J* = 7.3 Hz, 3H). $^{13}C\{^1H\}$ NMR (101 MHz, $CDCl_3$) δ 161.3, 160.9, 144.8, 142.3, 134.7, 132.3, 129.0, 128.7, 128.1, 125.6, 124.7, 121.8, 121.4, 121.2, 107.7, 56.2, 32.4, 30.4, 27.2, 26.3, 22.8, 14.3.; HRMS (ESI) m/z: $[M+H]^+$ Calcd for HRMS (ESI) m/z: $[M+Na]^+$ Calcd for $C_{24}H_{24}BrN_3NaO_3$ 504.0893, found 504.0886.

2-benzyl-N,9-dimethyl-6-oxo-2,6-dihydropyrano[3,4-g]indazole-8-carboxamide (3g)

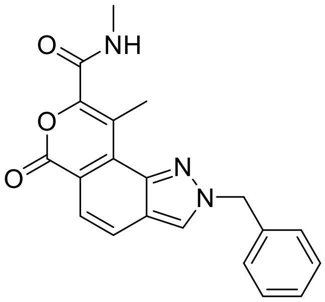

3g

Flash chromatography for purification: hexane/ethyl acetate = 3:1. Yellow solid; Yield = 75 mg (83%); ^{1}H NMR (400 MHz, $CDCl_3$) δ 7.98 (s, 1H), 7.90 (d, *J* = 8.7 Hz, 1H), 7.75 (d, *J* = 8.1 Hz, 1 H), 7.45-7.31 (m, *J* = 4.6 Hz, 5H), 7.11 (s, 1H), 5.66 (s, 2H), 3.31 (s, 3H), 2.98 (d, *J* = 4.8 Hz, 3H). $^{13}C\{^{1}H\}$ NMR (101 MHz, $CDCl_3$) δ 163.7, 160.7, 144.4, 141.9, 135.4, 132.6, 129.1, 128.8, 128.4, 126.0, 123.2, 122.7, 120.5, 119.9, 77.3, 58.3, 26.2, 15.5.; HRMS (ESI) m/z: $[M+H]^+$ Calcd for $C_{20}H_{18}N_3O_3$ 348.1343, found 348.1343.

2-benzyl-N-isobutyl-6-oxo-9-pentyl-2,6-dihydropyrano[3,4-g]indazole-8-carboxamide (3h)

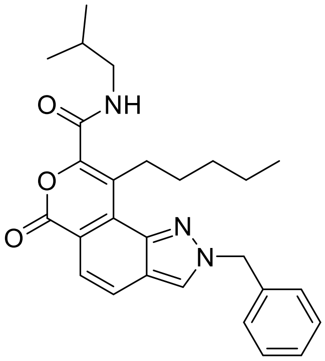

3h

Flash chromatography for purification: hexane/ethyl acetate = 3:1. White solid; Yield = 80 mg (84%); ^{1}H NMR (400 MHz, $CDCl_3$) δ 8.02 (s, 1H), 7.90 (d, *J* = 8.8 Hz, 1H), 7.75 (d, *J* = 8.8 Hz, 1H), 7.39 – 7.30 (m, 5H), 7.11 (d, *J* = 6.2 Hz, 1H), 5.63 (s, 2H), 3.94 – 3.85 (m, 2H), 3.25 (s, 2H), 1.91 (dp, *J* = 13.5, 6.7 Hz, 1H), 1.73 – 1.64 (m, 2H), 1.51 (dt, *J* = 15.8, 7.3 Hz, 2H), 1.34 (dt, *J* = 14.8, 7.4 Hz, 2H), 0.97 (d, *J* = 6.7 Hz, 6H), 0.87 (t, *J* = 7.3 Hz, 3H). $^{13}C\{^{1}H\}$ NMR (101 MHz, $CDCl_3$) δ 161.2, 160.9, 144.7, 142.2, 135.3, 132.1, 129.1, 128.8, 128.2, 125.9, 124.9, 123.3, 122.5, 120.7, 120.3, 58.3, 47.0, 32.4, 30.4, 28.6, 27.3, 22.8, 20.3, 14.3.; HRMS (ESI) m/z: $[M+H]^+$ Calcd for $C_{27}H_{32}N_3O_3$ 446.2439, found 446.2430.

2-benzyl-N-cyclopropyl-9-methyl-6-oxo-2,6-dihydropyrano[3,4-g]indazole-8-carboxamide (3i)

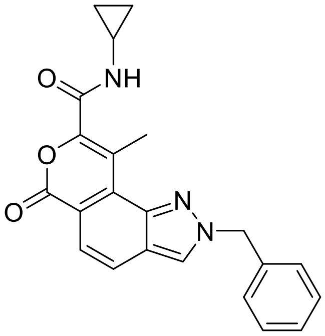

3i

Flash chromatography for purification: hexane/ethyl acetate = 3:1. White solid; Yield = 78 mg (86%); ^{1}H NMR (600 MHz, $CDCl_3$) δ 7.99 (s, 1H), 7.89 (d, *J* = 8.7 Hz, 1H), 7.75 (d, *J* = 8.8 Hz, 1H), 7.37 (p, *J* = 6.9 Hz, 5H), 7.18 (s, 1H), 5.64 (s, 2H), 3.31 (s, 3H), 2.89 (tq, *J* = 7.4, 3.6 Hz, 1H), 0.88 (t, *J* = 6.2 Hz, 2H), 0.70 – 0.63 (m, 2H). $^{13}C\{^{1}H\}$ NMR (101 MHz, $CDCl_3$) δ 162.7, 161.1, 145.4, 141.7, 132.5, 128.8, 128.4, 125.9, 122.7,

120.7, 120.5, 6.6.; HRMS (ESI) m/z: $[M+H]^+$ Calcd for $C_{22}H_{20}N_3O_3$ 374.1500, found 374.1506.

2-benzyl-N-cyclopentyl-6-oxo-9-pentyl-2,6-dihydropyrano[3,4-g]indazole-8-carboxamide (3j)

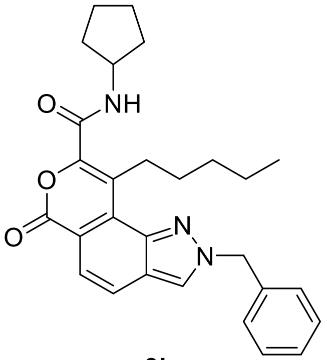

3j

Flash chromatography for purification: hexane/ethyl acetate = 3:1. White solid; Yield = 82 mg (84%); ^{1}H NMR (400 MHz, $CDCl_3$)) δ 8.02 (s, 1H), 7.90 (d, *J* = 8.7 Hz, 1H), 7.75 (d, *J* = 8.7 Hz, 1H), 7.43 – 7.31 (m, 5H), 6.98 (d, *J* = 7.0 Hz, 1H), 5.63 (s, 2H), 4.36 (q, *J* = 7.1 Hz, 1H), 3.88 (dd, *J* = 9.8, 6.0 Hz, 2H), 2.07 (dq, *J* = 12.6, 6.4 Hz, 2H), 1.75 – 1.62 (m, 6H), 1.52 (dd, *J* = 11.8, 6.0 Hz, 4H), 1.35 (p, *J* = 7.4 Hz, 2H), 0.88 (t, *J* = 7.3 Hz, 3H). $^{13}C\{^1H\}$ NMR (101 MHz, $CDCl_3$) δ 161.2, 160.4, 144.7, 143.0, 137.9, 133.2, 129.1, 128.8, 127.5, 125.6, 124.8, 123.3, 122.5, 120.7, 119.8, 57.8, 50.5, 35.5, 32.4, 29.6, 26.5, 23.9, 23.1, 12.3.; HRMS (ESI) m/z: $[M+H]^+$ Calcd for $C_{28}H_{32}N_3O_3$ 458.2439, found 458.2438.

2-benzyl-N-(4-methoxybenzyl)-9-methyl-6-oxo-2,6-dihydropyrano[3,4-g]indazole-8-carboxamide (3k)

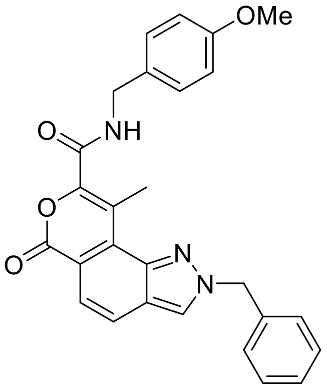

3k

Flash chromatography for purification: hexane/ethyl acetate = 3:1. White solid; Yield = 85 mg (86%); ^{1}H NMR (400 MHz, $CDCl_3$) δ 7.96 (s, 1H), 7.86 (d, *J* = 8.8 Hz, 1H), 7.73 (d, *J* = 8.8 Hz, 1H), 7.42 – 7.29 (m, 7H), 6.87 (d, *J* = 8.6 Hz, 2H), 5.65 (s, 2H), 4.53 (d, *J* = 5.7 Hz, 2H), 3.79 (s, 3H), 3.30 (s, 3H). $^{13}C\{^1H\}$ NMR (101 MHz, $CDCl_3$) δ 161.0, 145.3, 141.8, 135.1, 129.9, 129.5, 129.1, 128.8, 128.4, 125.9, 123.2, 122.7, 120.8, 120.5, 119.9, 114.2, 58.3, 55.4, 43.1, 15.6.; HRMS (ESI) m/z: $[M+H]^+$ Calcd for $C_{27}H_{24}N_3O_4$ 454.1762, found 454.1761.

2-benzyl-9-methyl-6-oxo-N-(4-(trifluoromethyl)benzyl)-2,6-dihydropyrano[3,4-g]indazole-8-carboxamide (3l)

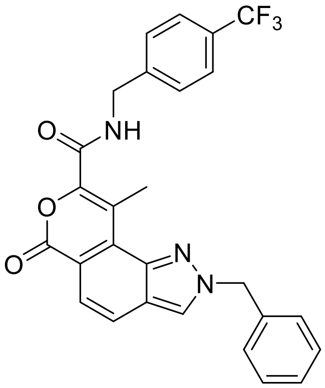

3l

Flash chromatography for purification: hexane/ethyl acetate = 3:1. White solid; Yield = 88 mg (85%); ^{1}H NMR (600 MHz, $CDCl_3$) δ 8.00 (s, 1H), 7.90 (d, *J* = 8.8 Hz, 1H), 7.78 (d, *J* = 8.8 Hz, 1H), 7.61 (d, *J* = 8.0 Hz, 2H), 7.49 (d, *J* = 8.0 Hz, 2H), 7.41 – 7.34 (m, 5H), 5.68 (s, 2H), 4.67 (d, *J* = 6.0 Hz, 2H), 3.33 (s, 3H). $^{13}C\{^1H\}$ NMR (101 MHz, $CDCl_3$) δ 161.3, 161.0, 145.7, 142.0, 141.4, 134.3, 131.3, 129.8, 129.1, 128.9, 128.4, 128.1, 126.0, 125.8, 125.7, 123.3, 123.0, 121.5, 120.5, 120.1, 58.9, 44.5, 19.0.; HRMS (ESI) m/z: $[M+H]^+$ Calcd for $C_{27}H_{21}F_3N_3O_3$ 492.1530, found 492.1529.

2-benzyl-N-(4-chlorobenzyl)-9-methyl-6-oxo-2,6-dihydropyrano[3,4-g]indazole-8-carboxamide (3m)

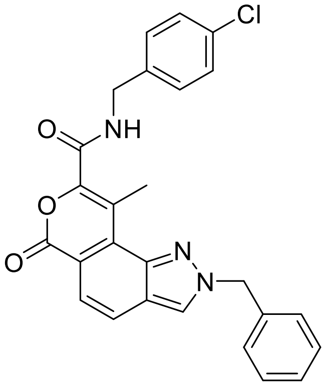

3m

Flash chromatography for purification: hexane/ethyl acetate = 3:1. White solid; Yield = 81 mg (83%); ^{1}H NMR (400 MHz, $CDCl_3$) δ 8.00 (s, 1H), 7.87 (d, *J* = 8.8 Hz, 1H), 7.75 (d, *J* = 8.8 Hz, 1H), 7.52 (d, *J* = 6.0 Hz, 1H), 7.36 (d, *J* = 4.7 Hz, 5H), 7.30 (s, 4H), 5.66 (s, 2H), 4.57 (d, *J* = 6.0 Hz, 2H), 3.31 (s, 3H). $^{13}C\{^1H\}$ NMR (101 MHz, $CDCl_3$) δ 161.2, 161.0, 145.3, 141.5, 136.9, 135.5, 133.5, 132.4, 129.4, 129.1, 128.9, 128.9, 128.4, 126.0, 123.3, 122.9, 121.3, 120.5, 120.0, 58.3, 42.9, 15.6.; HRMS (ESI) m/z: $[M+H]^+$ Calcd for $C_{26}H_{21}ClN_3O_3$ 458.1266, found 458.1264.

2-benzyl-6-oxo-9-pentyl-N-propyl-2,6-dihydropyrano[3,4-g]indazole-8-carboxamide (3n)

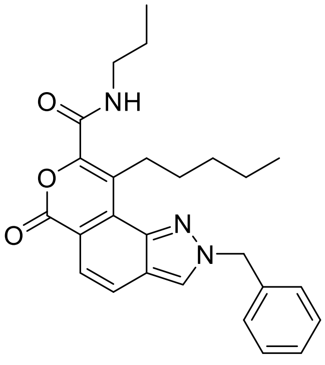

3n

Flash chromatography for purification: hexane/ethyl acetate = 3:1. White solid; Yield = 83 mg (93%); ^{1}H NMR (400 MHz, $CDCl_3$) δ 8.03 (s, 1H), 7.92 (d, J = 8.8 Hz, 1H), 7.77 (d, J = 8.8 Hz, 1H), 7.34 (dtt, J = 7.1, 5.0, 1.8 Hz, 5H), 7.08 (d, J = 6.3 Hz, 1H), 5.64 (s, 2H), 3.96 – 3.83 (m, 2H), 3.46 – 3.34 (m, 2H), 1.66 (dd, J = 15.2, 7.6 Hz, 4H), 1.52 (dt, J = 15.8, 7.3 Hz, 2H), 1.34 (h, J = 7.3 Hz, 2H), 0.98 (t, J = 7.4 Hz, 3H), 0.88 (t, J = 7.3 Hz,

3H). $^{13}C\{^1H\}$ NMR (151 MHz, $CDCl_3$) δ 161.1, 160.7, 144.6, 142.0, 135.1, 132.0, 129.0, 128.7, 128.1, 125.9, 124.9, 123.1, 122.4, 120.6, 120.2, 58.2, 41.2, 32.3, 30.3, 27.2, 22.7, 22.7, 14.2, 11.4.; HRMS (ESI) m/z: $[M+H]^+$ Calcd for $C_{26}H_{30}N_3O_3$ 432.2282, found 432.2280.

2-benzyl-9-methyl-6-oxo-N-propyl-2,6-dihydropyrano[3,4-g]indazole-8-carboxamide (3o)

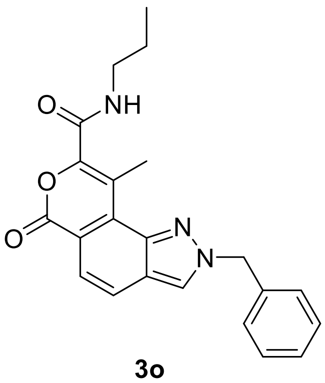

3o

Flash chromatography for purification: hexane/ethyl acetate = 3:1. White solid; Yield = 78 mg (87%); ^1H NMR (600 MHz, $CDCl_3$) δ 7.99 (s, 1H), 7.91 (d, J = 8.8 Hz, 1H), 7.76 (d, J = 8.8 Hz, 1H), 7.37 (q, J = 7.8 Hz, 5H), 7.11 (s, 1H), 5.67 (s, 2H), 3.42 – 3.38 (m, 2H), 3.32 (s, 3H), 1.66 (h, J = 7.4 Hz, 2H), 1.00 (t, J = 7.4 Hz, 3H). $^{13}C\{^1H\}$ NMR (101 MHz, $CDCl_3$) δ 161.2, 161.2, 145.4, 142.0, 135.1, 132.6, 129.7, 128.8, 128.4, 126.0, 123.2, 122.6, 120.5, 119.9, 58.3, 41.4, 22.8, 15.5, 11.5.; HRMS (ESI) m/z: $[M+H]^+$ Calcd for $C_{22}H_{22}N_3O_3$ 376.1656, found 376.1659.

2-benzyl-9-(hydroxymethyl)-8-phenylpyrano[3,4-g]indazol-6(2H)-one (3p)

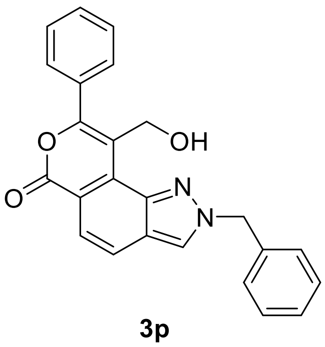

3p

Flash chromatography for purification: hexane/ethyl acetate = 3:1. Yellow solid; Yield = 68 mg (78%); ^1H NMR (600 MHz, $CDCl_3$) δ 8.07 (s, 1H), 7.95 (d, J = 8.7 Hz, 1H), 7.85 – 7.80 (m, 2H), 7.70 (d, J = 8.8 Hz, 1H), 7.50 (d, J = 7.0 Hz, 3H), 7.43 – 7.33 (m, 5H), 5.67 (s, 2H), 4.94 (s, 2H), 3.46 (s, 1H). $^{13}C\{^1H\}$ NMR (151 MHz, $CDCl_3$) δ 162.3, 155.9, 144.0, 134.5, 132.4, 131.7, 130.0, 129.6, 129.2, 129.0, 128.4, 128.3, 124.9, 124.4, 121.2, 120.4, 118.4, 113.4, 77.2, 77.0, 76.8, 59.5, 58.2.; HRMS (ESI) m/z: $[M+H]^+$ Calcd for $C_{24}H_{19}N_2O_3$ 383.1391, found 383.1387.

Methyl 2-benzyl-6-oxo-9-pentyl-2,6-dihydropyrano[3,4-g]indazole-8-carboxylate (3r)

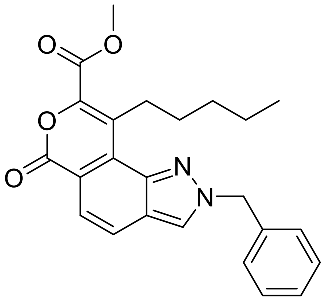

3r

Flash chromatography for purification: hexane/ethyl acetate = 3:1. White solid; Yield = 75 mg (78%); ^{1}H NMR (400 MHz, $CDCl_3$) δ 8.05 (s, 1H), 7.96 (d, *J* = 8.8 Hz, 1H), 7.80 (d, *J* = 8.8 Hz, 1H), 7.40 – 7.31 (m, 5H), 5.65 (s, 2H), 3.94 (s, 3H), 3.78 – 3.70 (m, 2H), 1.72 – 1.65 (m, 2H), 1.51 (dd, *J* = 14.8, 7.6 Hz, 2H), 1.35 (h, *J* = 7.3 Hz, 2H), 0.90 (t, *J* = 7.3 Hz, 3H). $^{13}C\{^1H\}$ NMR (101 MHz, $CDCl_3$) δ 161.6, 161.2, 144.6, 140.4, 135.2, 130.9, 129.1, 128.8, 127.6, 127.0, 125.9, 123.4, 123.2, 121.2, 120.9, 58.4, 52.6, 32.3, 30.0, 27.9, 22.6, 14.3.; HRMS (ESI) m/z: $[M+H]^+$ Calcd for $C_{24}H_{25}N_2O_4$ 405.1809, found 405.1812.

Methyl 2-(4-bromobenzyl)-6-oxo-9-pentyl-2,6-dihydropyrano[3,4-g]indazole-8-carboxylate (3s)

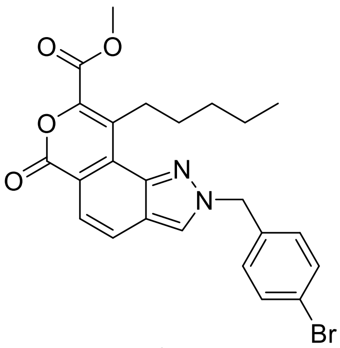

3s

Flash chromatography for purification: hexane/ethyl acetate = 3:1. White solid; Yield = 75 mg (74%); ^{1}H NMR (400 MHz, $CDCl_3$) δ 8.06 (s, 1H), 7.95 (d, *J* = 8.6 Hz, 1H), 7.80 (d, *J* = 8.8 Hz, 1H), 7.48 (s, 2H), 7.19 (d, *J* = 8.0 Hz, 2H), 5.60 (s, 2H), 3.94 (s, 3H), 3.69 (t, *J* = 7.7 Hz, 2H), 1.69 – 1.64 (m, 2H), 1.51 – 1.40 (m, 2H), 1.33 (t, *J* = 7.4 Hz, 2H), 0.89 (t, *J* = 7.2 Hz, 3H). $^{13}C\{^1H\}$ NMR (101 MHz, $CDCl_3$) δ 161.9, 161.1, 145.1, 140.5, 134.2, 132.6, 130.9, 129.8, 126.8, 125.9, 123.5, 123.1, 122.9, 121.4, 121.0, 57.6, 52.7, 32.3, 30.0, 27.8, 22.6, 14.3.; HRMS (ESI) m/z: $[M+H]^+$ Calcd for $C_{24}H_{24}BrN_2O_4$ 483.0914, found 483.0912.

Methyl 2-(4-methoxybenzyl)-6-oxo-9-pentyl-2,6-dihydropyrano[3,4-g]indazole-8-carboxylate (3t)

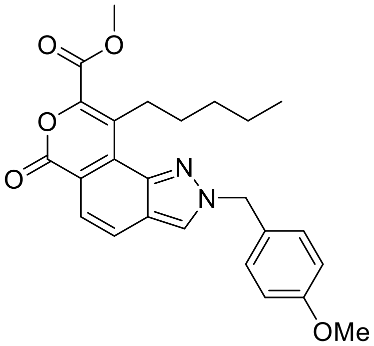

3t

Flash chromatography for purification: hexane/ethyl acetate = 3:1. White solid; Yield = 75 mg (73%); ^{1}H NMR (600 MHz, $CDCl_3$) δ 8.01 (s, 1H), 7.95 (d, *J* = 8.7 Hz, 1H), 7.79 (d, *J* = 8.8 Hz, 1H), 7.30 (d, *J* = 8.7 Hz, 2H),) 6.90 (d, *J* = 8.6 Hz, 2H), 5.58 (s, 2H), 3.95 (s, 3H), 3.80 (s, 3H), 3.77 – 3.72 (m, 2H), 1.72 – 1.66 (m, 2H), 1.56 – 1.51 (m, 2H), 1.38 (dt, *J* =

14.7, 7.4 Hz, 2H), 0.93 (t, *J* = 7.3 Hz, 3H). $^{13}C\{^1H\}$ NMR (101 MHz, $CDCl_3$) δ 161.6, 161.3, 160.0, 144.5, 140.3, 130.9, 129.8, 127.8, 127.1, 125.8, 123.2, 123.1, 121.1, 120.8, 114.4, 57.9, 55.4, 52.6, 32.3, 30.0, 27.9, 22.6, 14.3.; HRMS (ESI) m/z: $[M+H]^+$ Calcd for $C_{25}H_{27}N_2O_5$ 435.1915, found 435.1913.

Methyl 6-oxo-9-pentyl-2-(4-(trifluoromethyl)benzyl)-2,6-dihydropyrano[3,4-g]indazole-8-carboxylate (3u)

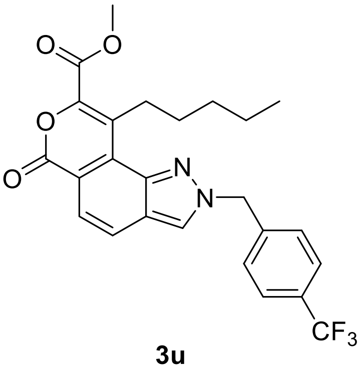

3u

Flash chromatography for purification: hexane/ethyl acetate = 3:1. White solid; Yield = 85 mg (75%); ^1H NMR (400 MHz, $CDCl_3$) δ 8.11 (s, 1H), 7.97 (d, *J* = 8.8 Hz, 1H), 7.82 (d, *J* = 8.8 Hz, 1H), 7.62 (d, *J* = 8.1 Hz, 2H), 7.40 (d, *J* = 8.0 Hz, 2H), 5.71 (s, 2H), 3.94 (s, 3H), 3.68 (d, *J* = 8.0 Hz, 2H), 1.68 – 1.62 (m, 2H), 1.45 (dd, *J* = 15.0, 7.8 Hz, 2H), 1.30 (q, *J* = 7.0 Hz, 2H), 0.85 (t, *J* = 7.3 Hz, 3H). $^{13}C\{^1H\}$ NMR (101 MHz, $CDCl_3$) δ 161.6, 161.1, 144.9, 141.0, 139.2, 131.7, 130.9, 127.8, 126.8, 126.1, 126.0, 125.9, 125.0, 124.2, 123.1, 121.5, 121.2, 57.6, 52.7, 32.2, 29.6, 27.8, 22.9, 14.2.; HRMS (ESI) m/z: $[M+H]^+$ Calcd for $C_{25}H_{24}F_3N_2O_4$ 473.1683, found 473.1680.

Methyl 2-benzyl-3-methoxy-6-oxo-9-pentyl-2,6-dihydropyrano[3,4-g]indazole-8-carboxylate (3v)

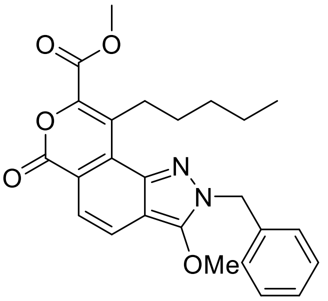

3v

Flash chromatography for purification: hexane/ethyl acetate = 3:1. Yellow solid; Yield = 83 mg (77%); ^1H NMR (400 MHz, $CDCl_3$) δ 7.86 (d, *J* = 8.9 Hz, 1H), 7.78 (d, *J* = 8.9 Hz, 1H), 7.36 – 7.29 (m, 5H), 5.47 (s, 2H), 4.28 (s, 3H), 3.94 (s, 3H), 3.75 – 3.69 (m, 2H), 1.72 – 1.65 (m, 2H), 1.53 – 1.46 (m, 2H), 1.38 – 1.31 (m, 2H), 0.89 (t, *J* = 7.3 Hz, 3H). $^{13}C\{^1H\}$ NMR (101 MHz, $CDCl_3$) δ 161.6, 161.2, 147.1, 142.9, 140.3, 135.7, 130.8, 128.8, 128.3, 128.0, 127.2, 122.4, 121.5, 118.4, 110.3, 61.1, 53.1, 52.6, 32.3, 30.1, 27.8, 22.7, 14.3.; HRMS (ESI) m/z: $[M+H]^+$ Calcd for $C_{25}H_{27}N_2O_5$ 435.1915, found 435.1914.

Ethyl 2-benzyl-9-methyl-6-oxo-2,6-dihydropyrano[3,4-g]indazole-8-carboxylate (3w)

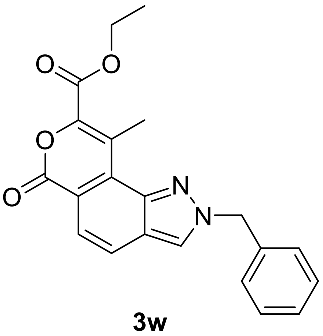

3w

Flash chromatography for purification: hexane/ethyl acetate = 3:1. White solid; Yield = 83 mg (75%); ^{1}H NMR (400 MHz, $CDCl_3$) δ 7.99 (s, 1H), 7.94 (d, *J* = 8.8 Hz, 1H), 7.78 (d, *J* = 8.8 Hz, 1H), 7.40 – 7.31 (m, 5H), 5.67 (s, 2H), 4.42 (q, *J* = 7.2 Hz, 2H), 3.17 (s, 3H), 1.43 (t, *J* = 7.1 Hz, 3H). $^{13}C\{^{1}H\}$ NMR (101 MHz, $CDCl_3$) δ 161.6, 161.3, 146.3, 141.1, 135.6, 132.2, 129.1, 128.8, 128.4, 125.8, 123.9, 123.1, 122.0, 120.7, 62.4, 57.2, 16.0, 13.9.; HRMS (ESI) m/z: $[M+H]^+$ Calcd for $C_{21}H_{19}N_2O_4$ 363.1340, found 363.1345.

2-cyclohexyl-6-oxo-9-pentyl-N-propyl-2,6-dihydropyrano[3,4-g]indazole-8-carboxamide (3x)

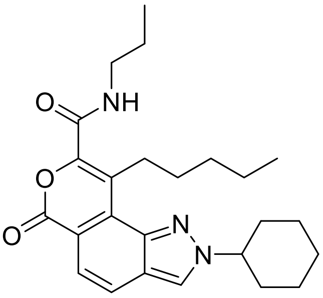

3x

Flash chromatography for purification: hexane/ethyl acetate = 3:1. White solid; Yield = 90 mg (86%); ^{1}H NMR (400 MHz, $CDCl_3$) δ 8.06 (s, 1H), 7.91 (d, *J* = 8.7 Hz, 1H), 7.79 (d, *J* = 8.8 Hz, 1H), 7.10 (t, *J* = 4.6 Hz, 1H), 4.43 (tt, *J* = 11.6, 3.9 Hz, 1H), 3.93 – 3.84 (m, 2H), 3.41 – 3.36 (m, 2H), 2.26 (dd, *J* = 11.1, 4.2 Hz, 2H), 2.01 – 1.92 (m, 4H), 1.84 – 1.68 (m, 4H), 1.67 – 1.48 (m, 6H), 1.43 – 1.36 (m, 2H), 0.98 (t, *J* = 7.4 Hz, 3H), 0.91 (t, *J* = 7.3 Hz, 3H). $^{13}C\{^{1}H\}$ NMR (101 MHz, $CDCl_3$) δ 161.4, 160.9, 144.1, 142.0, 132.0, 125.2, 125.0, 122.6, 121.2, 120.3, 120.0, 63.5, 41.3, 33.8, 32.7, 30.3, 27.5, 25.3, 23.0, 22.8, 14.4, 11.5. HRMS (ESI) m/z: $[M+H]^+$ Calcd for $C_{25}H_{34}N_3O_3$ 424.2595, found 424.2591.

N-methyl-2-(naphthalen-1-ylmethyl)-6-oxo-9-pentyl-2,6-dihydropyrano[3,4-g]indazole-8-carboxamide (3y)

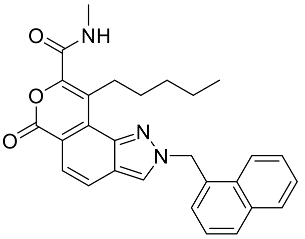

3Y

Flash chromatography for purification: hexane/ethyl acetate = 3:1. White solid; Yield = 86 mg (80%); ^{1}H NMR (400 MHz, $CDCl_3$) δ 8.02 – 7.98 (m, 1H), 7.94 – 7.84 (m, 4H), 7.67 (d, *J* = 8.8 Hz, 1H), 7.53 – 7.45 (m, 4H), 7.11 (d, *J* = 6.7 Hz, 1H), 6.12 (s, 2H), 4.01 – 3.94 (m, 2H), 2.99 (d, *J* = 4.9 Hz, 3H), 1.84 – 1.75 (m, 2H), 1.63 – 1.56 (m, 2H), 1.42 (dq, *J* = 14.4, 7.3 Hz, 2H), 0.91 (t, *J* = 7.3 Hz, 3H). $^{13}C\{^{1}H\}$ NMR (101 MHz,

$CDCl_3$) δ 161.5, 161.2, 144.4, 142.0, 134.0, 132.0, 131.5, 130.4, 130.1, 129.0, 128.2, 127.3, 126.4, 125.9, 125.4, 125.0, 123.3, 123.0, 122.6, 120.6, 120.4, 56.5, 32.4, 30.4, 27.2, 26.3, 22.8, 14.4.; HRMS (ESI) m/z: $[M+Na]^+$ Calcd for $C_{28}H_{27}N_3NaO_3$ 476.1945, found 476.1943.

N-methyl-6-oxo-9-pentyl-2-(pyridin-3-ylmethyl)-2,6-dihydropyrano[3,4-g]indazole-8-carboxamide (3z)

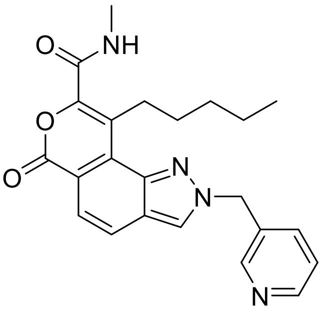

Flash chromatography for purification: hexane/ethyl acetate = 3:1. Yellow solid; Yield = 88 mg (79%). ^1H NMR (400 MHz, $CDCl_3$) δ 8.64 (d, J = 28.3 Hz, 2H), 8.11 (s, 1H), 7.91 (d, J = 8.8 Hz, 1H), 7.77 (d, J = 8.8 Hz, 1H), 7.67 (d, J = 7.9 Hz, 1H), 7.31 (dd, J = 8.0, 4.8 Hz, 1H), 7.15 (d, J = 4.6 Hz, 1H), 5.67 (s, 2H), 3.91 – 3.76 (m, 2H), 2.97 (d, J = 4.9 Hz, 3H), 1.73 – 1.62 (m, 2H), 1.48 (dt, J = 15.7, 7.2 Hz, 2H), 1.32 (dq, J = 14.4, 7.3 Hz, 2H), 0.87 (t, J = 7.3 Hz, 3H). $^{13}C\{^1H\}$ NMR (101 MHz, $CDCl_3$) δ 161.4, 161.1, 149.9, 149.0, 145.0, 142.1, 136.1, 132.0, 131.3, 126.0, 124.7, 124.0, 123.5, 122.5, 121.0, 120.6, 55.6, 32.4, 30.3, 27.2, 26.3, 22.7, 14.3. HRMS (ESI) m/z: $[M+H]^+$ Calcd for $C_{23}H_{25}N_4O_3$ 405.1921, found 405.1923.

2-Benzyl-6-oxo-9-phenyl-N-propyl-2,6-dihydropyrano[3,4-g]indazole-8-carboxamide (3aa)

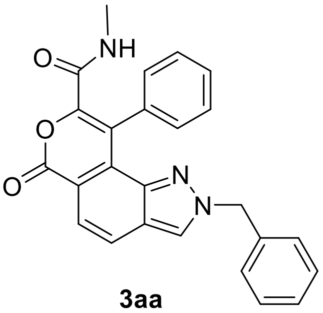

3aa

Flash chromatography for purification: hexane/ethyl acetate = 3:1. Yellow solid; Yield = 78 mg (81%). ^1H NMR (400 MHz, $CDCl_3$) δ 7.93 (d, J = 8.8 Hz, 1H), 7.88 (s, 1H), 7.75 (d, J = 8.8 Hz, 1H), 7.50 – 7.42 (m, 3H), 7.35 (dd, J = 7.6, 1.9 Hz, 2H), 7.32 – 7.27 (m, 3H), 7.12 (dd, J = 6.6, 2.9 Hz, 2H), 6.83 (t, J = 6.0 Hz, 1H), 3.26 – 3.17 (m, 2H), 1.48 (p, J = 7.4 Hz, 2H), 0.87 (t, J = 7.4 Hz, 4H). $^{13}C\{^1H\}$ NMR (101 MHz, $CDCl_3$) δ 161.0, 159.5, 144.3, 143.1, 135.8, 134.9, 131.4, 129.6, 128.8, 128.8, 128.6, 127.9, 127.7, 125.8, 122.9, 122.6, 122.1, 120.4, 119.2, 58.2, 41.3, 22.7, 11.4. HRMS (ESI) m/z: $[M+Na]^+$ Calcd for $C_{27}H_{23}N_3NaO_3$ 460.1632, found 460.1632.

(E)-1-oxo-1-(propylamino)oct-2-en-2-yl 2-benzyl-2H-indazole-6-carboxylate (4n)

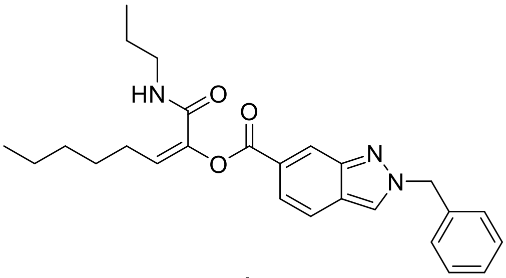

4n

Flash chromatography for purification: hexane/ethyl acetate = 3:1. White solid; Yield = 9 mg (8%) ^{1}H NMR (600 MHz, $CDCl_3$) δ 8.57 (s, 1H), 7.95 (s, 1H), 7.72 – 7.67 (m, 2H), 7.39 – 7.30 (m, 5H), 5.76 (s, 1H), 5.65 (s, 2H), 5.50 (s, 1H), 3.28 (q, *J* = 6.8 Hz, 2H), 3.00 – 2.95 (m, 2H), 1.57 (dq, *J* = 21.6, 7.4 Hz, 5H), 1.39 – 1.29 (m, 4H), 0.94 (t, *J* = 7.4 Hz, 3H), 0.86 (t, *J* = 7.1 Hz, 3H). $^{13}C\{^1H\}$ NMR (151 MHz, $CDCl_3$) δ 164.9, 164.6, 163.6, 147.8, 135.0, 129.0, 128.7, 128.1, 127.0, 124.4, 123.3, 122.1, 121.5, 120.5, 112.0, 58.0, 41.2, 31.4, 30.6, 26.5, 22.8, 22.4, 13.9, 11.4. HRMS (ESI) m/z: $[M+H]^+$ Calcd for $C_{26}H_{32}N_3O_3$ 434.2439, found 434.2440.

1-benzyl-N-methyl-8-oxo-5-pentyl-1,8-dihydropyrano[4,3-f]indazole-6-carboxamide (3a')

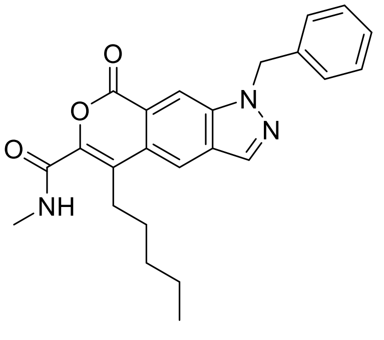

3a′

Flash chromatography for purification: hexane/ethyl acetate = 3:1. White solid; Yield = 88 mg (82%) ^{1}H NMR (400 MHz, $CDCl_3$) δ 8.49 (s, 1H), 8.29 (s, 1H), 8.19 (s, 1H), 7.33 – 7.27 (m, 3H), 7.26 – 7.21 (m, 2H), 7.14 – 7.01 (m, 1H), 5.70 (s, 2H), 3.42 – 3.32 (m, 2H), 2.96 (d, *J* = 5.0 Hz, 3H), 1.72 – 1.64 (m, 3H), 1.52 (dt, *J* = 14.0, 6.9 Hz, 2H), 1.40 (h, *J* = 7.2 Hz, 2H), 0.92 (t, *J* = 7.2 Hz, 3H). $^{13}C\{^1H\}$ NMR (101 MHz, $CDCl_3$) δ 161.6, 161.3, 138.8, 138.4, 135.9, 134.5, 129.0, 128.8, 128.3, 127.4, 123.7, 120.7, 118.2, 111.6, 53.6, 32.3, 30.0, 26.2, 25.6, 22.7, 14.2.; HRMS (ESI) m/z: $[M+H]^+$ Calcd for $C_{24}H_{26}N_3O_3$ 404.1969, found 404.1973.

Spectral Data of 3a-3z, 3aa, 4n and 3a'

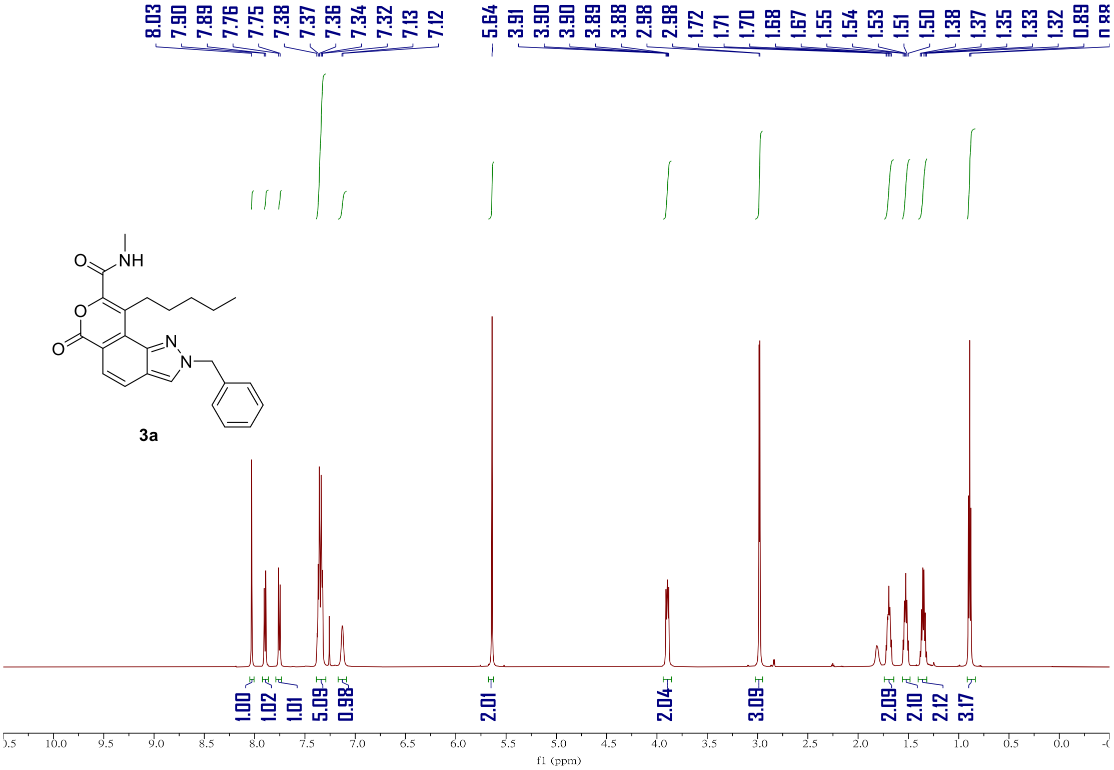

^{1}H spectrum (600 MHz) of compound **3a** in $CDCl_3$

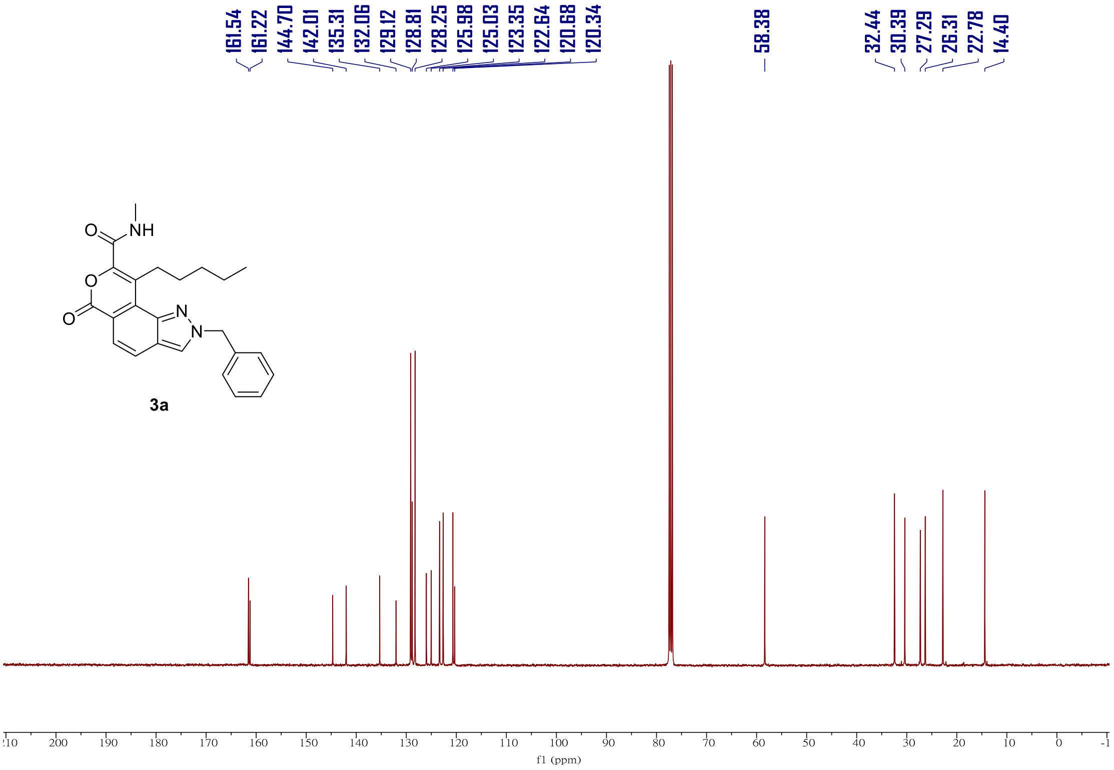

$^{13}C\{^{1}H\}$ spectrum (101 MHz) of compound **3a** in $CDCl_3$

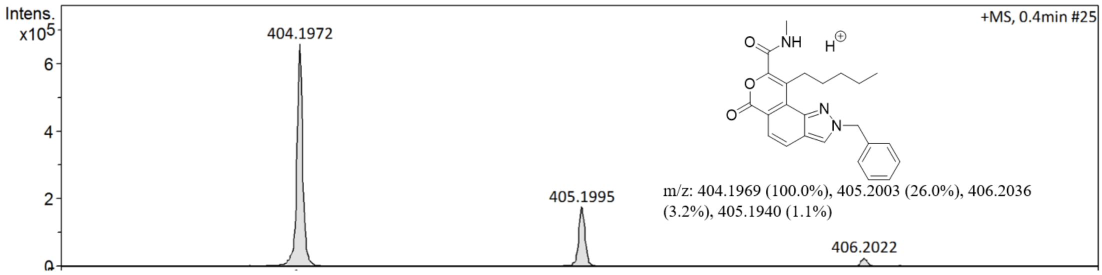

Display Report

Meas. m/z	#	Ion Formula	m/z	err [ppm]	mSigma	# Sigma	Score	rdb	e⁻ Conf	N-Rule	Adduct
404.1972	1	C24H26N3O3	404.1969	0.9	4.4	1	100.00	13.5	even	ok	M+H

HRMS Mass (ESI) spectrum of compound **3a**

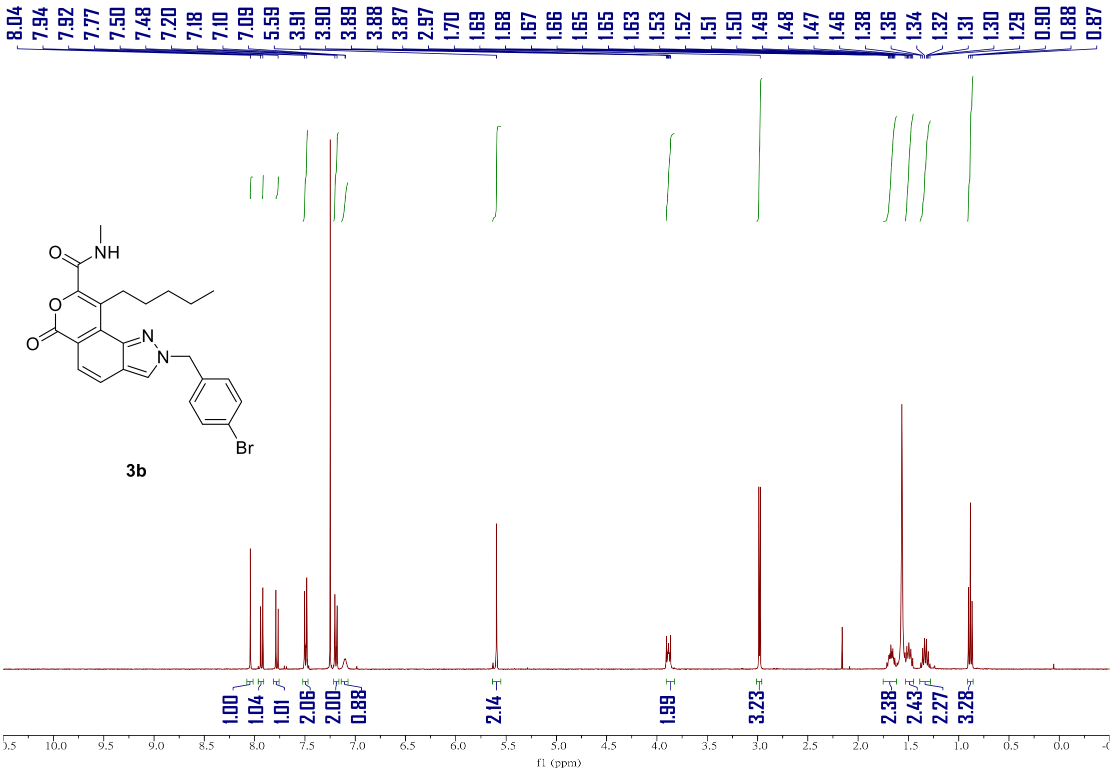

^{1}H spectrum (400 MHz) of compound **3b** in $CDCl_3$

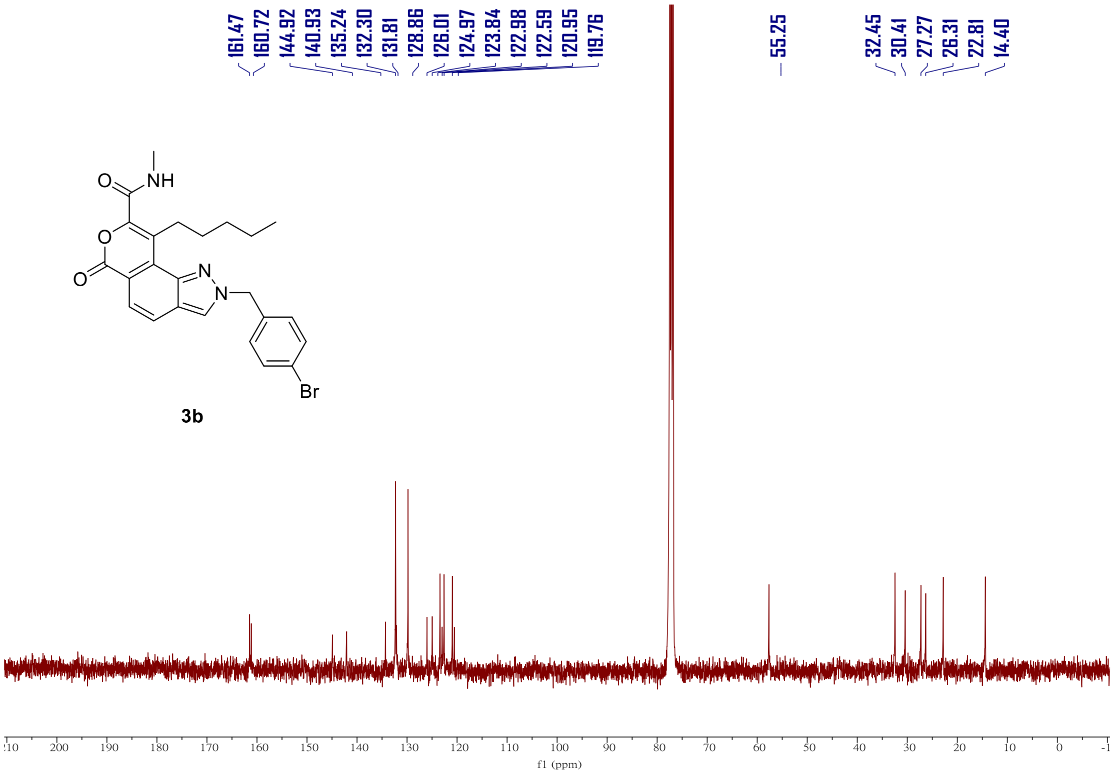

$^{13}C\{^{1}H\}$ spectrum (400 MHz) of compound **3b** in $CDCl_3$

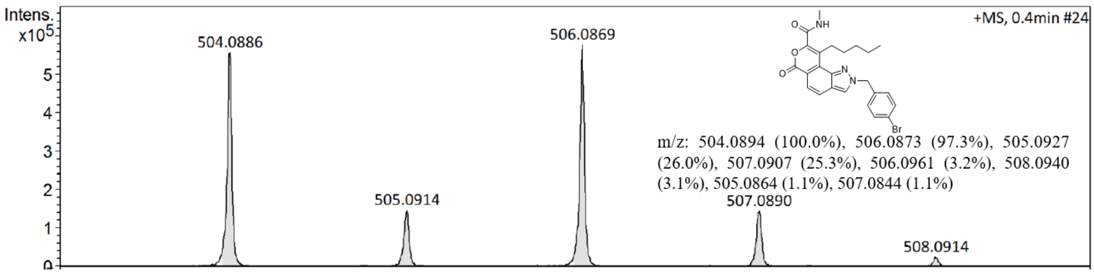

Display Report

Meas. m/z	#	Ion Formula	m/z	err [ppm]	mSigma	# Sigma	Score	rdb	e⁻ Conf	N-Rule	Adduct	Adduct
504.0886	1	C24H24BrN3NaO3	504.0893	1.4	12.5	1	100.00	13.5	even	ok	M+Na	M+Na

HRMS Mass (ESI) spectrum of compound **3b**

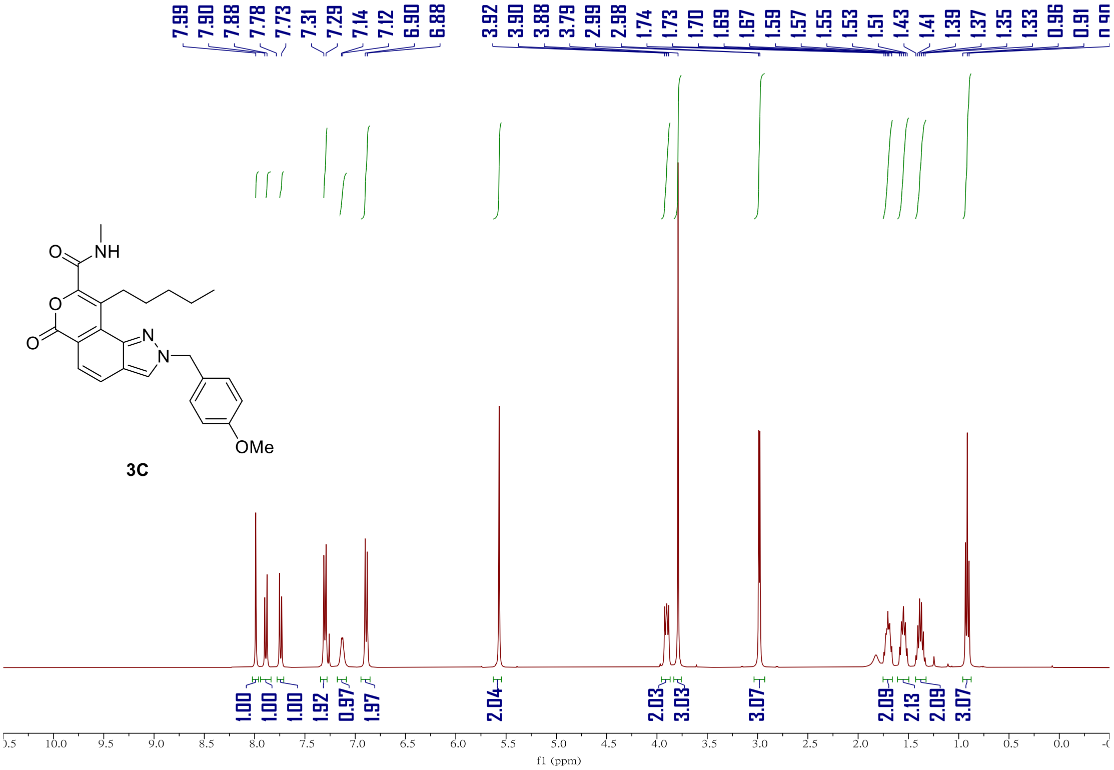

^{1}H spectrum (400 MHz) of compound **3c** in $CDCl_3$

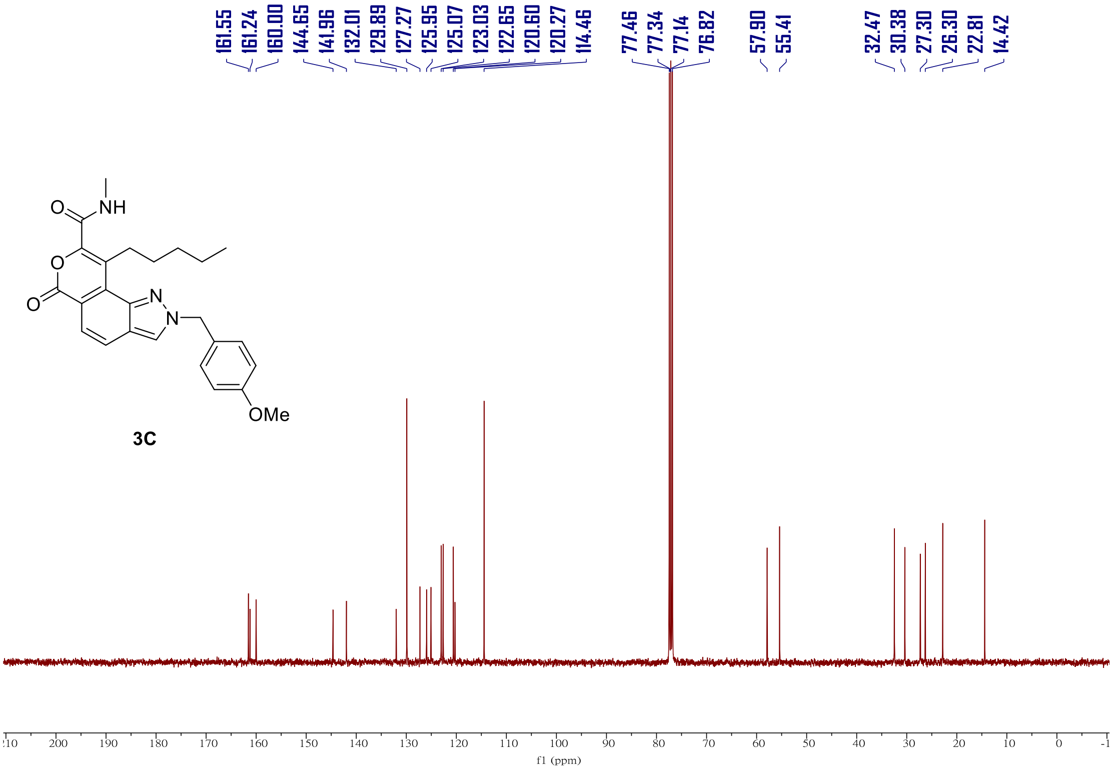

$^{13}C\{^{1}H\}$ spectrum (101 MHz) of compound **3c** in $CDCl_3$

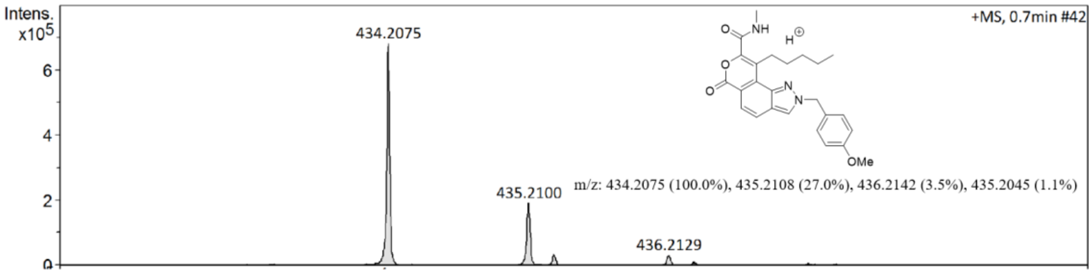

Display Report

Meas. m/z	#	Ion Formula	m/z	err [ppm]	mSigma	# Sigma	Score	rdb	e⁻ Conf	N-Rule	Adduct
434.2075	1	C25H28N3O4	434.2074	0.2	3.6	1	100.00	13.5	even	ok	M+H

HRMS Mass (ESI) spectrum of compound **3c**

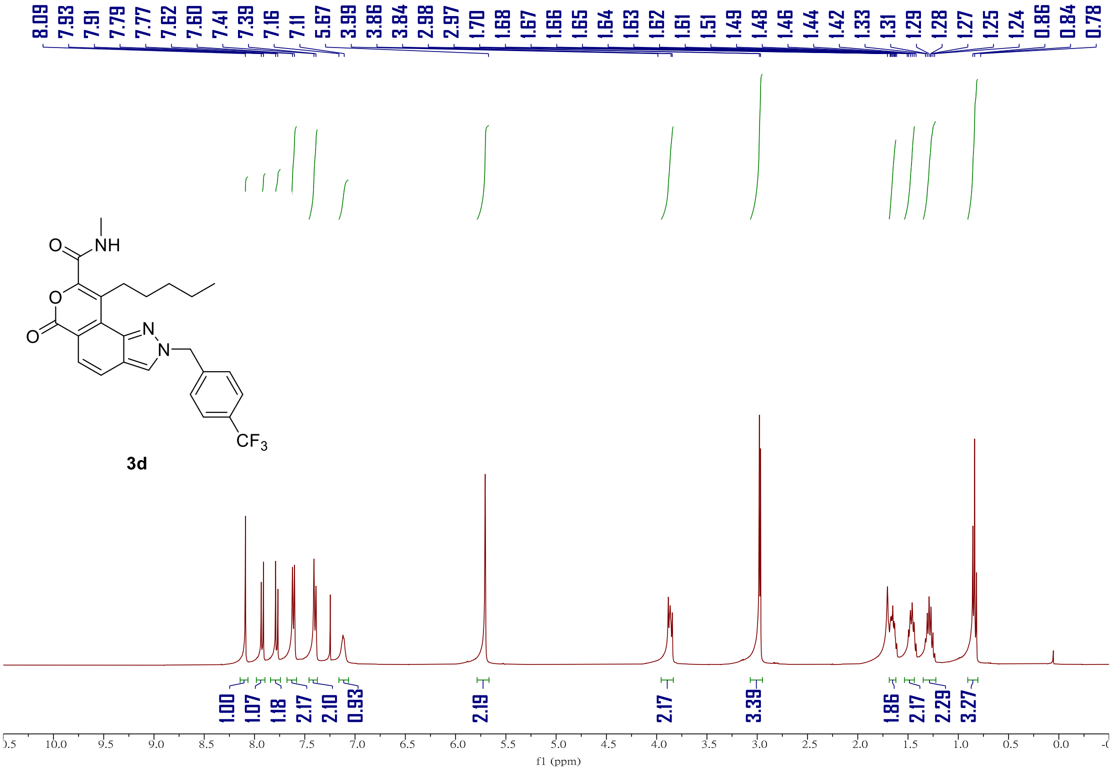

^{1}H spectrum (400 MHz) of compound **3d** in $CDCl_3$

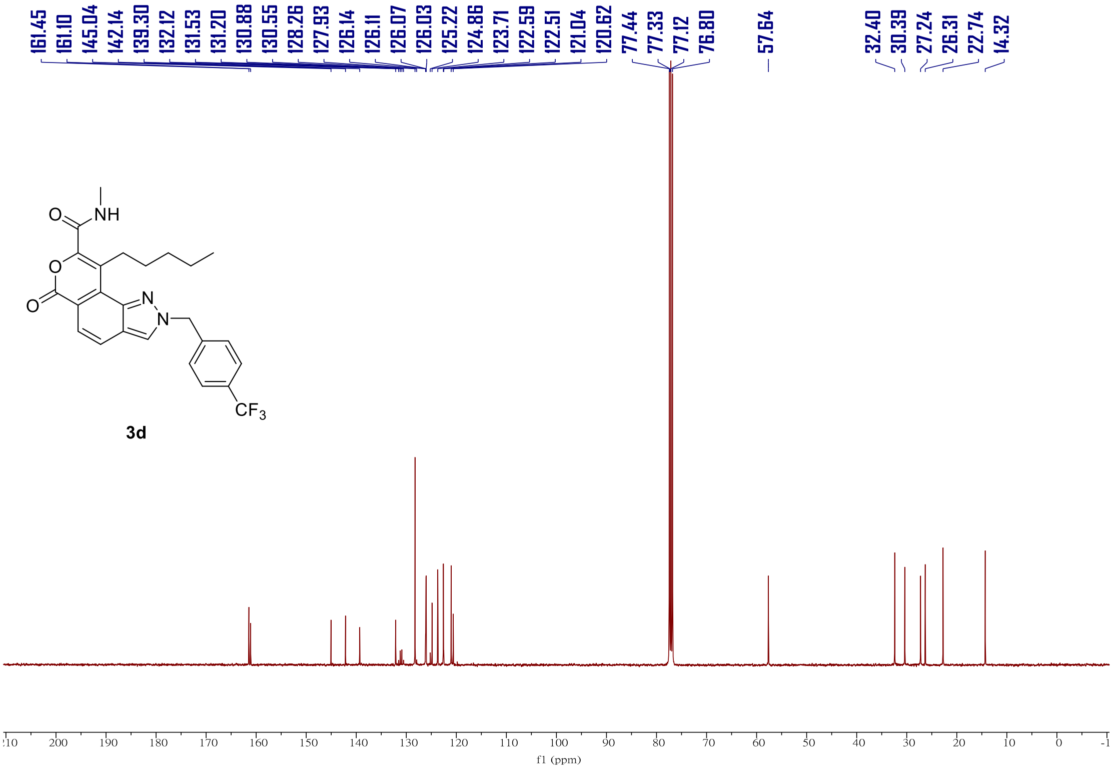

$^{13}C\{^{1}H\}$ spectrum (101 MHz) of compound **3d** in $CDCl_3$

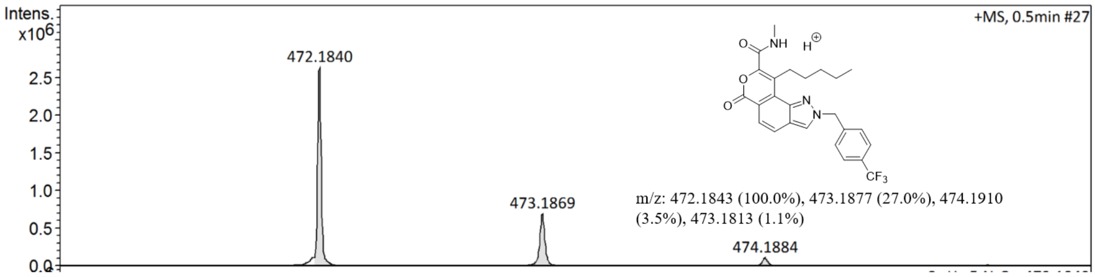

Display Report

Meas. m/z	#	Ion Formula	m/z	err [ppm]	mSigma	# Sigma	Score	rdb	e⁻ Conf	N-Rule	Adduct
472.1840	1	C25H25F3N3O3	472.1843	-0.6	11.1	1	100.00	13.5	even	ok	M+H

HRMS Mass (ESI) spectrum of compound **3d**

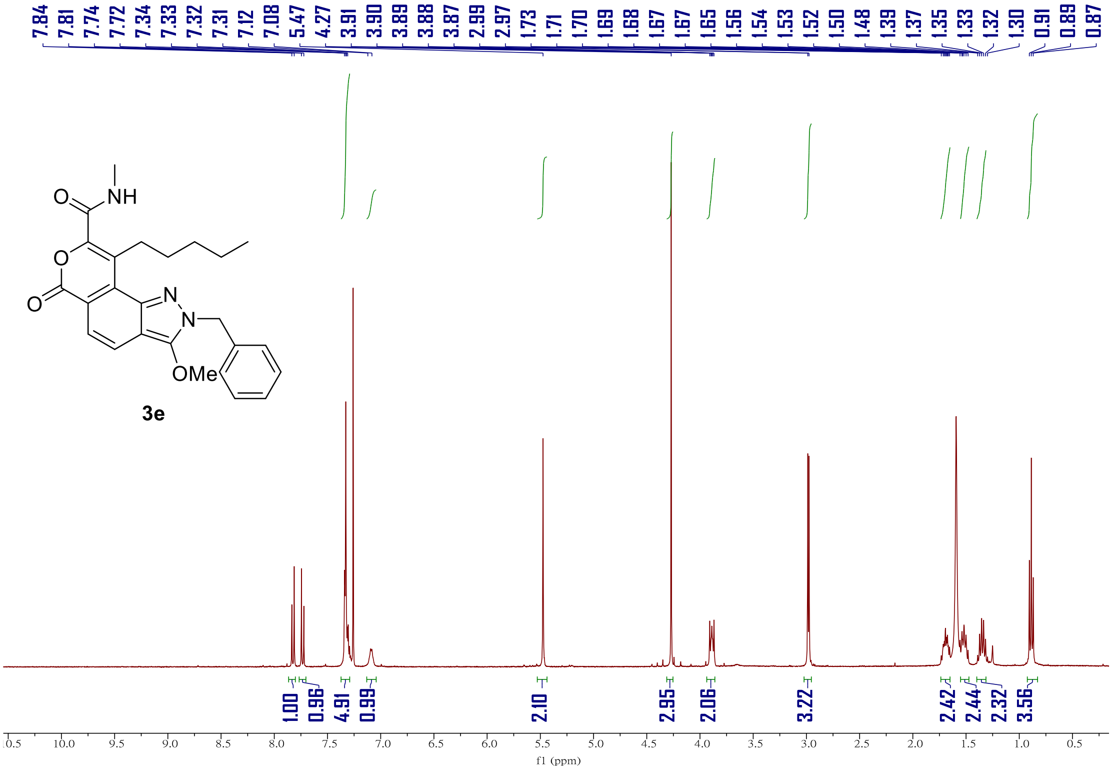

^{1}H spectrum (400 MHz) of compound **3e** in $CDCl_3$

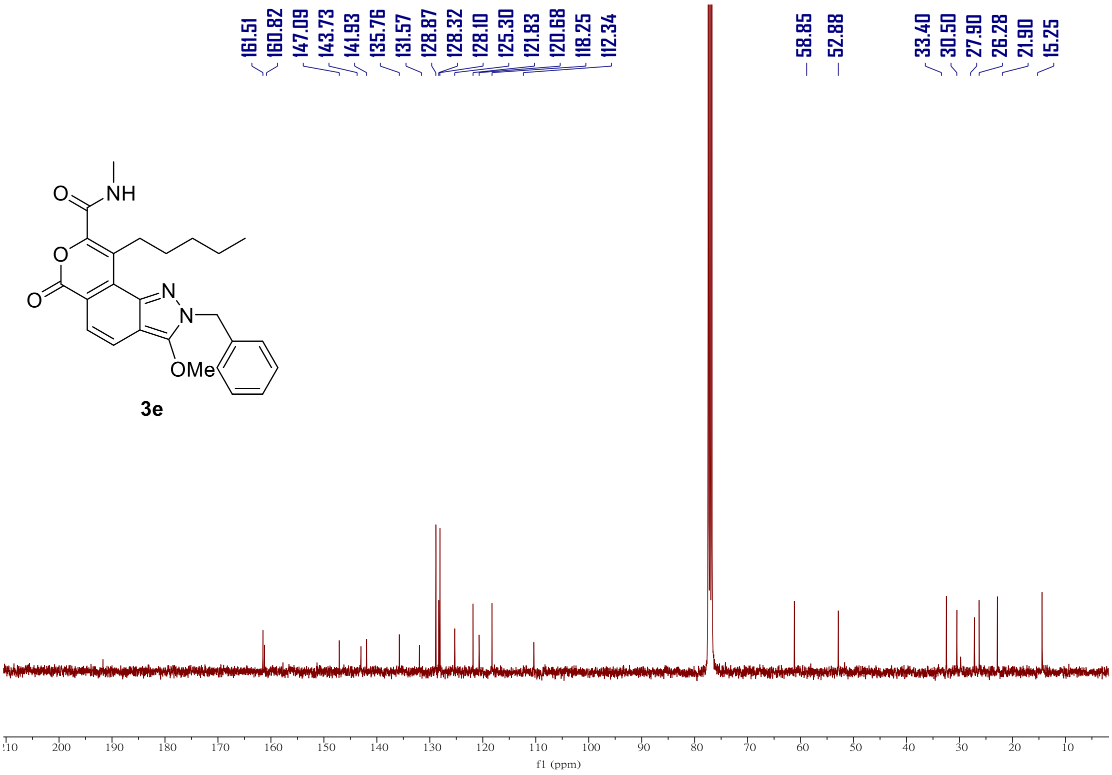

^{13}C{^{1}H} spectrum (101 MHz) of compound **3e** in $CDCl_3$

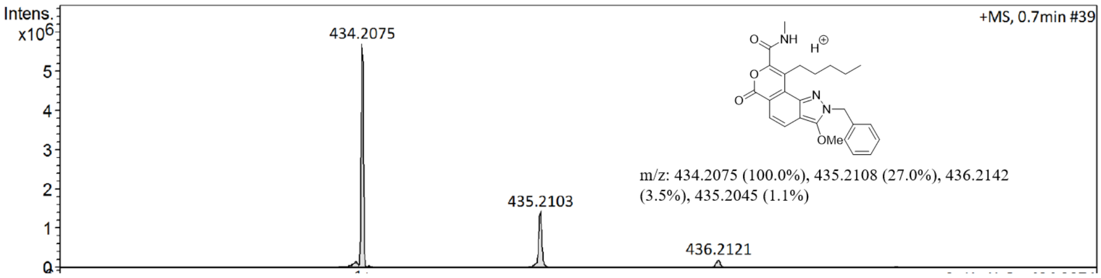

Display Report

Meas. m/z	#	Ion Formula	m/z	err [ppm]	mSigma	# Sigma	Score	rdb	e⁻ Conf	N-Rule	Adduct
434.2075	1	C25H28N3O4	434.2074	-0.1	18.9	1	100.00	13.5	even	ok	M+H

HRMS Mass (ESI) spectrum of compound **3e**

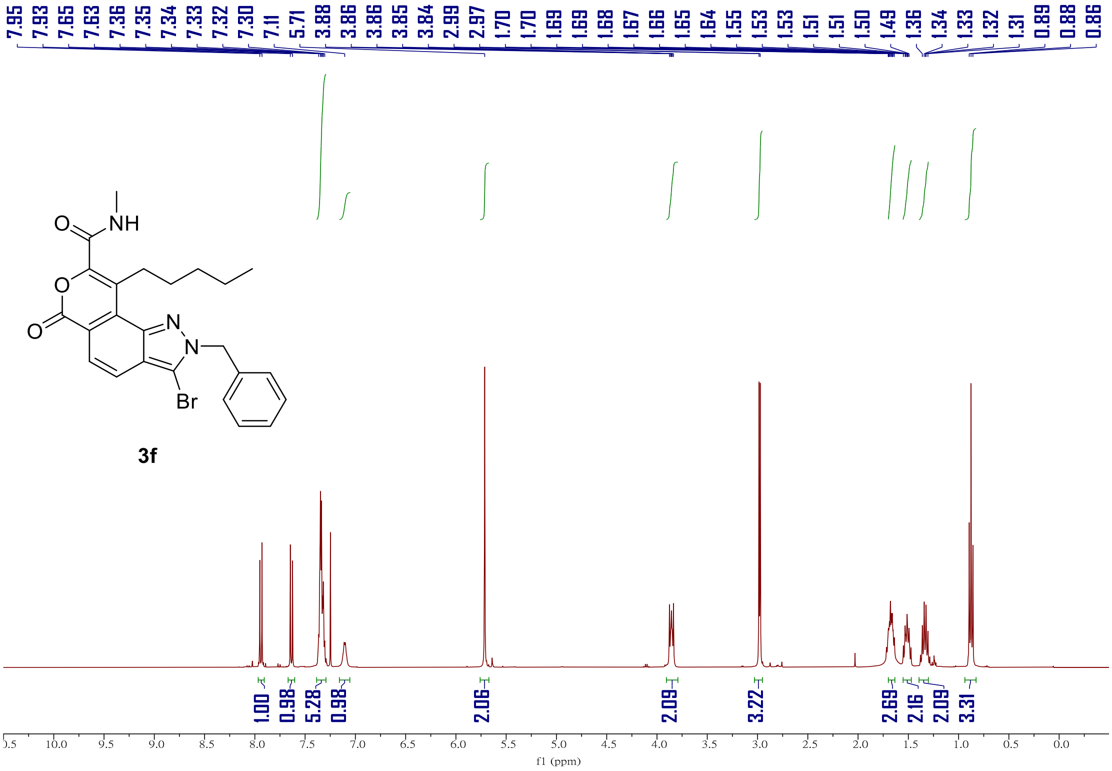

^{1}H spectrum (400 MHz) of compound **3f** in $CDCl_3$

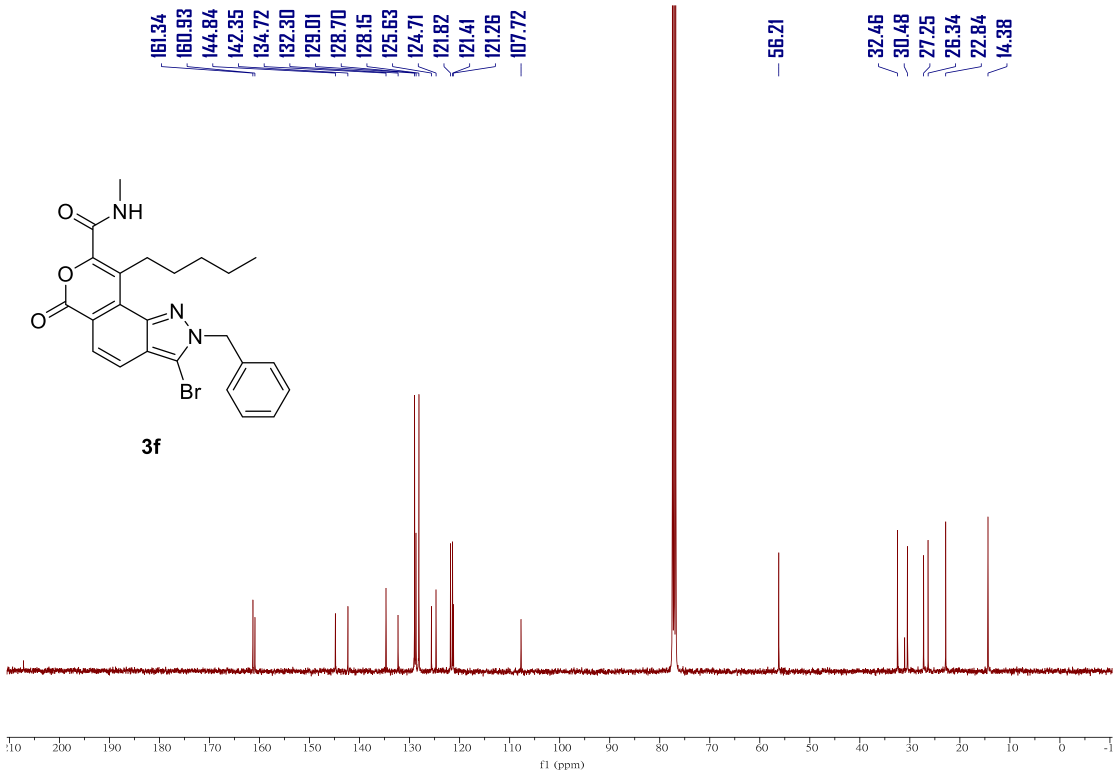

$^{13}C\{^{1}H\}$ spectrum (101MHz) of compound **3f** in $CDCl_3$

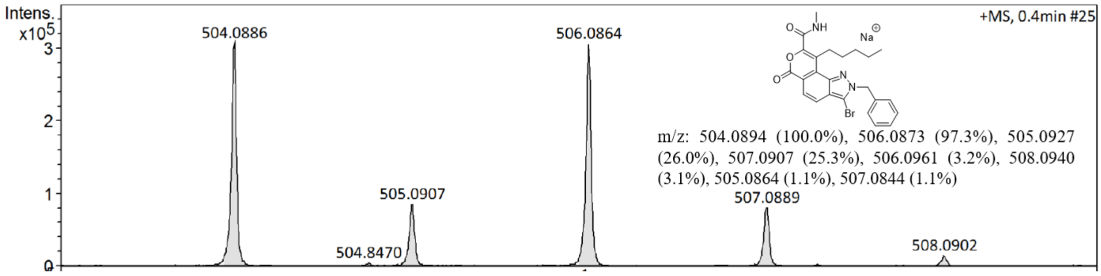

Display Report

Meas. m/z	#	Ion Formula	m/z	err [ppm]	mSigma	# Sigma	Score	rdb	e⁻ Conf	N-Rule	Adduct
504.0886	1	C24H24BrN3NaO3	504.0893	1.5	7.5	1	100.00	13.5	even	ok	M+Na

HRMS Mass (ESI) spectrum of compound **3f**

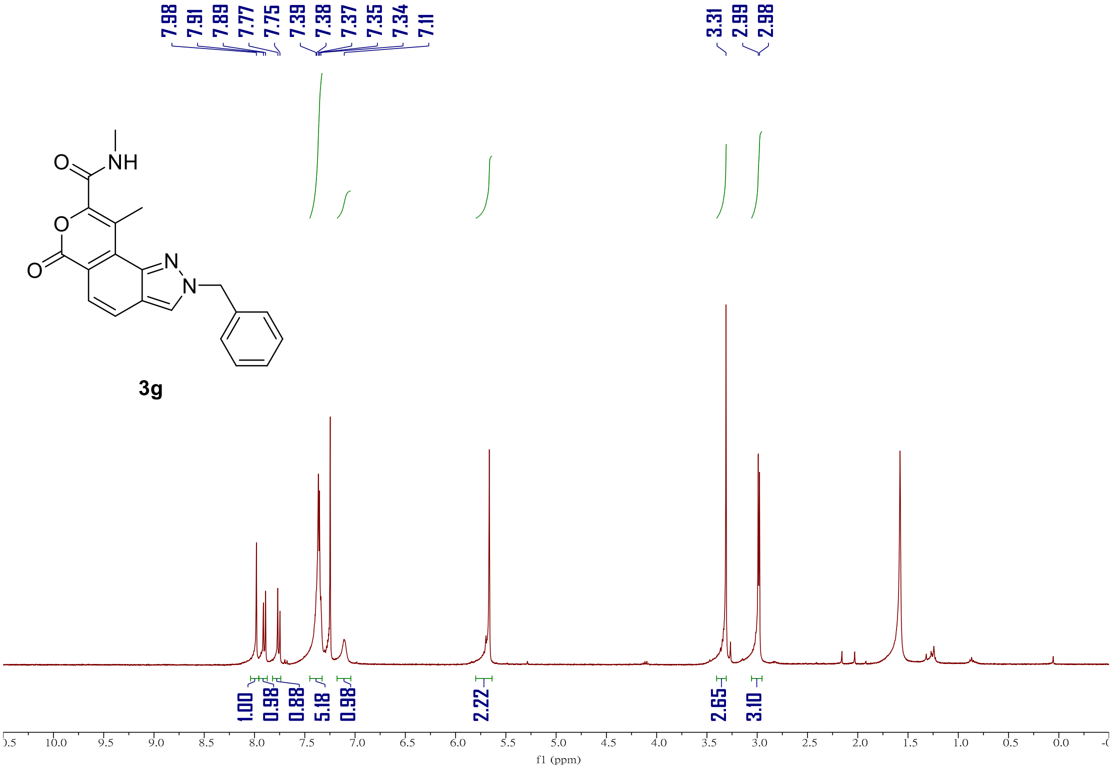

^{1}H spectrum (400 MHz) of compound **3g** in $CDCl_3$

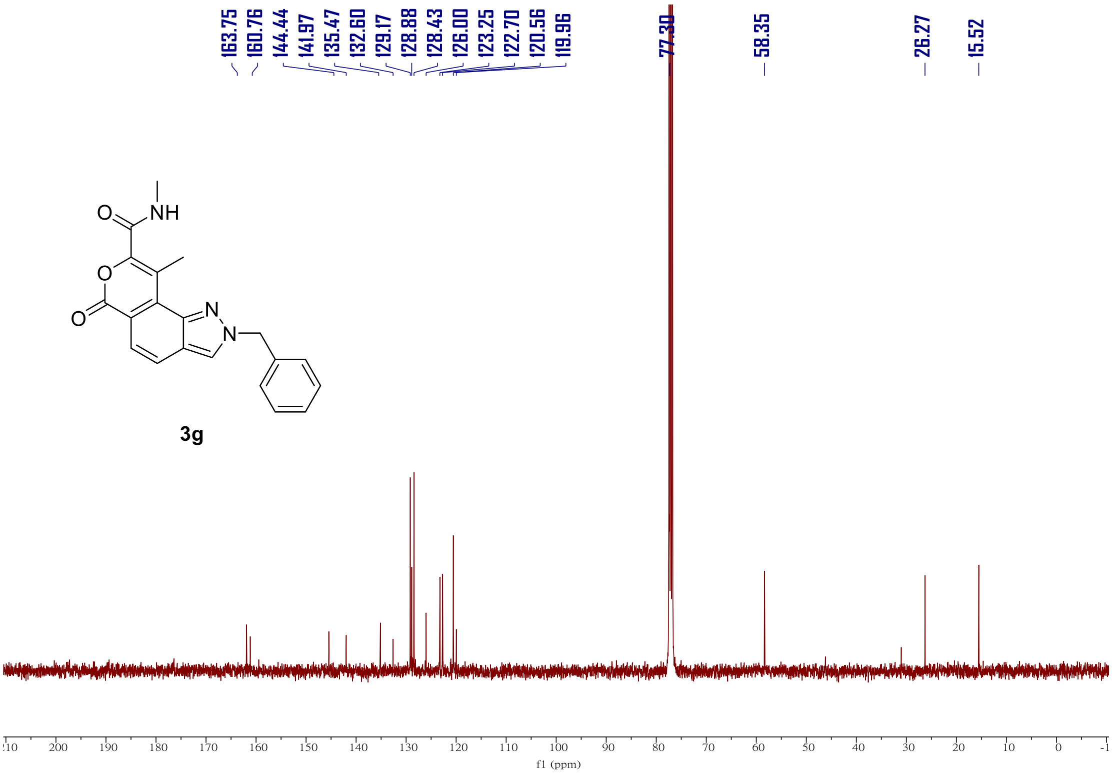

$^{13}C\{^{1}H\}$ spectrum (400 MHz) of compound **3g** in $CDCl_3$

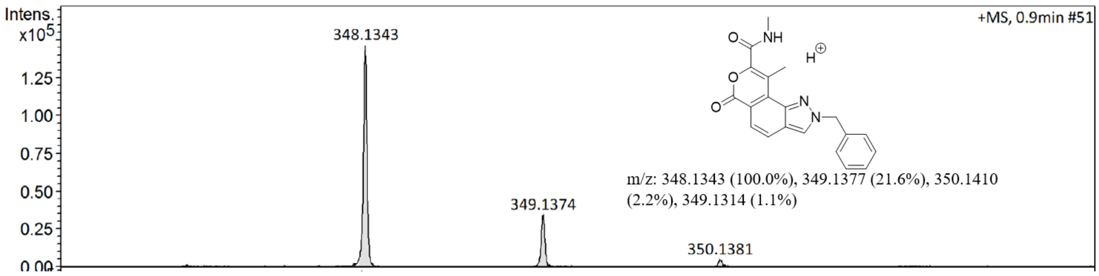

Display Report

Meas. m/z	#	Ion Formula	m/z	err [ppm]	mSigma	# Sigma	Score	rdb	e⁻ Conf	N-Rule	Adduct
348.1343	1	C20H18N3O3	348.1343	-0.1	2.4	1	100.00	13.5	even	ok	M+H

HRMS Mass (ESI) spectrum of compound **3g**

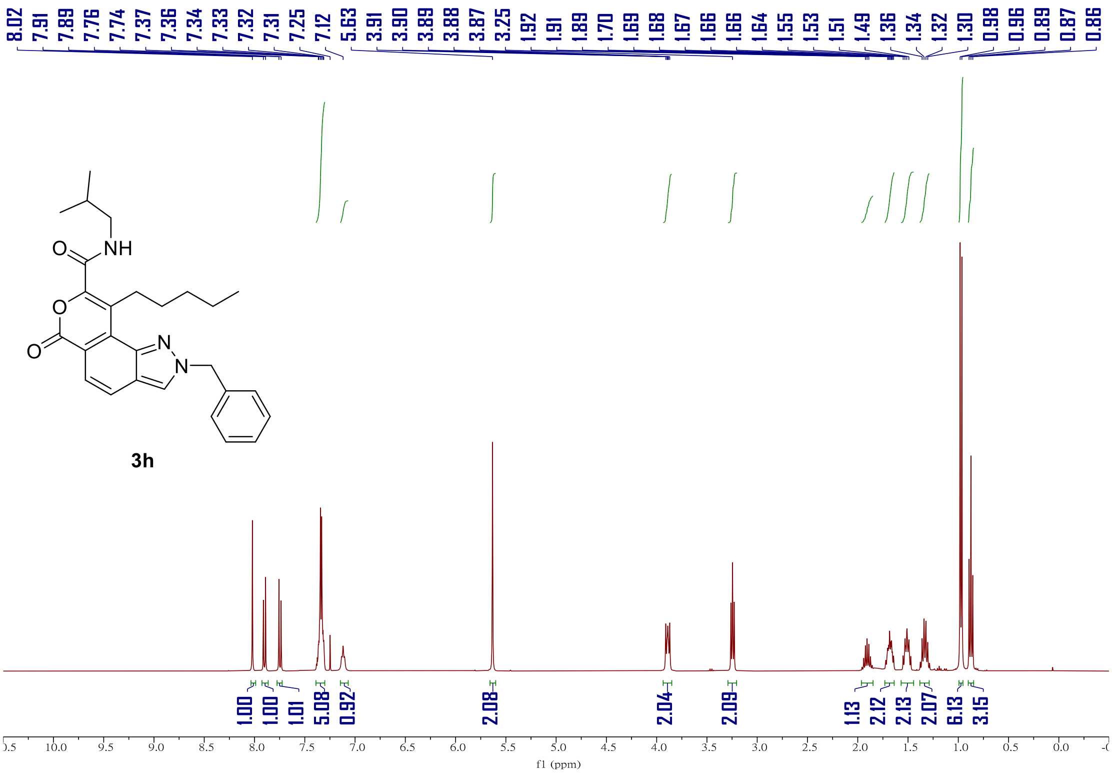

^{1}H spectrum (400 MHz) of compound **3h** in $CDCl_3$

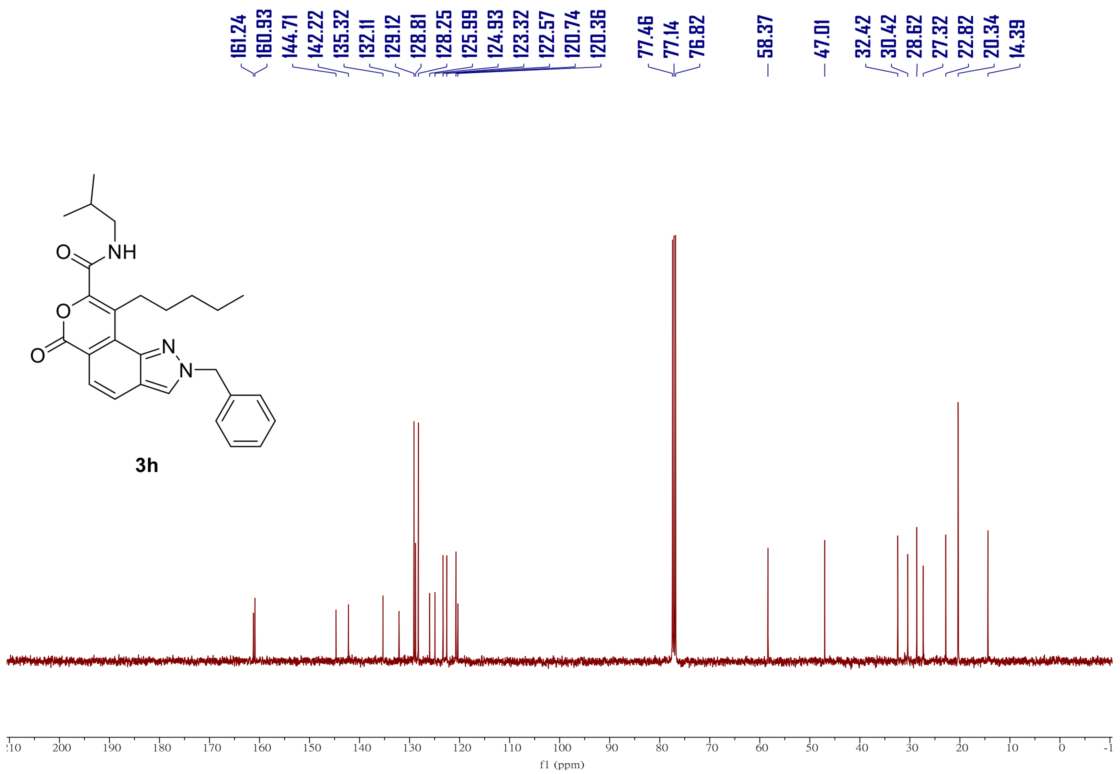

$^{13}C\{^1H\}$ spectrum (101 MHz) of compound **3h** in $CDCl_3$

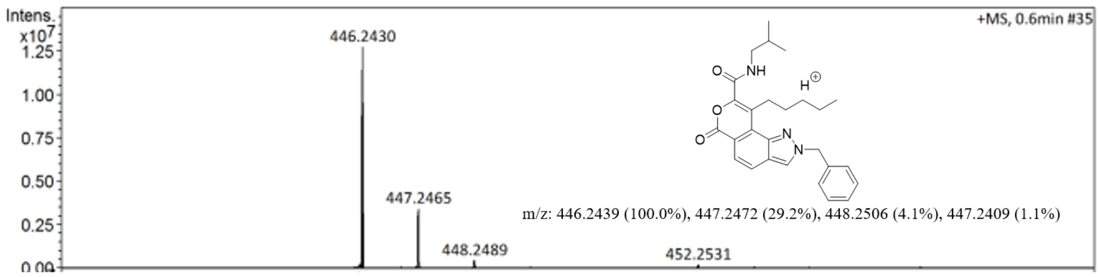

Display Report

Meas. m/z	#	Ion Formula	m/z	err [ppm]	mSigma	# Sigma	Score	rdb	e⁻ Conf	N-Rule	Adduct
446.2430	1	C27H32N3O3	446.2438	1.8	21.0	1	100.00	13.5	even	ok	M+H

HRMS Mass (ESI) spectrum of compound **3h**

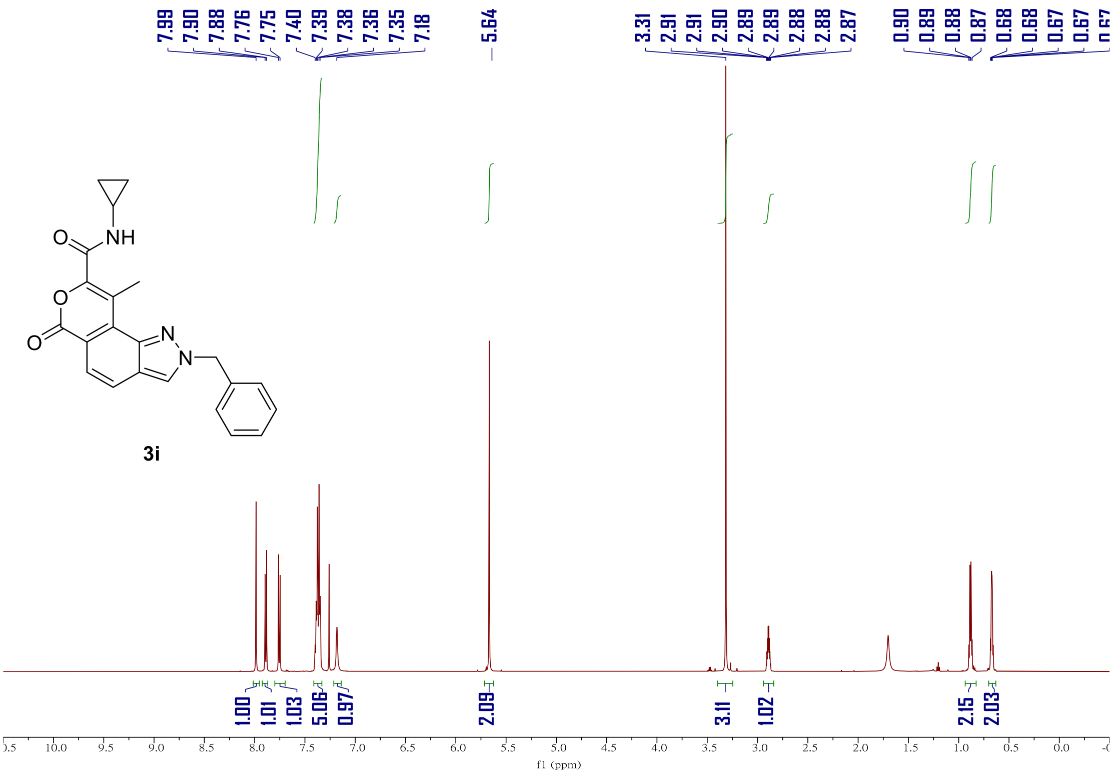

^{1}H spectrum (600 MHz) of compound **3i** in $CDCl_3$

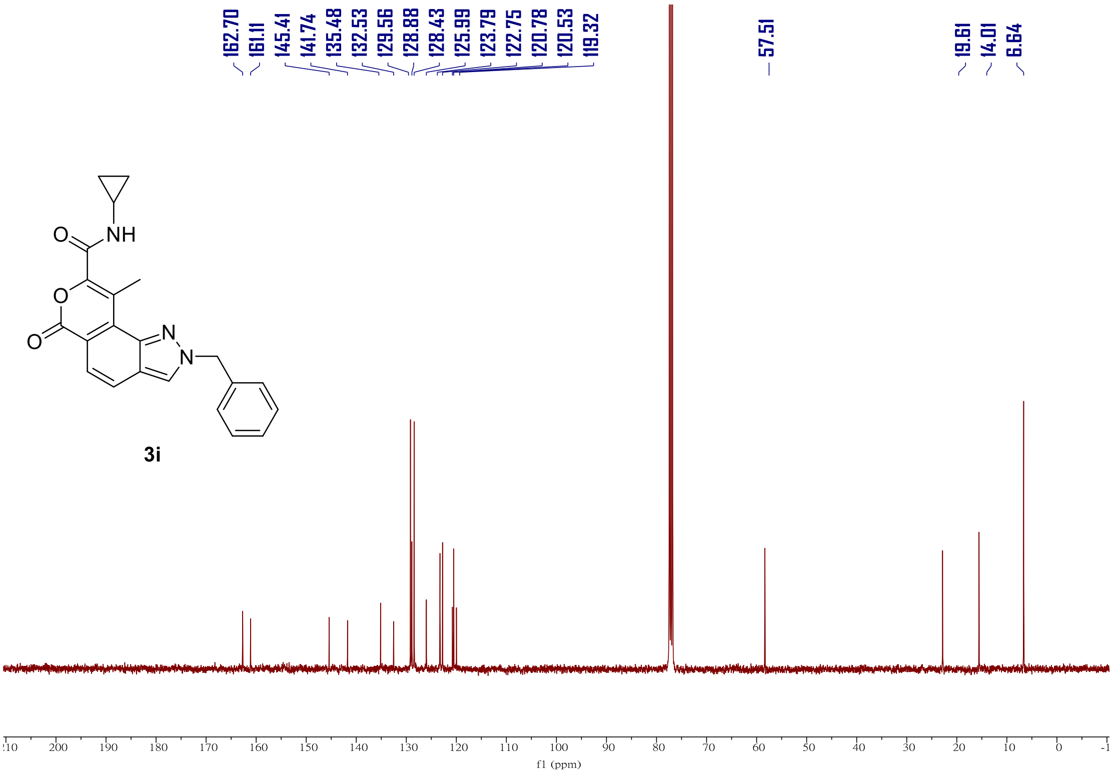

$^{13}C\{^{1}H\}$ spectrum (101 MHz) of compound **3i** in $CDCl_3$

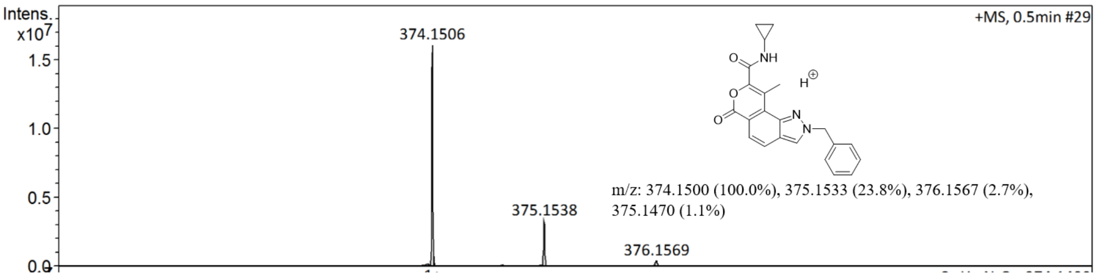

Display Report

Meas. m/z	#	Ion Formula	m/z	err [ppm]	mSigma	# Sigma	Score	rdb	e⁻ Conf	N-Rule	Adduct
374.1506	1	C22H20N3O3	374.1499	1.9	23.8	1	100.00	14.5	even	ok	M+H

HRMS Mass (ESI) spectrum of compound **3i**

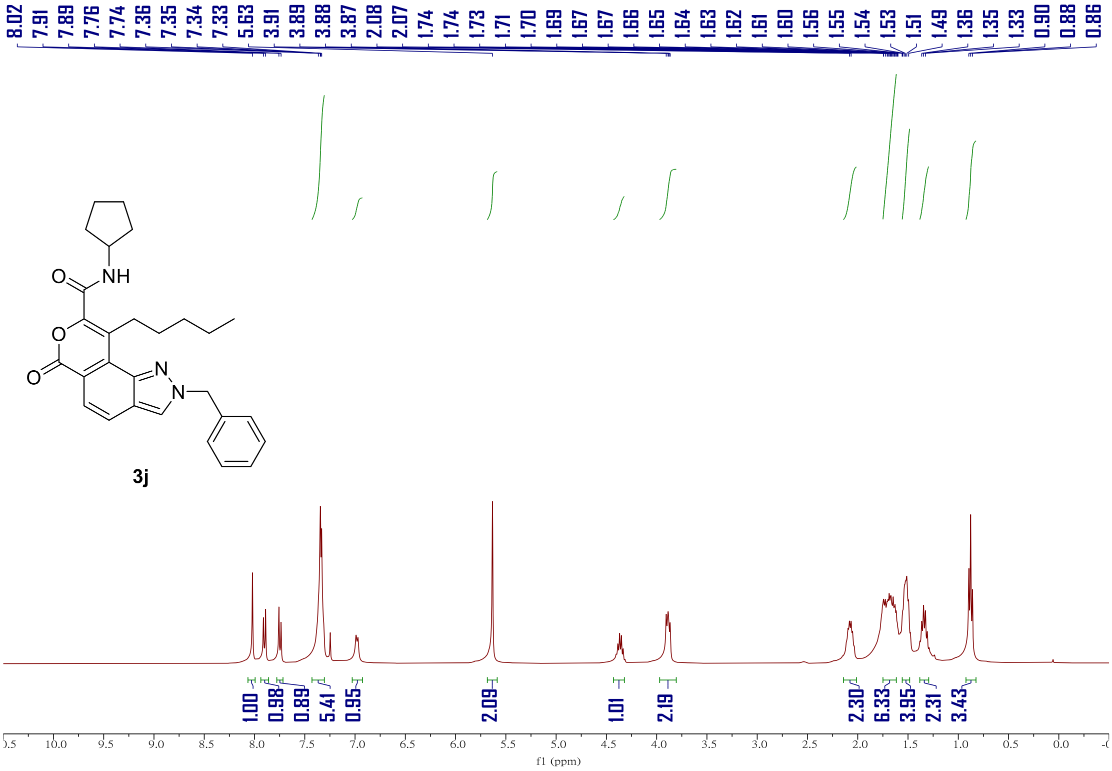

^{1}H spectrum (600 MHz) of compound **3j** in $CDCl_3$

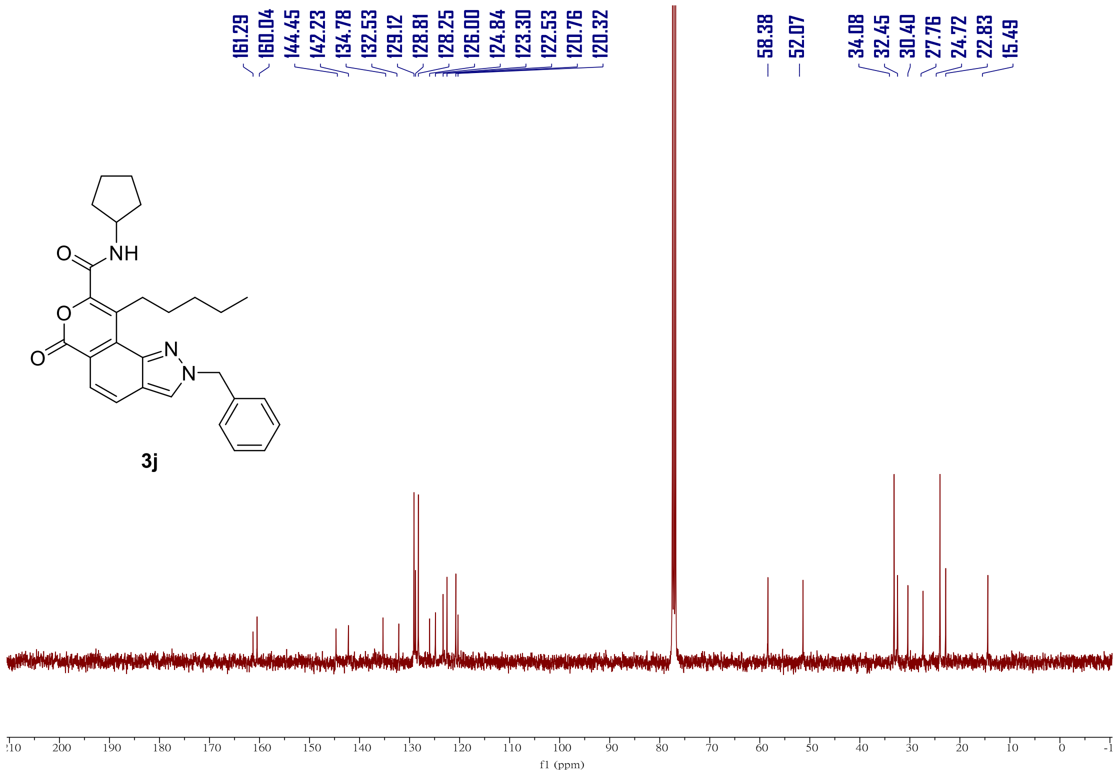

$^{13}C\{^{1}H\}$ spectrum (101 MHz) of compound **3j** in $CDCl_3$

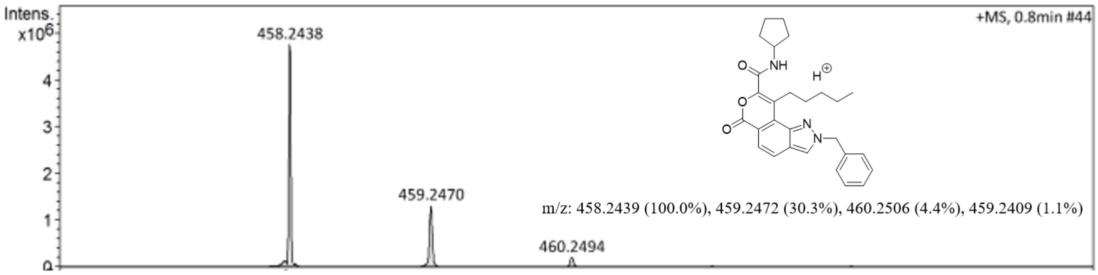

Display Report

Meas. m/z	#	Ion Formula	m/z	err [ppm]	mSigma	# Sigma	Score	rdb	e⁻ Conf	N-Rule	Adduct
458.2438	1	C28H32N3O3	458.2438	-0.1	20.7	1	100.00	14.5	even	ok	M+H

HRMS Mass (ESI) spectrum of compound **3j**

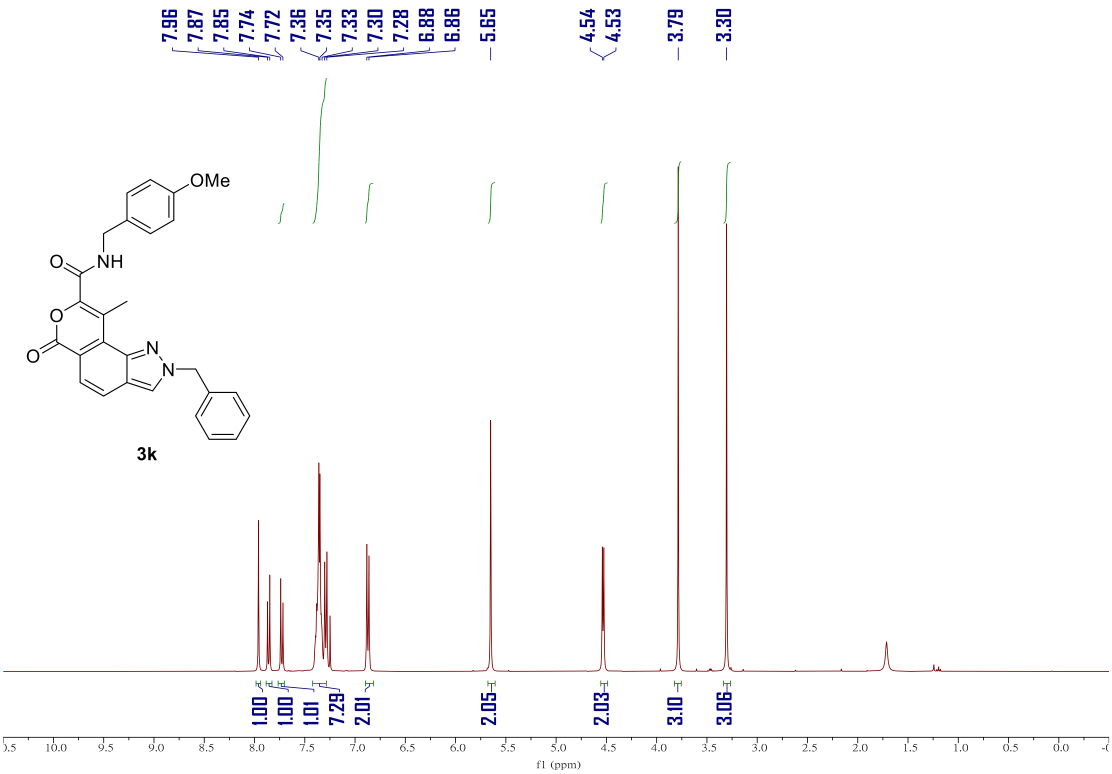

^{1}H spectrum (400 MHz) of compound **3k** in $CDCl_3$

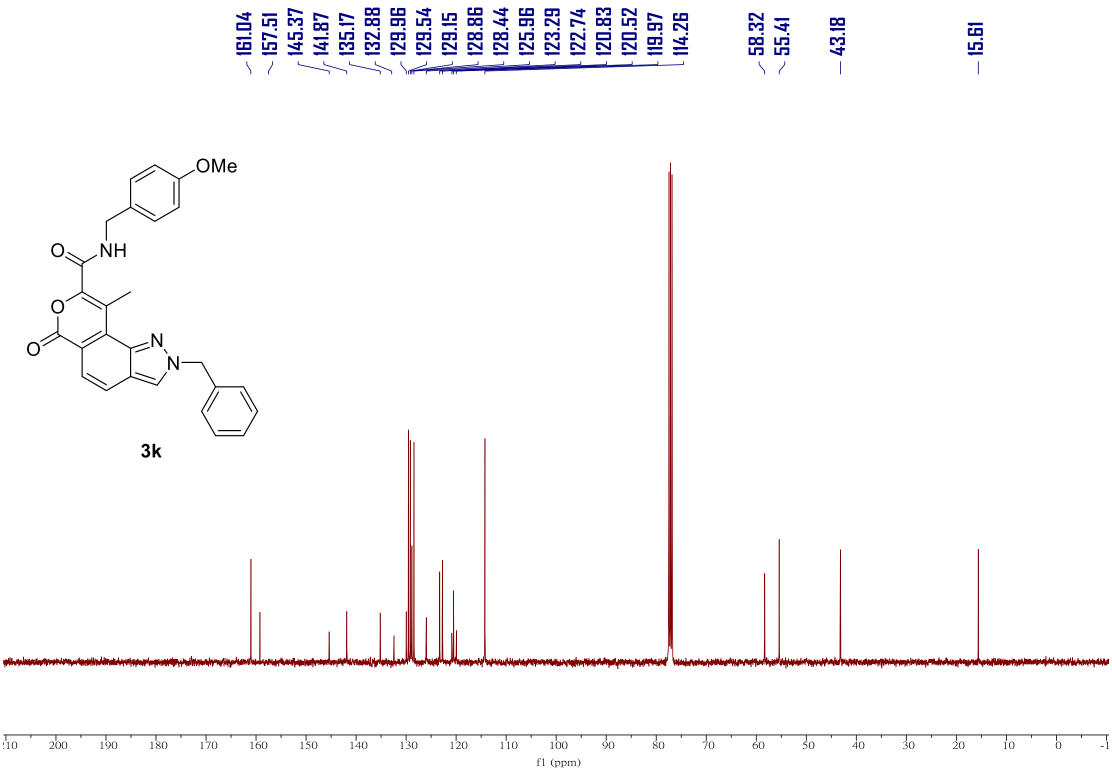

$^{13}C\{^1H\}$ spectrum (400 MHz) of compound **3k** in $CDCl_3$

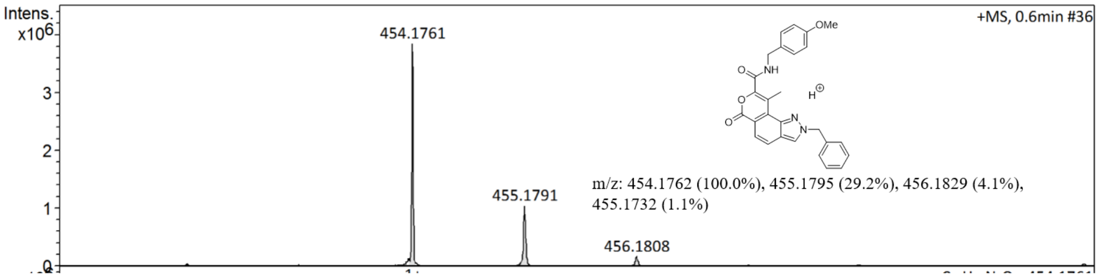

Display Report

Meas. m/z	#	Ion Formula	m/z	err [ppm]	mSigma	# Sigma	Score	rdb	e⁻ Conf	N-Rule	Adduct
454.1761	1	C27H24N3O4	454.1761	-0.0	18.7	1	100.00	17.5	even	ok	M+H

HRMS Mass (ESI) spectrum of compound **3k**

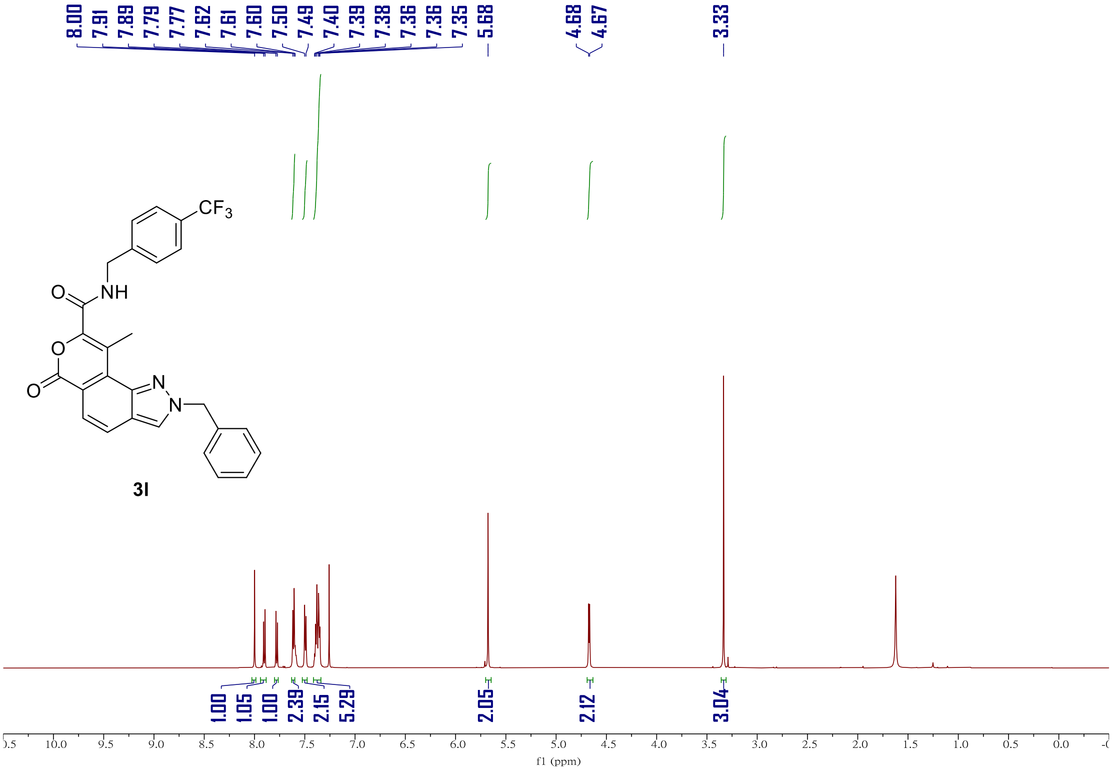

^{1}H spectrum (600 MHz) of compound **3l** in $CDCl_3$

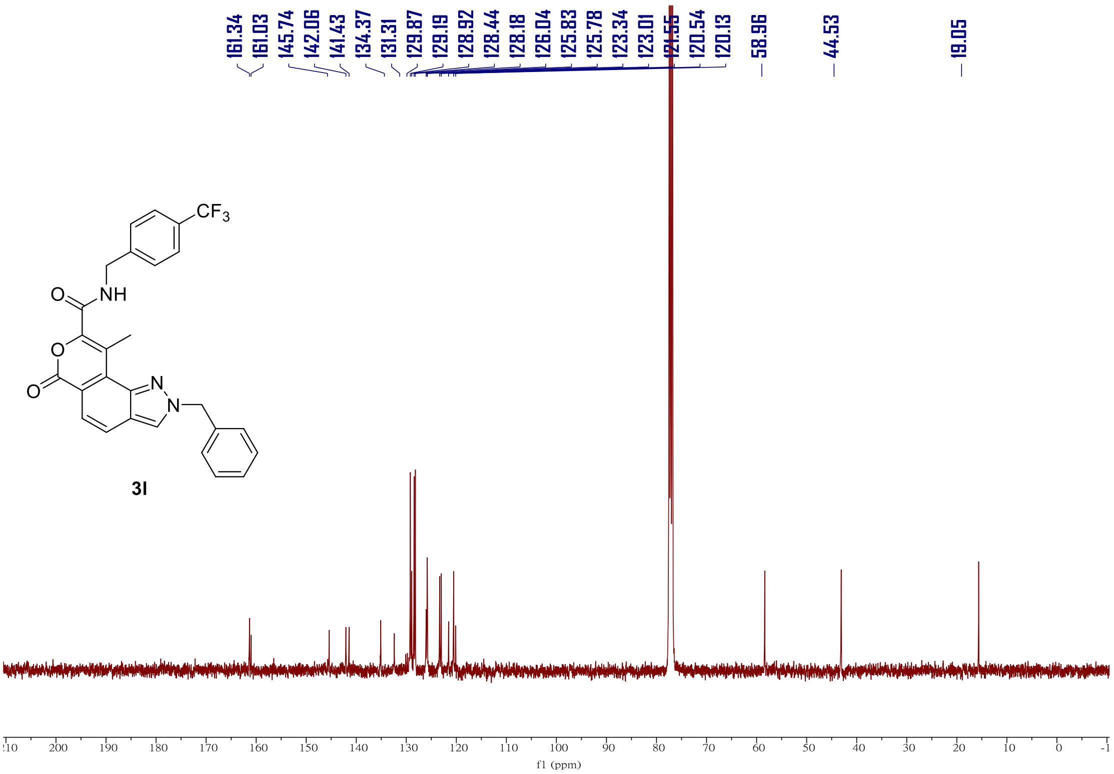

$^{13}C\{^1H\}$ spectrum (101 MHz) of compound **3l** in $CDCl_3$

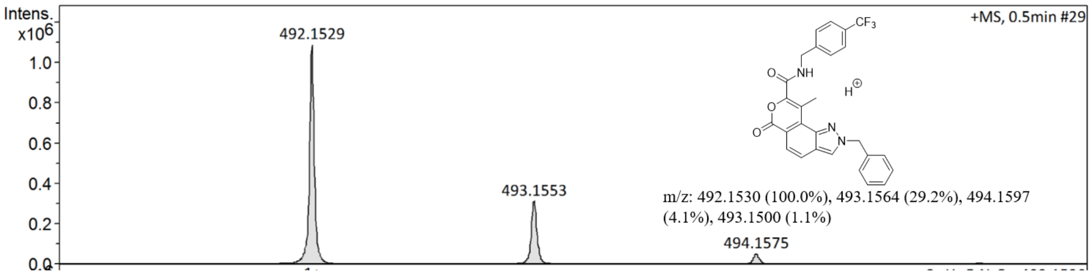

Display Report

Meas. m/z	#	Ion Formula	m/z	err [ppm]	mSigma	# Sigma	Score	rdb	e⁻ Conf	N-Rule	Adduct
492.1529	1	C27H21F3N3O3	492.1530	-0.1	8.8	1	100.00	17.5	even	ok	M+H

HRMS Mass (ESI) spectrum of compound **3l**

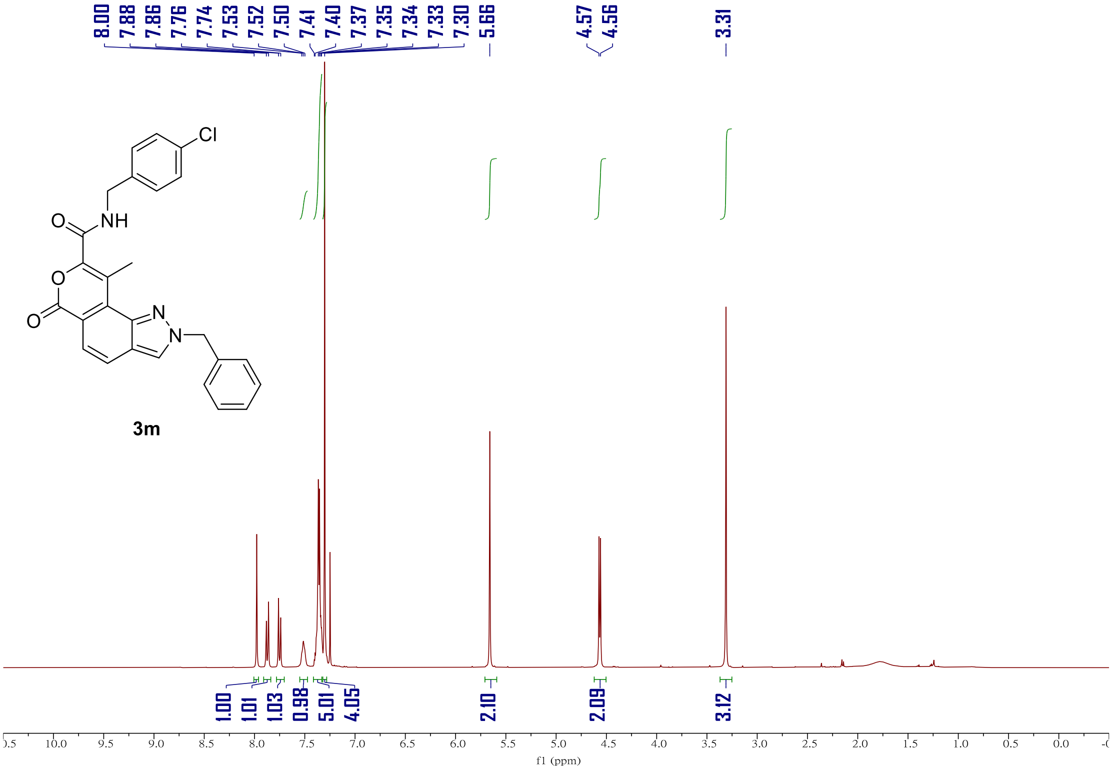

^{1}H spectrum (400 MHz) of compound **3m** in $CDCl_3$

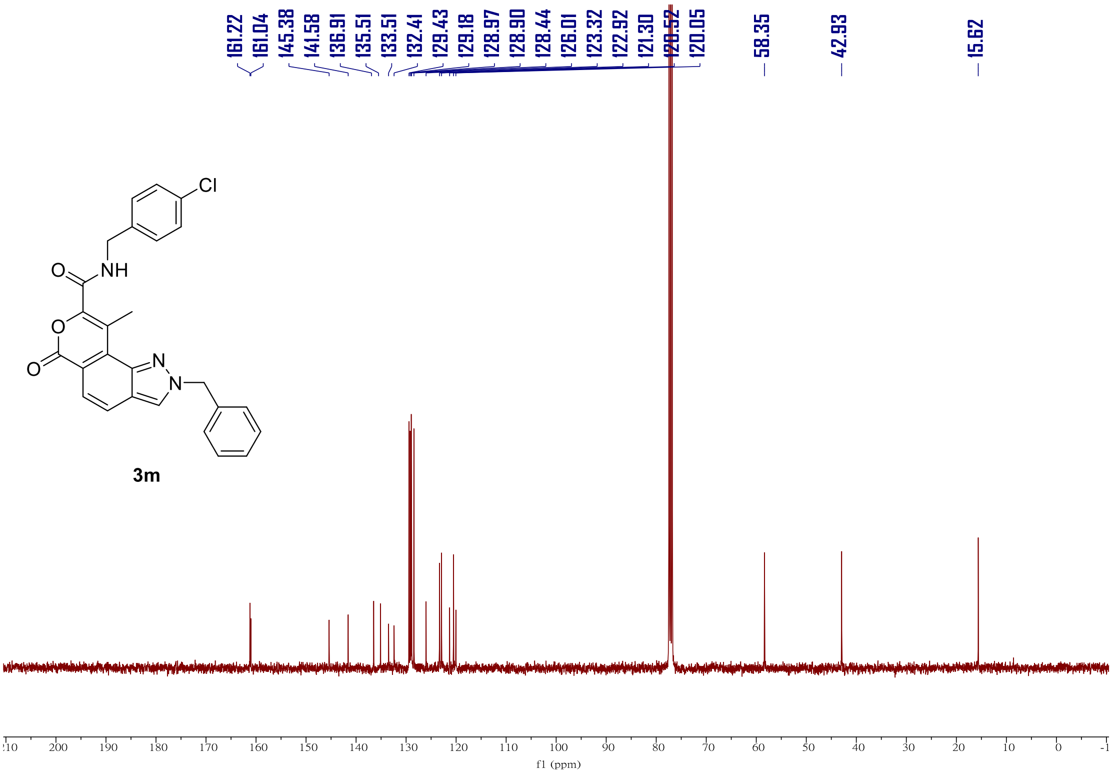

^{13}C{^{1}H} spectrum (101 MHz) of compound **3m** in $CDCl_3$

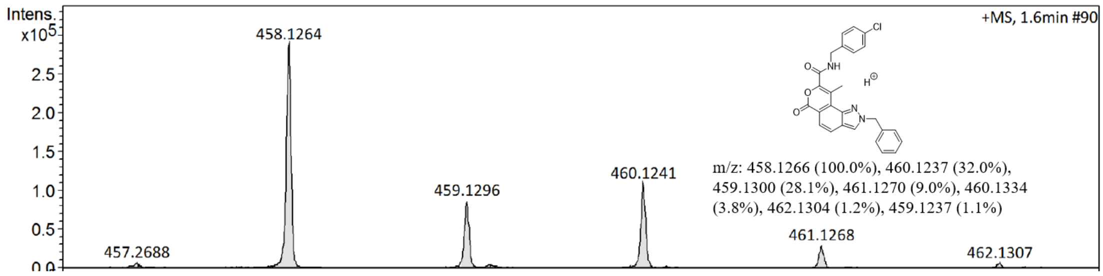

Display Report

Meas. m/z	#	Ion Formula	m/z	err [ppm]	mSigma	# Sigma	Score	rdb	e⁻ Conf	N-Rule	Adduct
458.1264	1	C26H21ClN3O3	458.1266	-0.4	5.5	1	100.00	17.5	even	ok	M+H

HRMS Mass (ESI) spectrum of compound **3m**

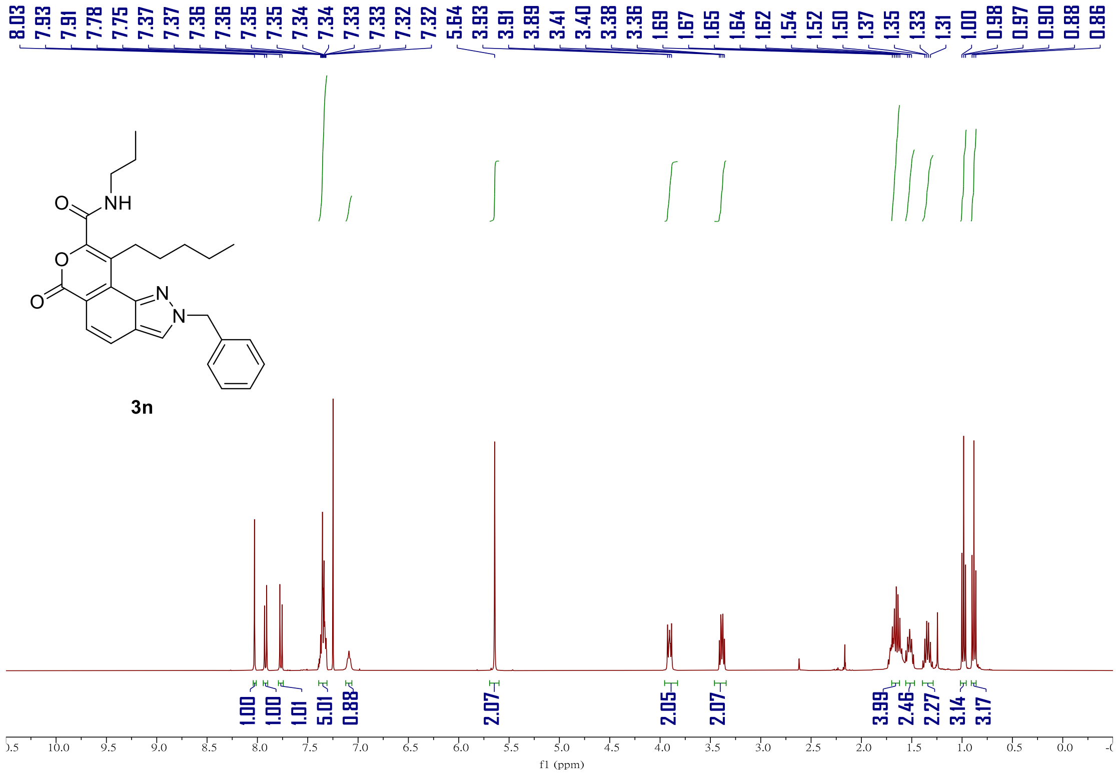

^{1}H spectrum (400 MHz) of compound **3n** in $CDCl_3$

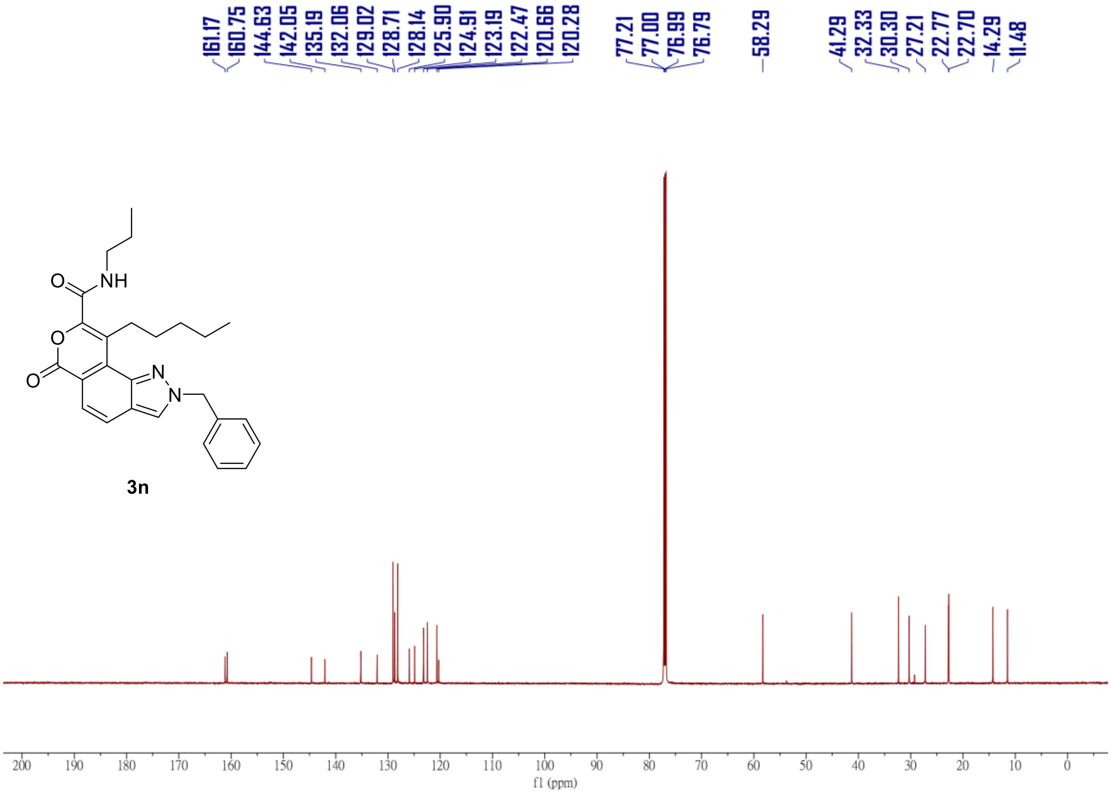

$^{13}C\{^1H\}$ spectrum (151 MHz) of compound **3n** in $CDCl_3$

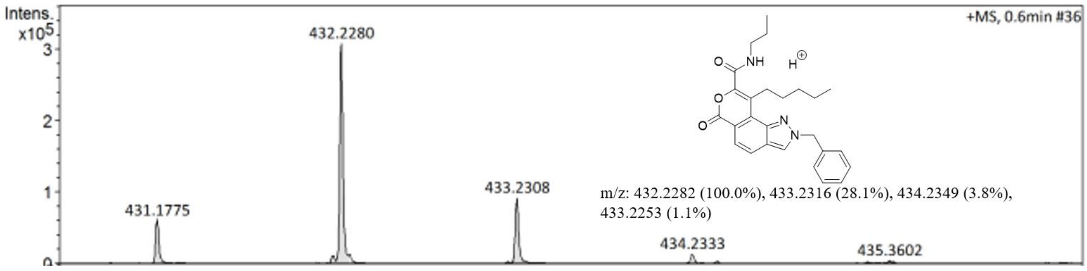

Display Report

Meas. m/z	#	Ion Formula	m/z	err [ppm]	mSigma	# Sigma	Score	rdb	e⁻ Conf	N-Rule	Adduct
432.2280	1	C26H30N3O3	432.2282	-0.4	3.5	1	100.00	13.5	even	ok	M+H

HRMS Mass (ESI) spectrum of compound **3n**

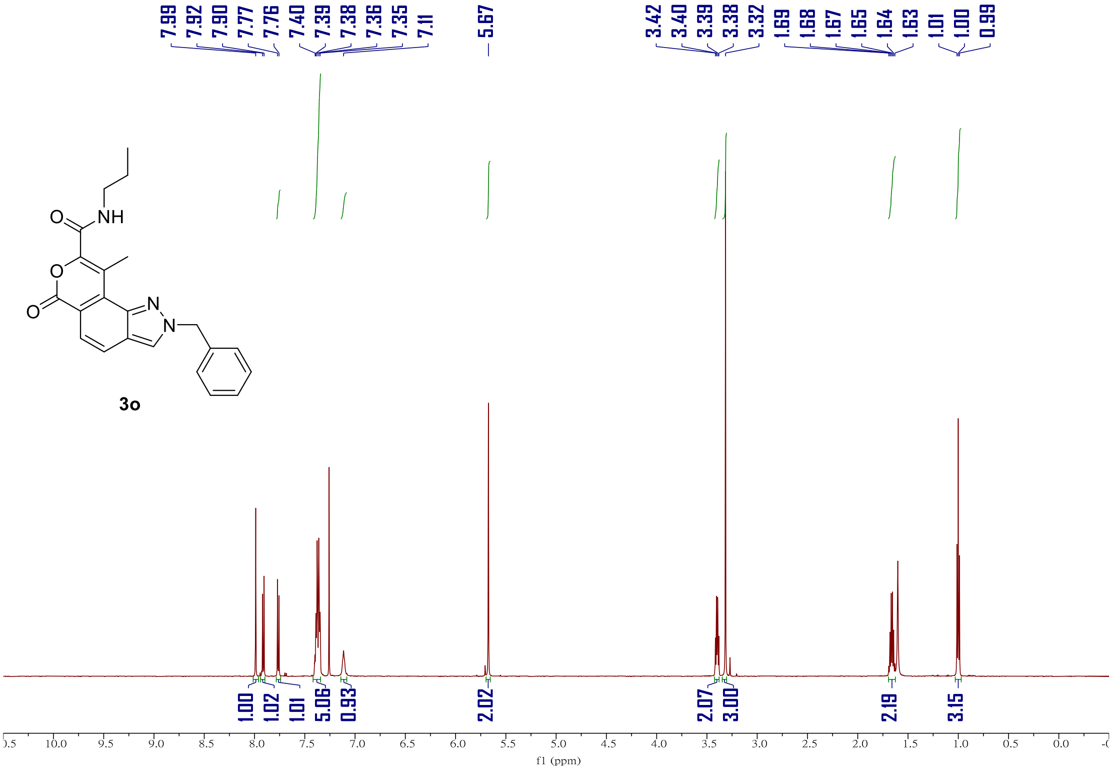

^{1}H spectrum (600 MHz) of compound **3o** in $CDCl_3$

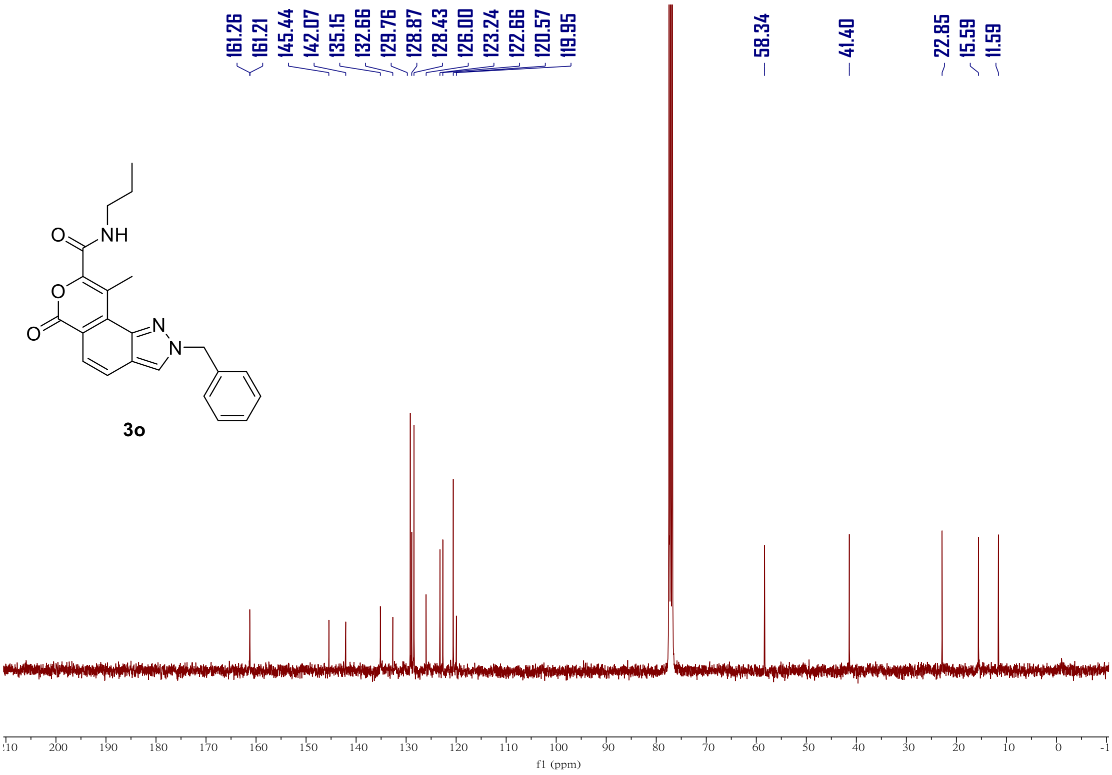

$^{13}C\{^{1}H\}$ spectrum (101 MHz) of compound **3o** in $CDCl_3$

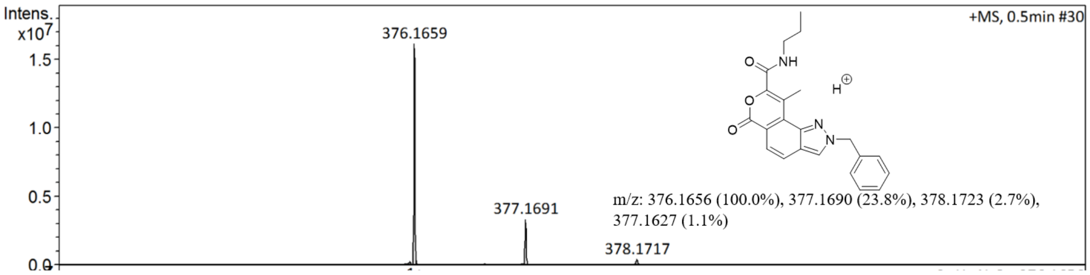

Display Report

Meas. m/z	#	Ion Formula	m/z	err [ppm]	mSigma	# Sigma	Score	rdb	e⁻ Conf	N-Rule	Adduct
376.1659	1	C22H22N3O3	376.1656	0.8	23.4	1	100.00	13.5	even	ok	M+H

HRMS Mass (ESI) spectrum of compound **3o**

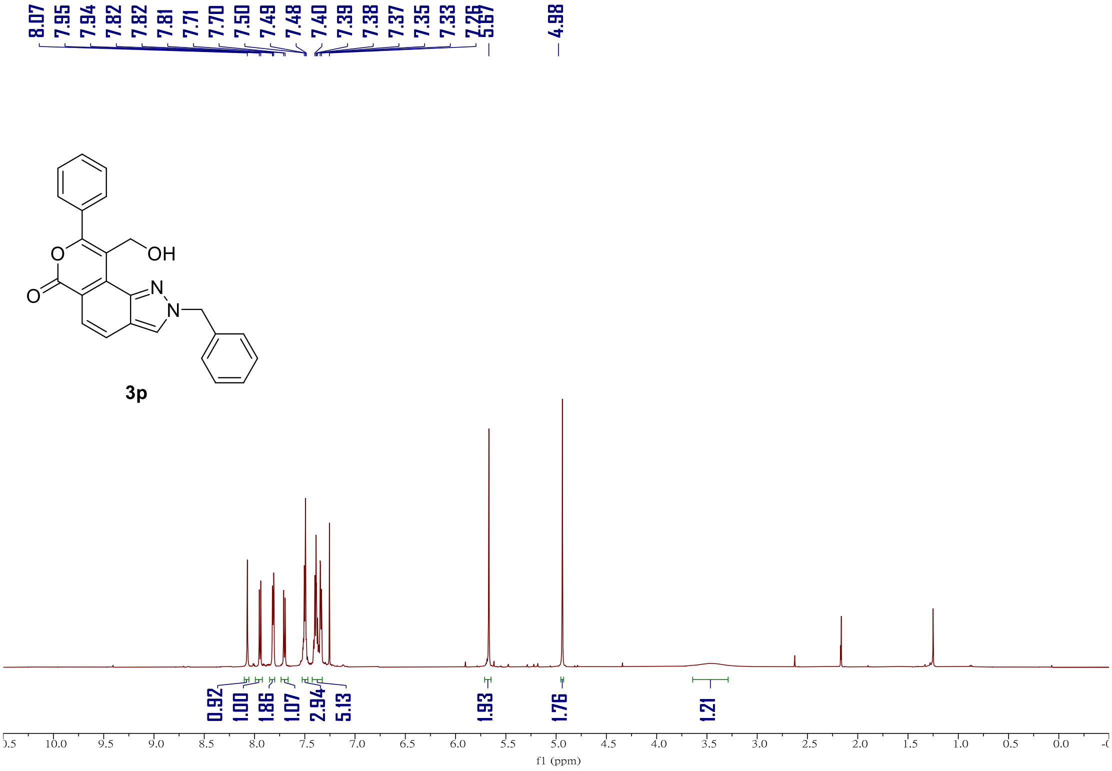

^{1}H spectrum (600 MHz) of compound **3p** in $CDCl_3$

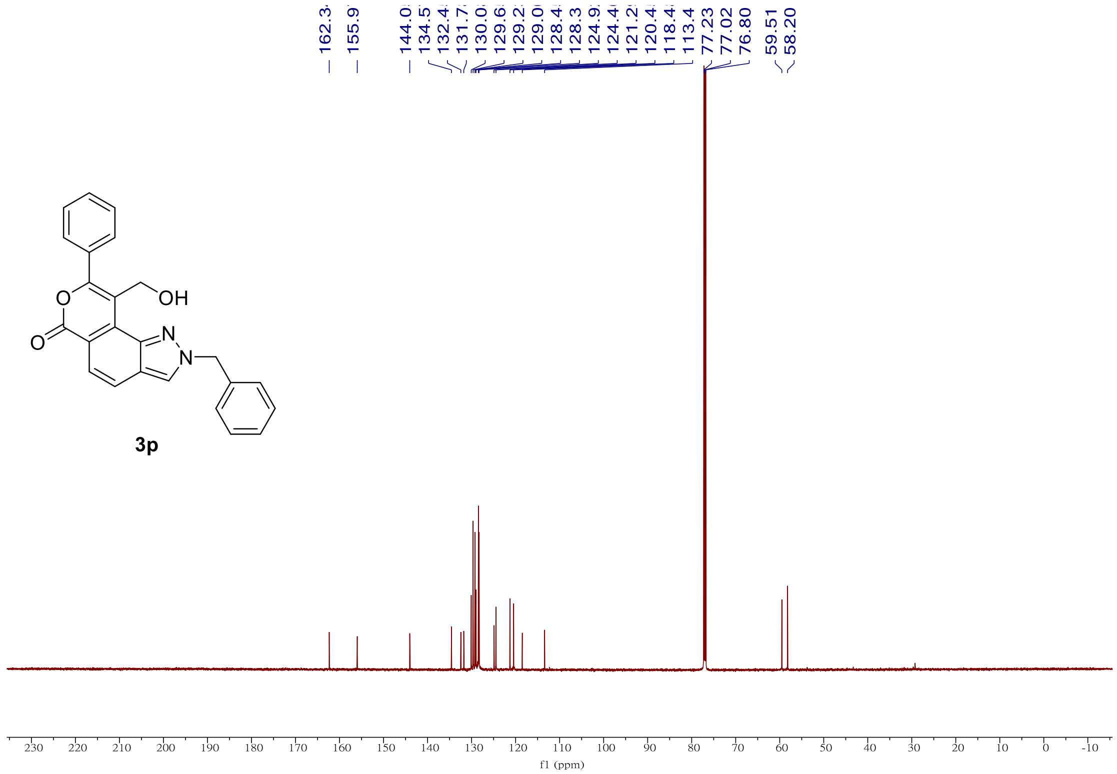

$^{13}C\{^{1}H\}$ spectrum (151 MHz) of compound **3p** in $CDCl_3$

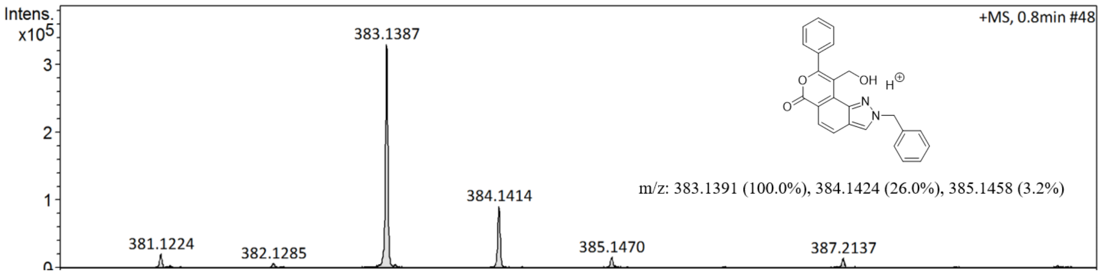

Display Report

Meas. m/z	#	Ion Formula	m/z	err [ppm]	mSigma	# Sigma	Score	rdb	e⁻ Conf	N-Rule	Adduct
383.1387	1	C24H19N2O3	383.1390	0.9	3.7	1	100.00	16.5	even	ok	M+H

HRMS Mass (ESI) spectrum of compound **3p**

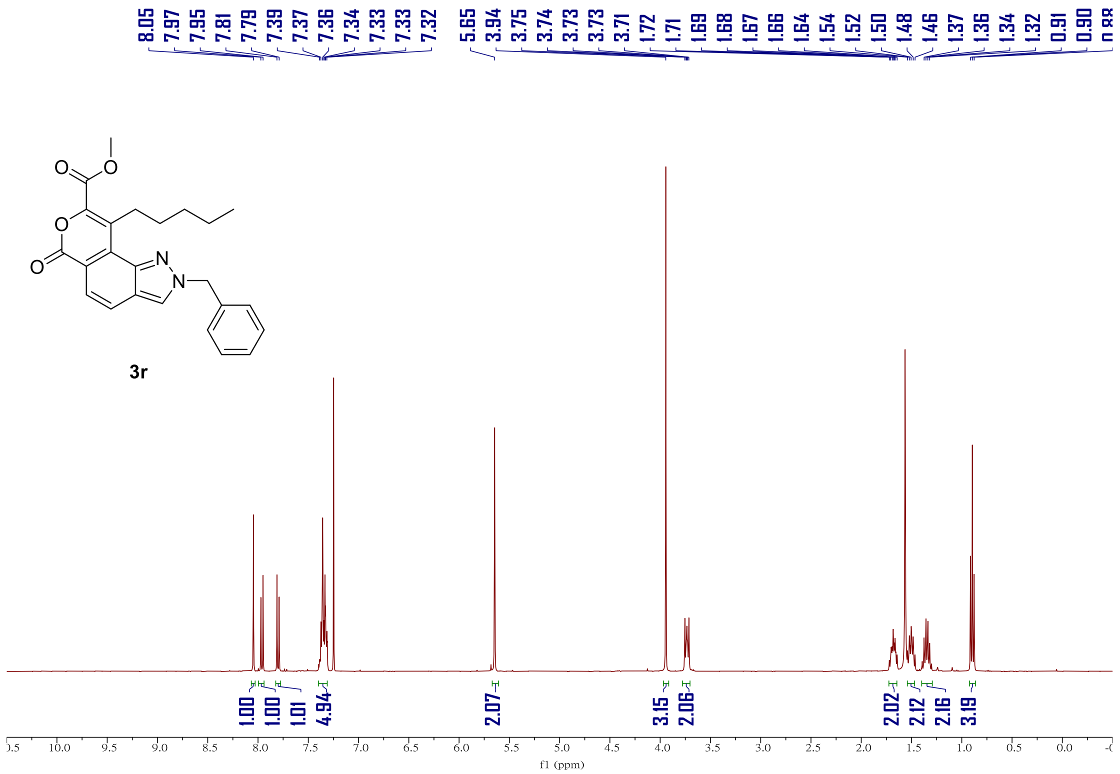

^{1}H spectrum (400 MHz) of compound **3r** in $CDCl_3$

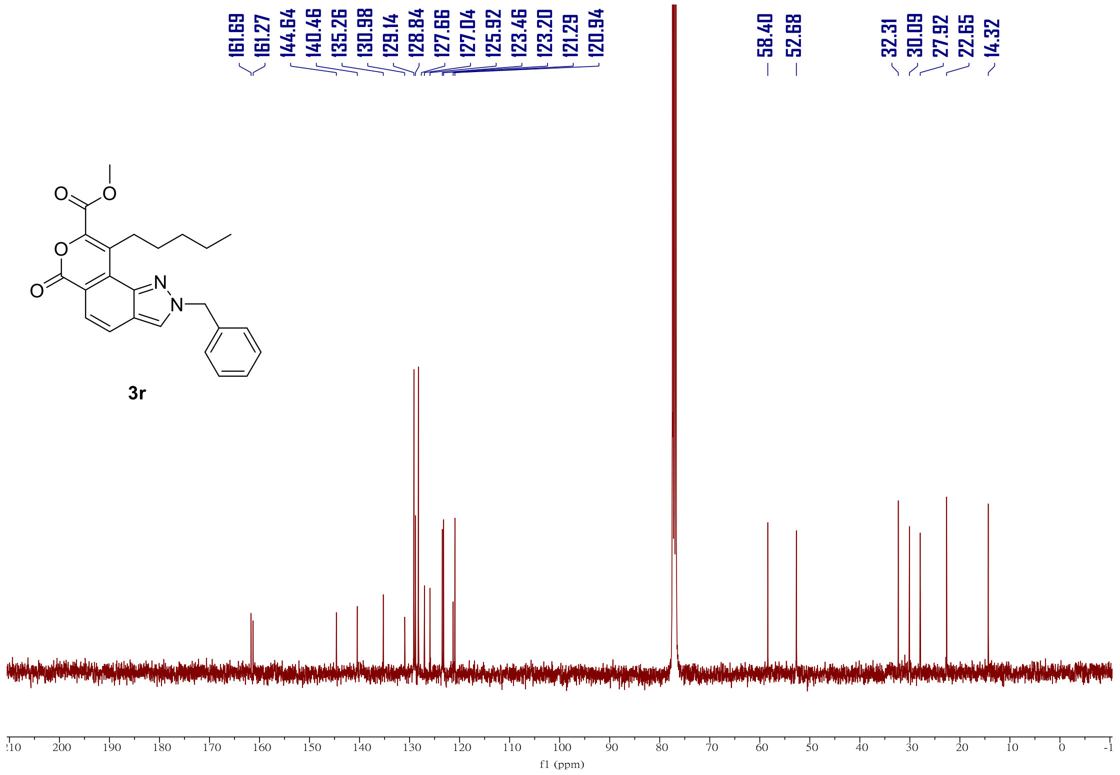

$^{13}C\{^{1}H\}$ spectrum (101 MHz) of compound **3r** in $CDCl_3$

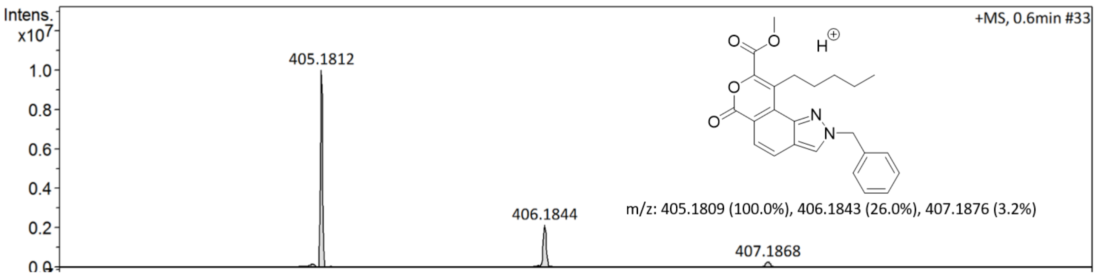

Display Report

Meas. m/z	#	Ion Formula	m/z	err [ppm]	mSigma	# Sigma	Score	rdb	e⁻ Conf	N-Rule	Adduct
405.1812	1	C24H25N2O4	405.1809	0.9	28.3	1	100.00	13.5	even	ok	M+H

HRMS Mass (ESI) spectrum of compound **3r**

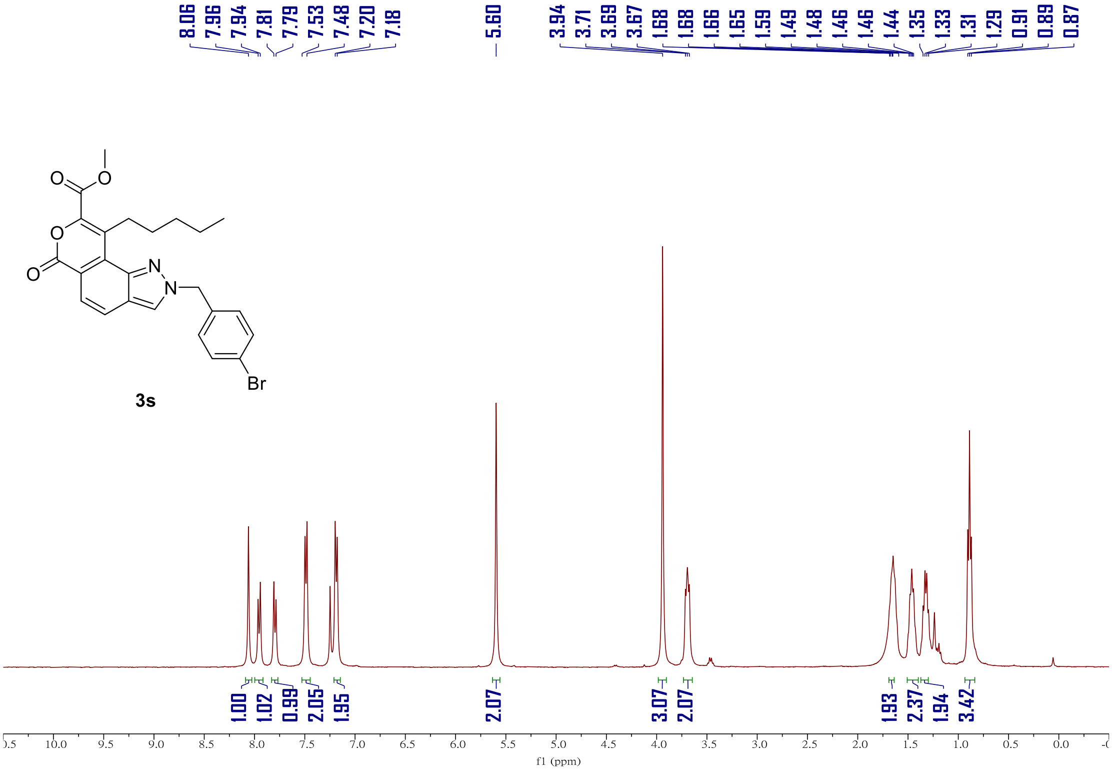

^{1}H spectrum (400 MHz) of compound **3s** in $CDCl_3$

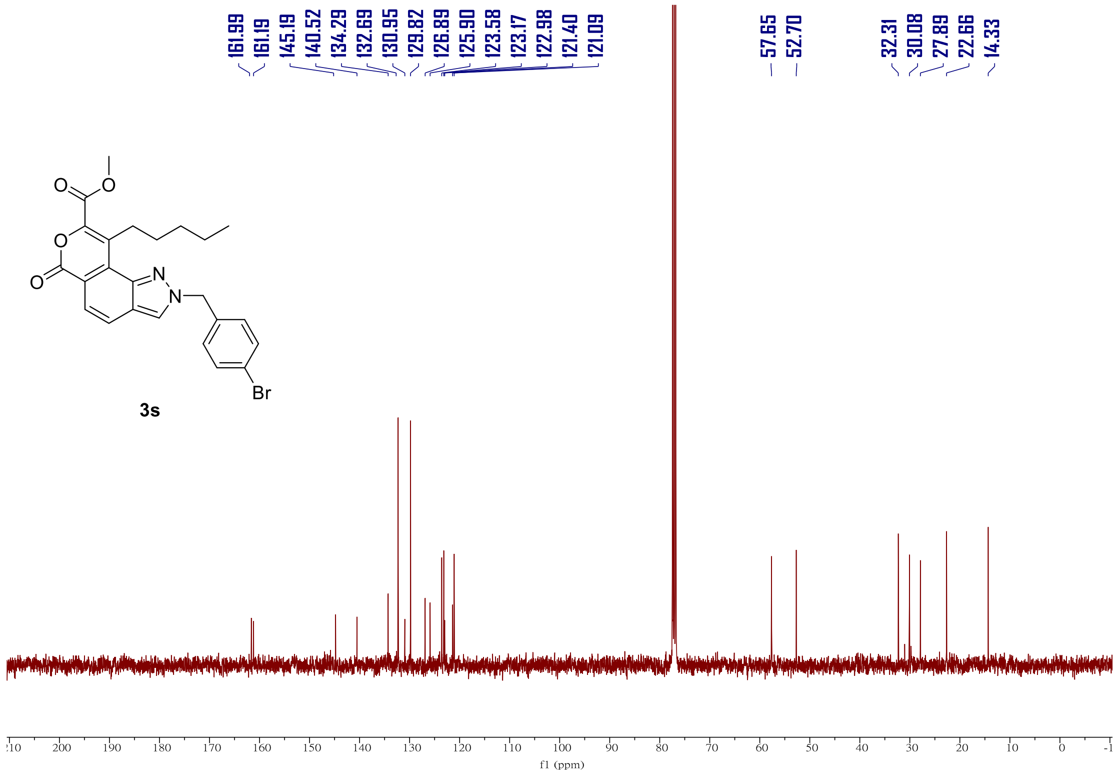

$^{13}C\{^{1}H\}$ spectrum (101 MHz) of compound **3s** in $CDCl_3$

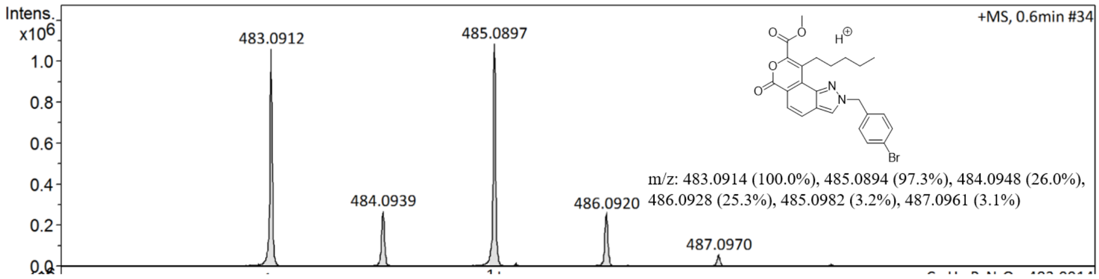

Display Report

Meas. m/z	#	Ion Formula	m/z	err [ppm]	mSigma	# Sigma	Score	rdb	e⁻ Conf	N-Rule	Adduct
483.0912	1	C24H24BrN2O4	483.0914	-0.4	14.8	1	100.00	13.5	even	ok	M+H

HRMS Mass (ESI) spectrum of compound **3s**

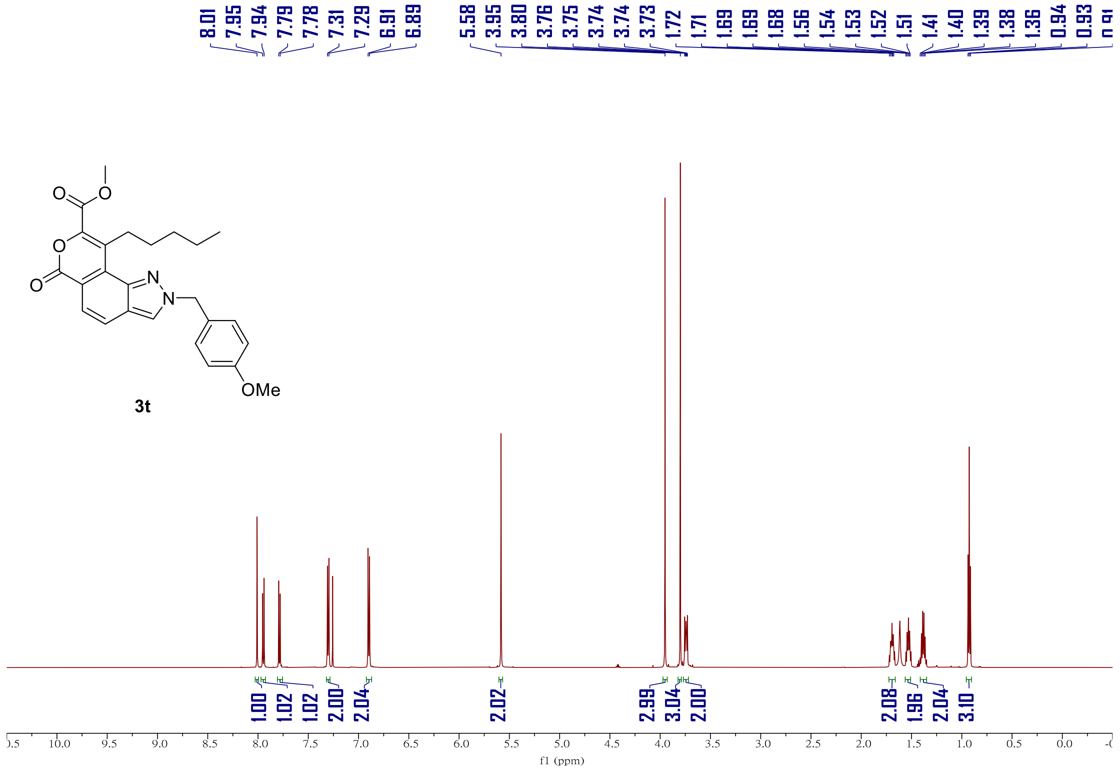

^{1}H spectrum (600 MHz) of compound **3t** in $CDCl_3$

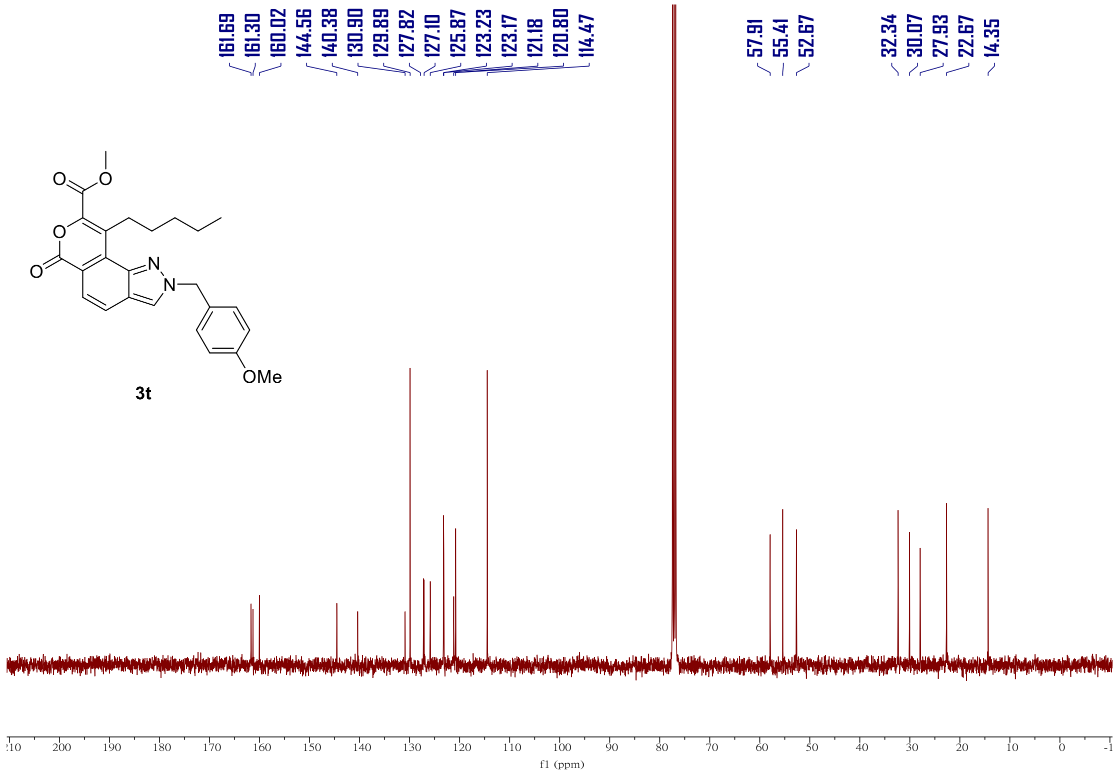

^{13}C{^{1}H} spectrum (101 MHz) of compound **3t** in $CDCl_3$

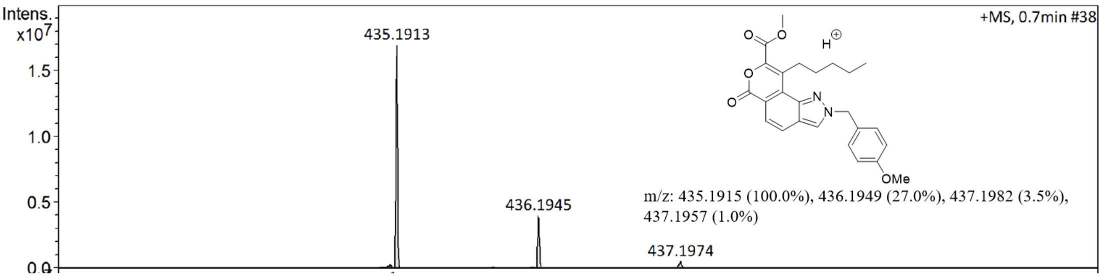

Display Report

Meas. m/z	#	Ion Formula	m/z	err [ppm]	mSigma	# Sigma	Score	rdb	e⁻ Conf	N-Rule	Adduct
435.1913	1	C25H27N2O5	435.1914	-0.4	25.1	1	100.00	13.5	even	ok	M+H

HRMS Mass (ESI) spectrum of compound **3t**

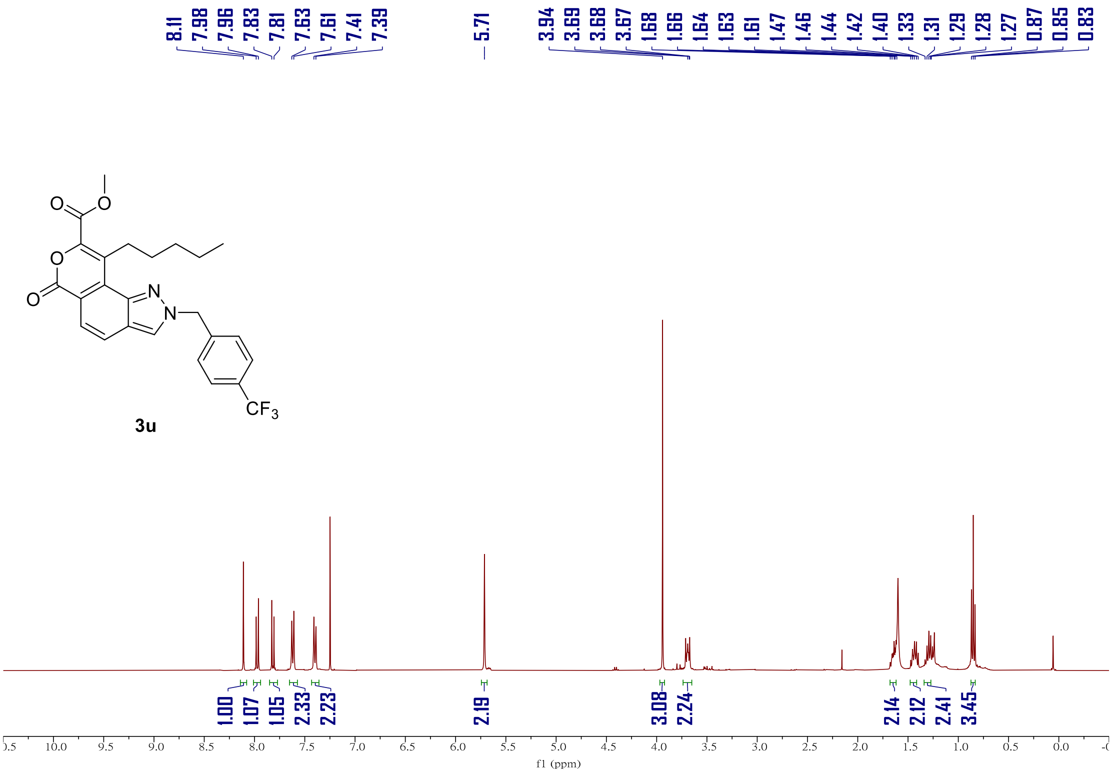

3u

^{1}H spectrum (400 MHz) of compound **3u** in $CDCl_3$

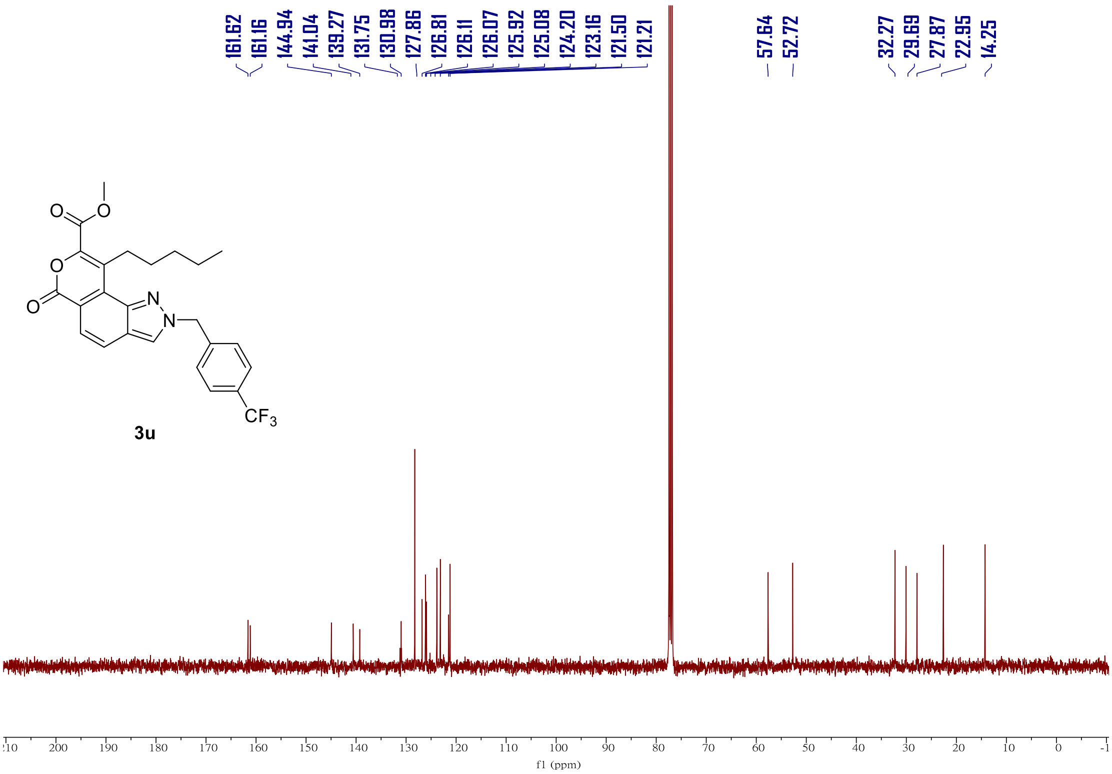

$^{13}C\{^{1}H\}$ spectrum (101 MHz) of compound **3u** in $CDCl_3$

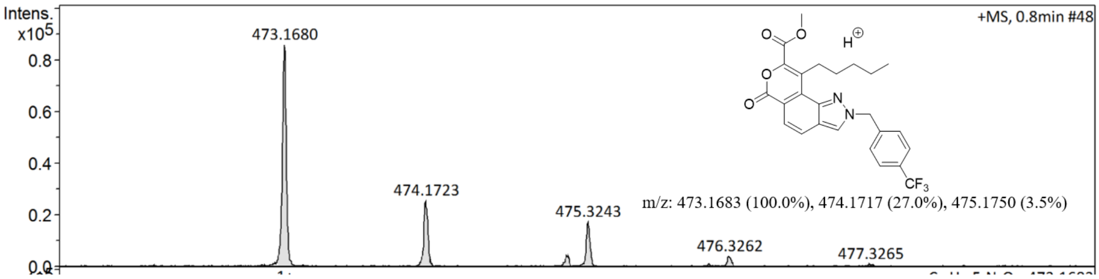

Display Report

Meas. m/z	#	Ion Formula	m/z	err [ppm]	mSigma	# Sigma	Score	rdb	e⁻ Conf	N-Rule	Adduct
473.1680	1	C25H24F3N2O4	473.1683	0.5	9.1	1	100.00	13.5	even	ok	M+H

HRMS Mass (ESI) spectrum of compound **3u**

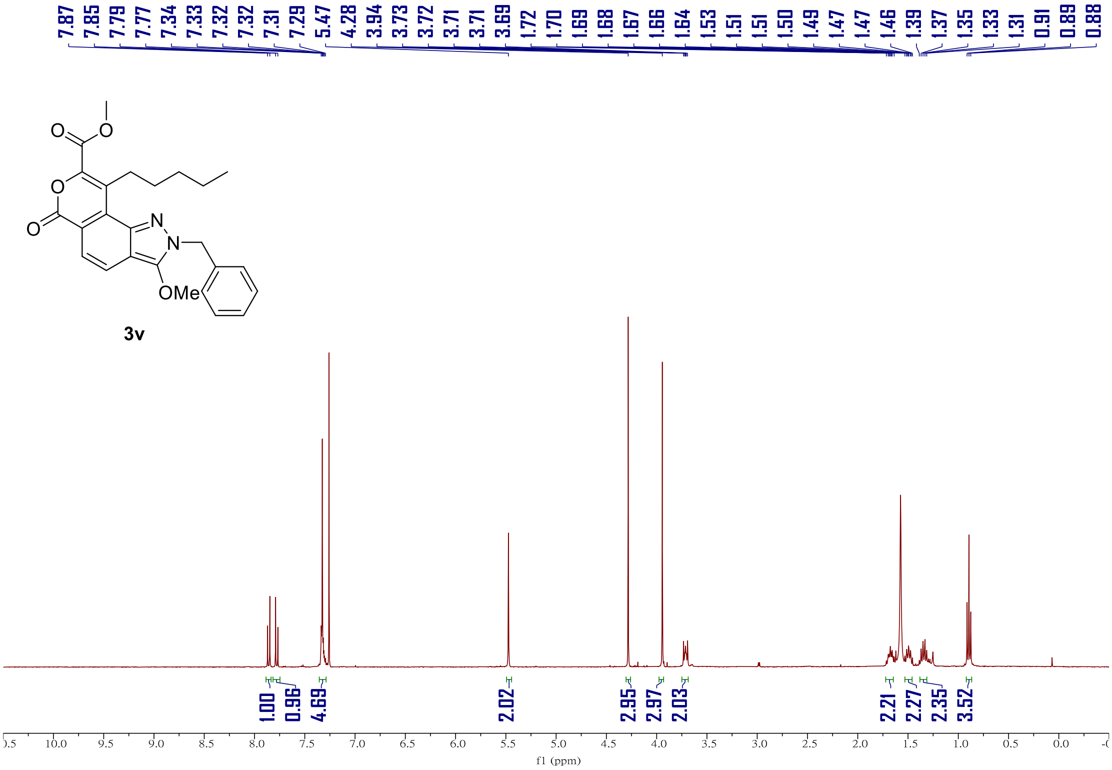

^{1}H spectrum (400 MHz) of compound **3v** in $CDCl_3$

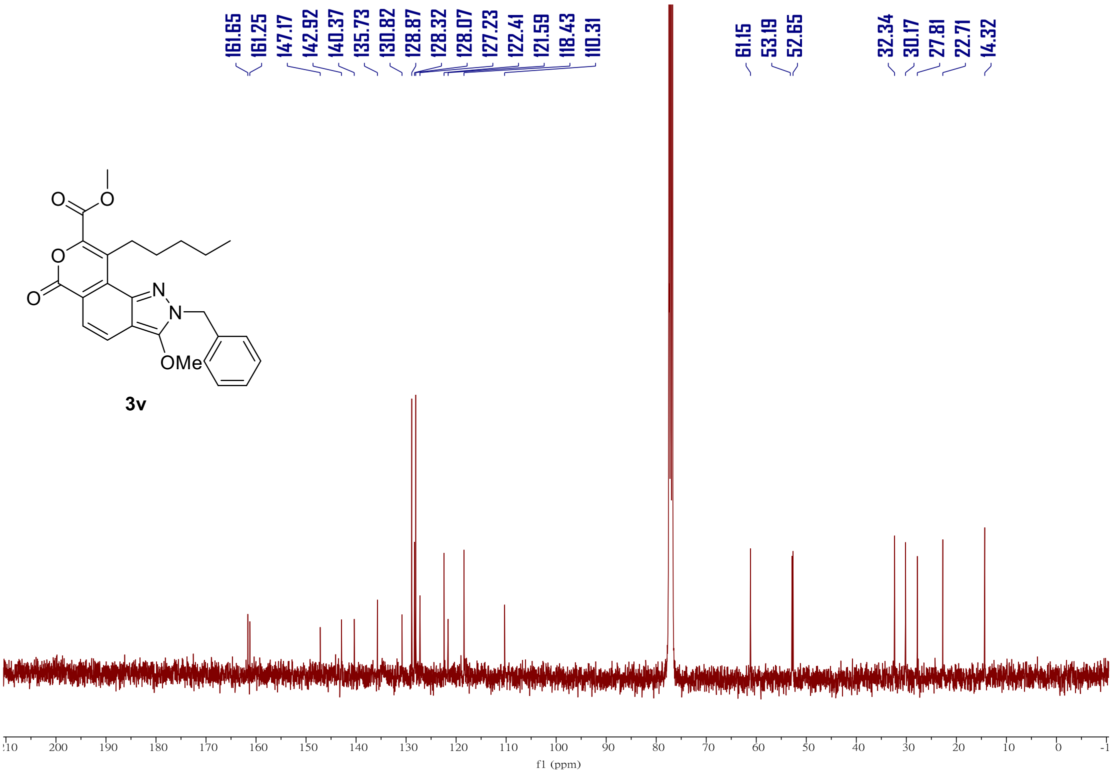

$^{13}C\{^1H\}$ spectrum (101 MHz) of compound **3v** in $CDCl_3$

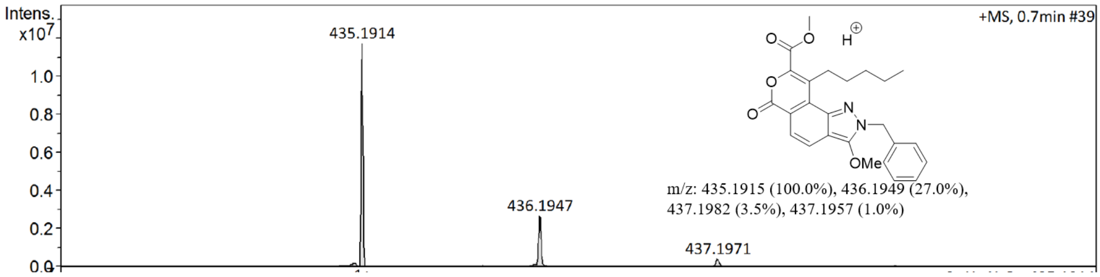

Display Report

Meas. m/z	#	Ion Formula	m/z	err [ppm]	mSigma	# Sigma	Score	rdb	e⁻ Conf	N-Rule	Adduct
435.1914	1	C25H27N2O5	435.1914	-0.2	29.2	1	100.00	13.5	even	ok	M+H

HRMS Mass (ESI) spectrum of compound **3v**

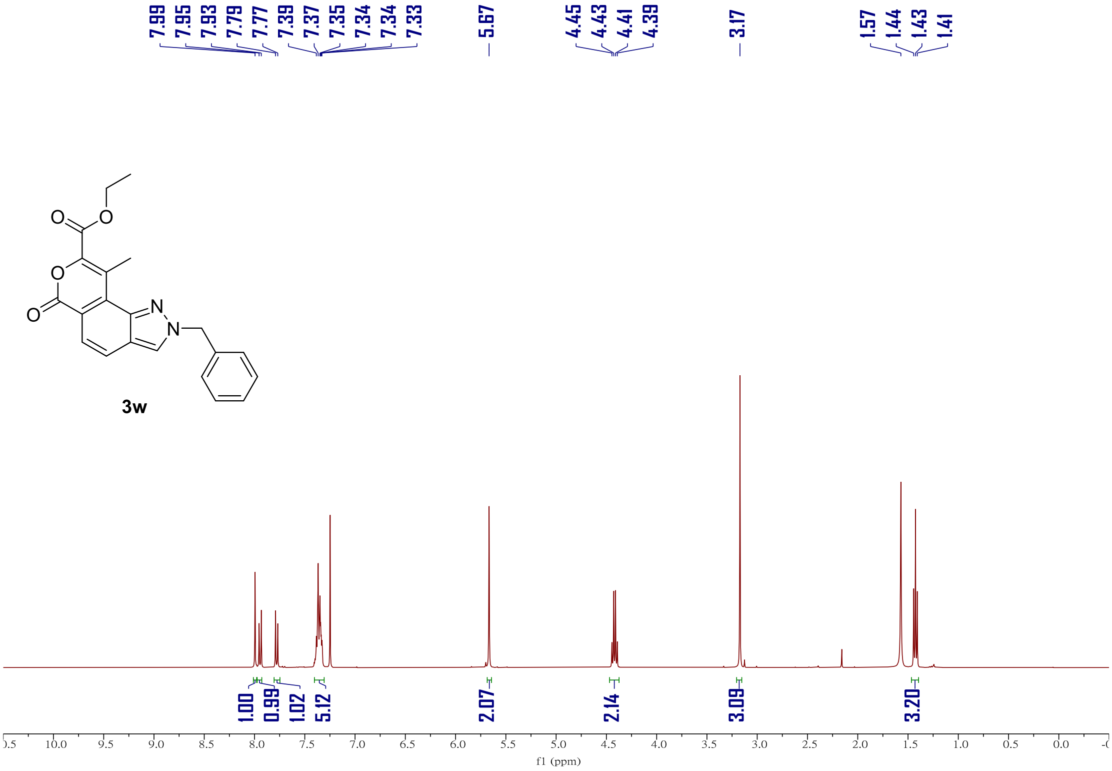

^{1}H spectrum (400 MHz) of compound **3w** in $CDCl_3$

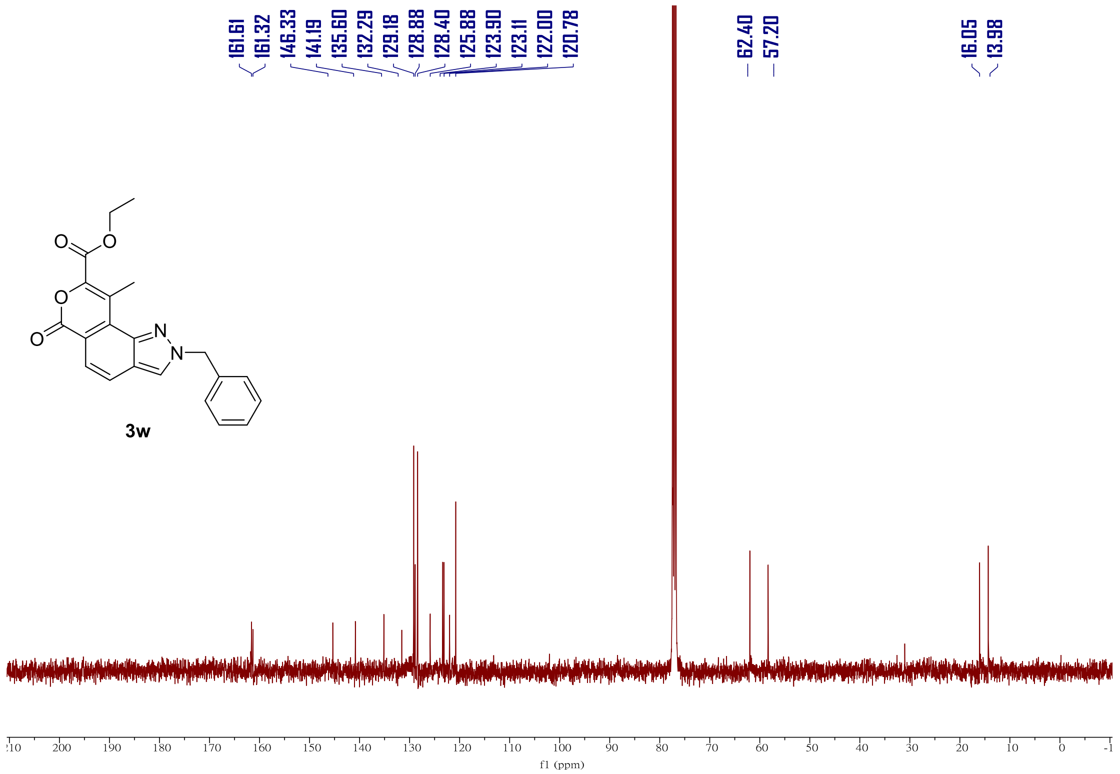

$^{13}C\{^{1}H\}$ spectrum (101 MHz) of compound **3w** in $CDCl_3$

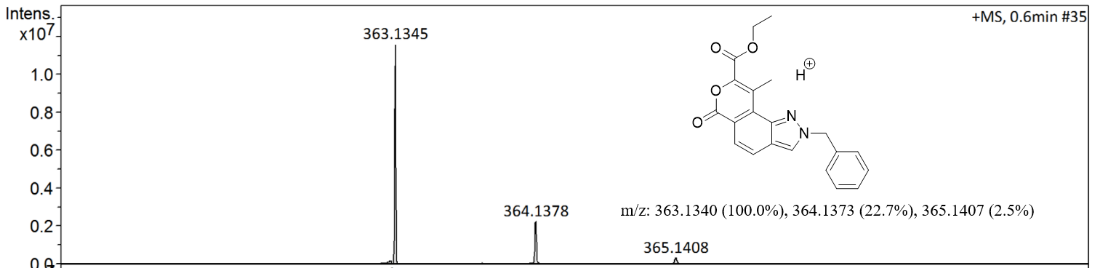

Display Report

Meas. m/z	#	Ion Formula	m/z	err [ppm]	mSigma	# Sigma	Score	rdb	e⁻ Conf	N-Rule	Adduct	Adduct
363.1345	1	C21H19N2O4	363.1339	-1.5	22.4	1	100.00	13.5	even	ok	M+H	M+H

HRMS Mass (ESI) spectrum of compound **3w**

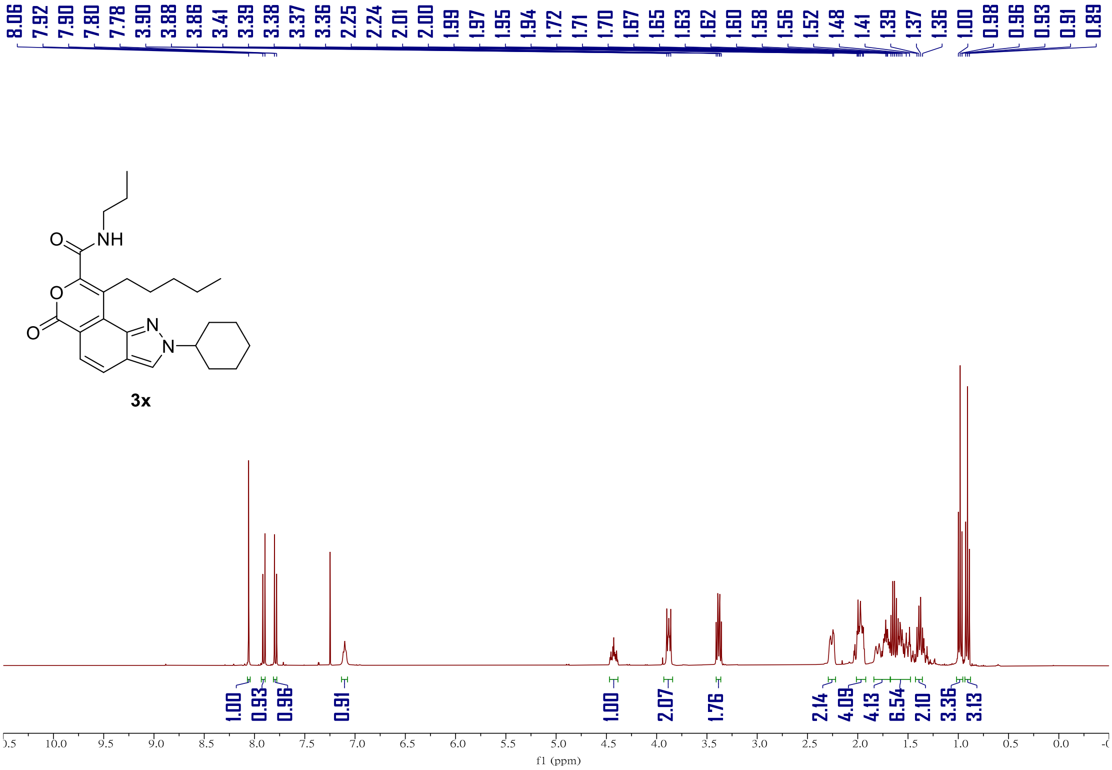

^{1}H spectrum (400 MHz) of compound **3x** in $CDCl_3$

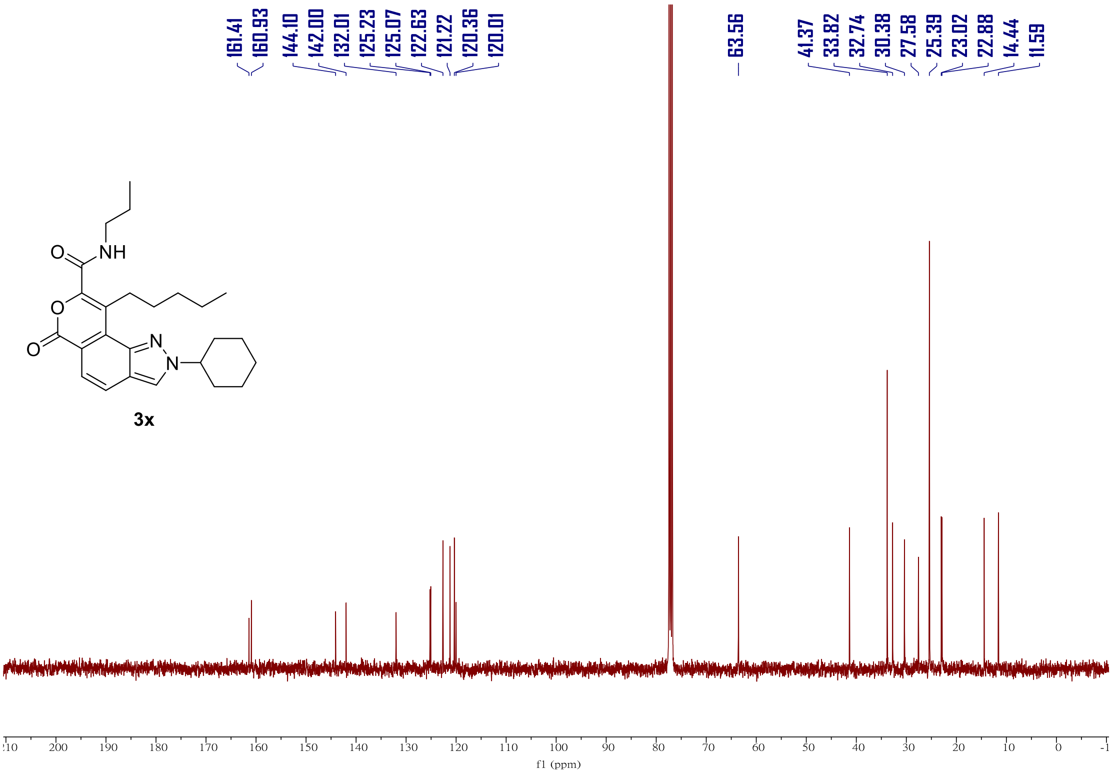

$^{13}C\{^1H\}$ spectrum (101 MHz) of compound **3x** in $CDCl_3$

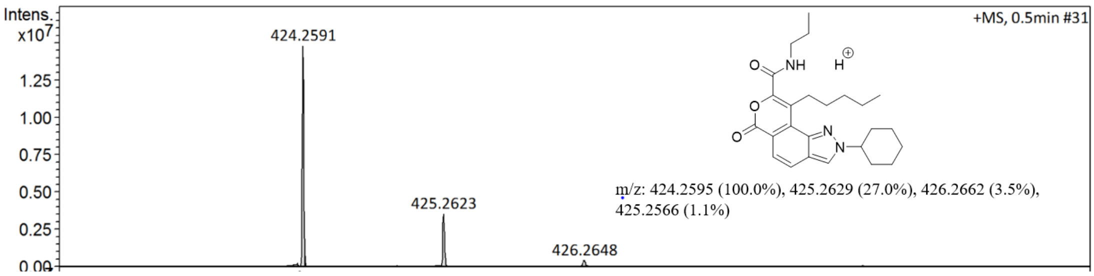

Display Report

Meas. m/z	#	Ion Formula	m/z	err [ppm]	mSigma	# Sigma	Score	rdb	e⁻ Conf	N-Rule	Adduct
424.2591	1	C25H34N3O3	424.2595	-0.8	25.1	1	100.00	10.5	even	ok	M+H

HRMS Mass (ESI) spectrum of compound **3x**

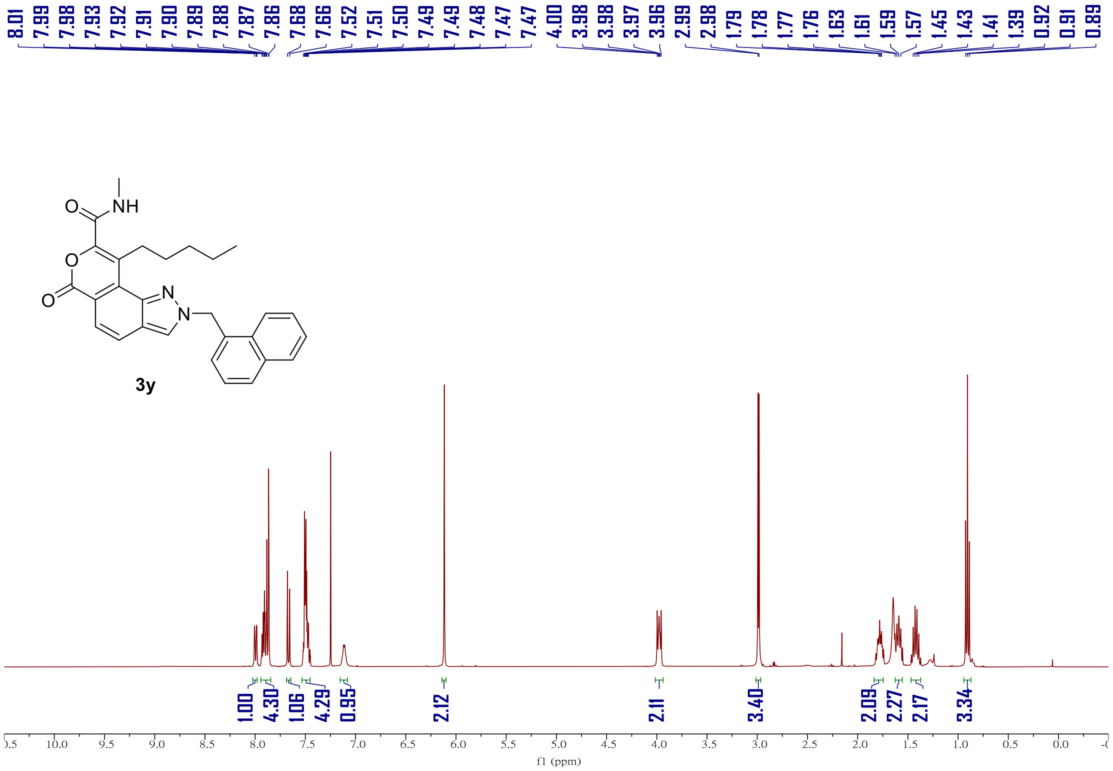

^{1}H spectrum (400 MHz) of compound **3y** in $CDCl_3$

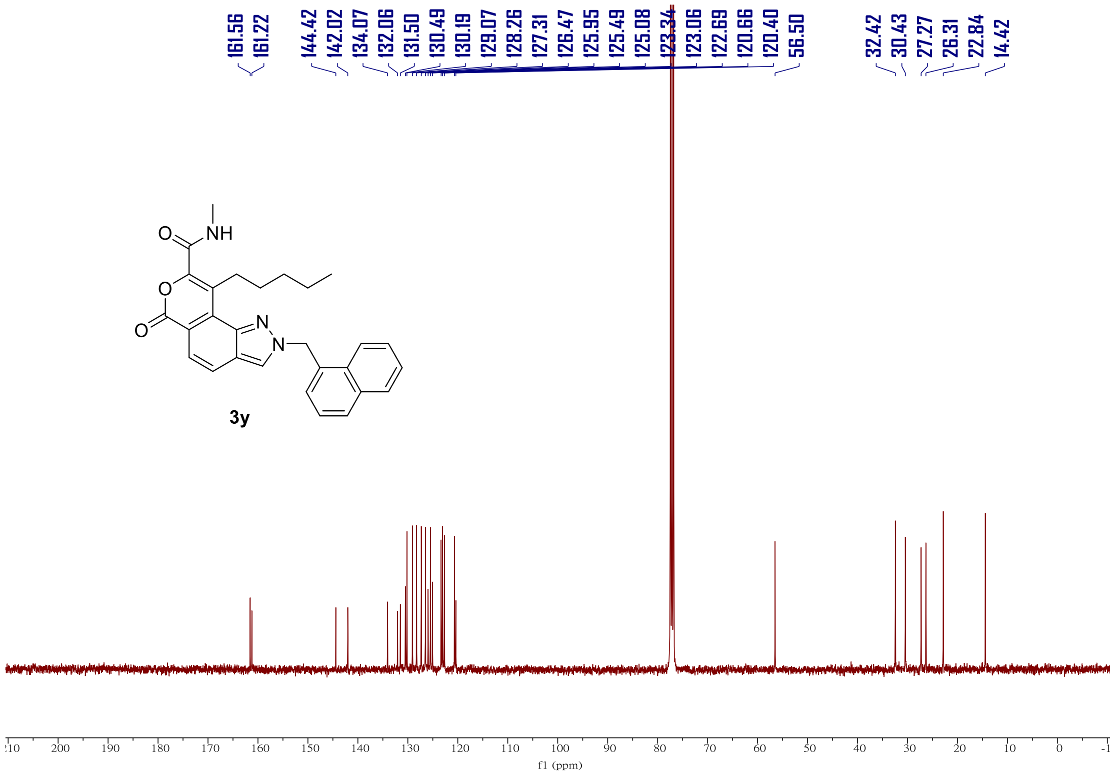

$^{13}C\{^{1}H\}$ spectrum (101 MHz) of compound **3y** in $CDCl_3$

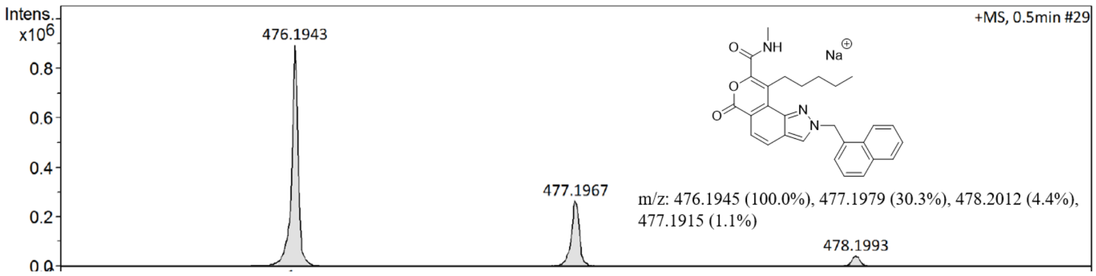

Display Report

Meas. m/z	#	Ion Formula	m/z	err [ppm]	mSigma	# Sigma	Score	rdb	e⁻ Conf	N-Rule	Adduct
476.1943	1	C28H27N3NaO3	476.1945	0.4	11.4	1	100.00	16.5	even	ok	M+Na

HRMS Mass (ESI) spectrum of compound **3y**

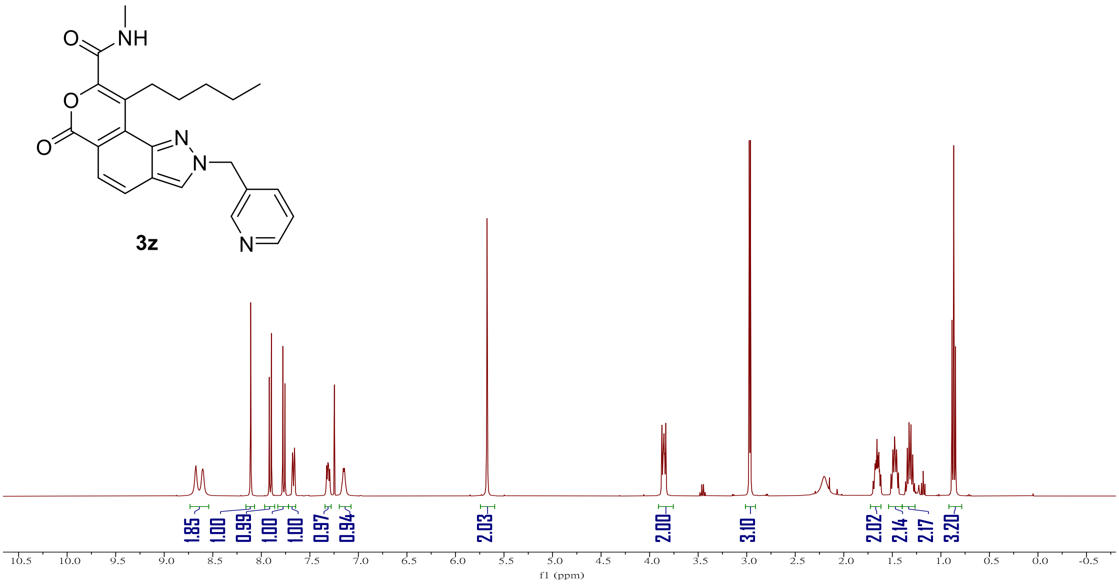

^{1}H spectrum (400 MHz) of compound **3z** in $CDCl_3$

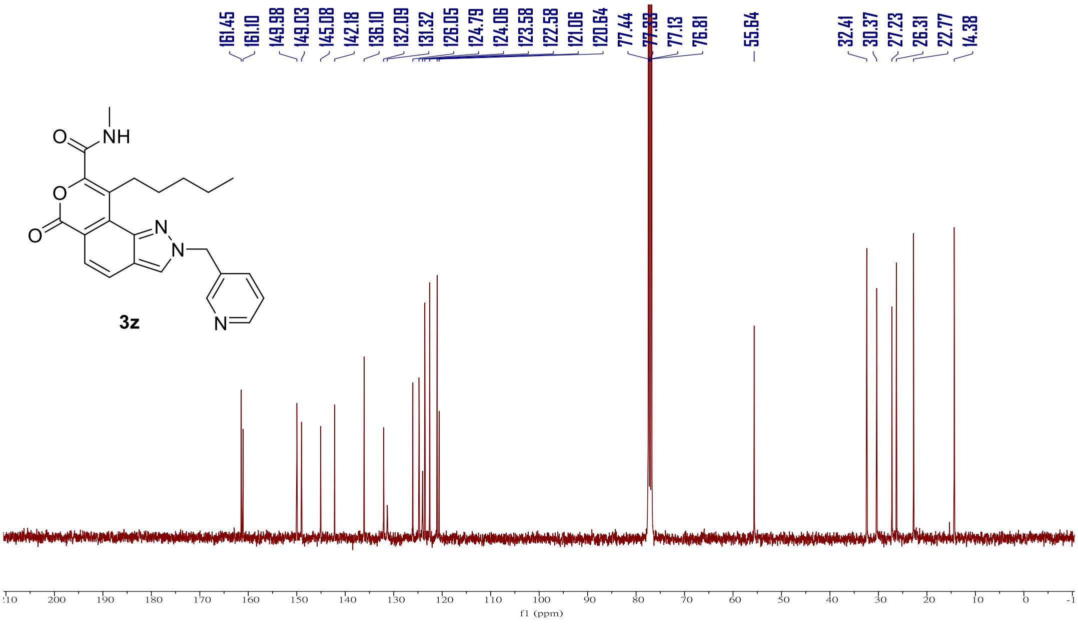

$^{13}C\{^{1}H\}$ spectrum (101 MHz) of compound **3z** in $CDCl_3$

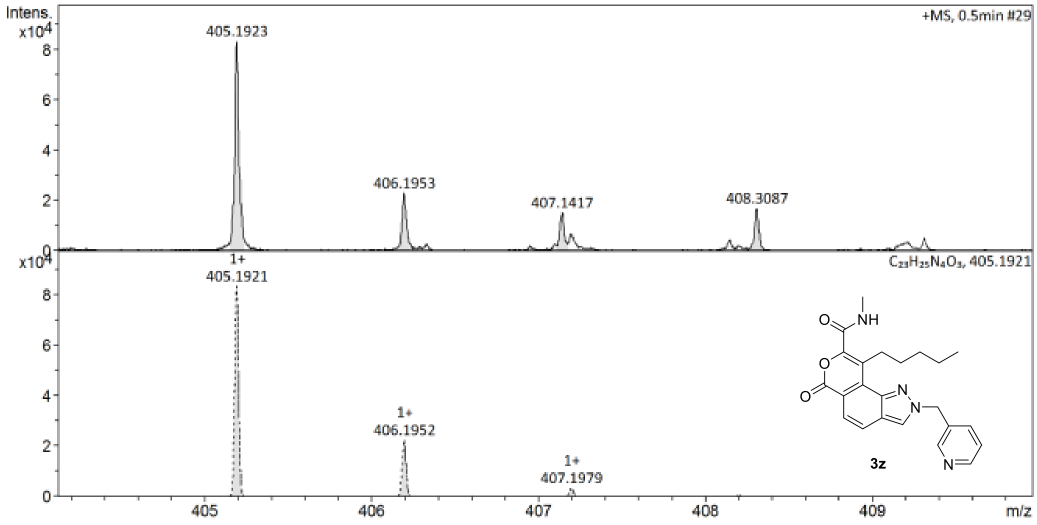

Display Report

Meas. m/z	#	Ion Formula	m/z	err [ppm]	mSigma	# Sigma	Score	rdb	e⁻ Conf	N-Rule	Adduct
405.1923	1	C23H25N4O3	405.1921	0.6	20.7	1	100.00	13.5	even	ok	M+H

HRMS Mass (ESI) spectrum of compound **3z**

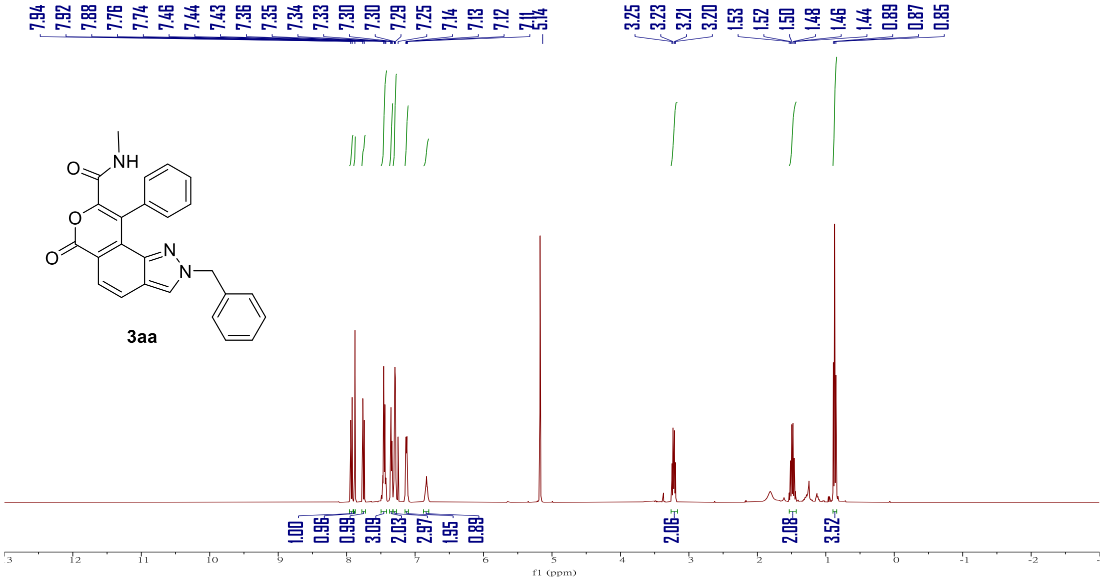

^{1}H spectrum (400 MHz) of compound **3aa** in $CDCl_3$

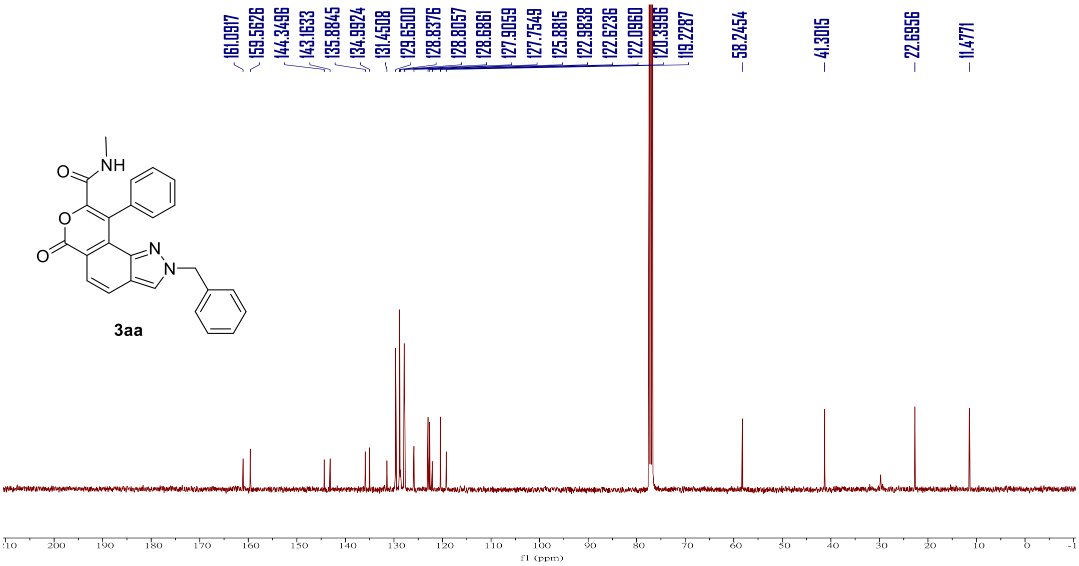

$^{13}C\{^{1}H\}$ spectrum (101 MHz) of compound **3aa** in $CDCl_3$

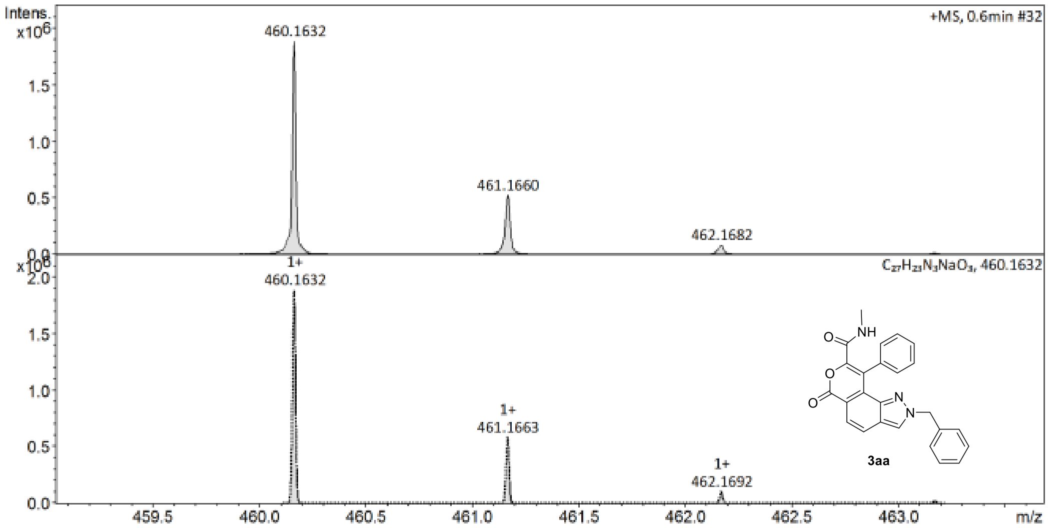

Display Report

Meas. m/z	#	Ion Formula	m/z	err [ppm]	mSigma	# Sigma	Score	rdb	e⁻ Conf	N-Rule	Adduct
460.1632	1	C27H23N3NaO3	460.1632	-0.1	16.2	1	100.00	17.5	even	ok	M+Na

HRMS Mass (ESI) spectrum of compound **3aa**

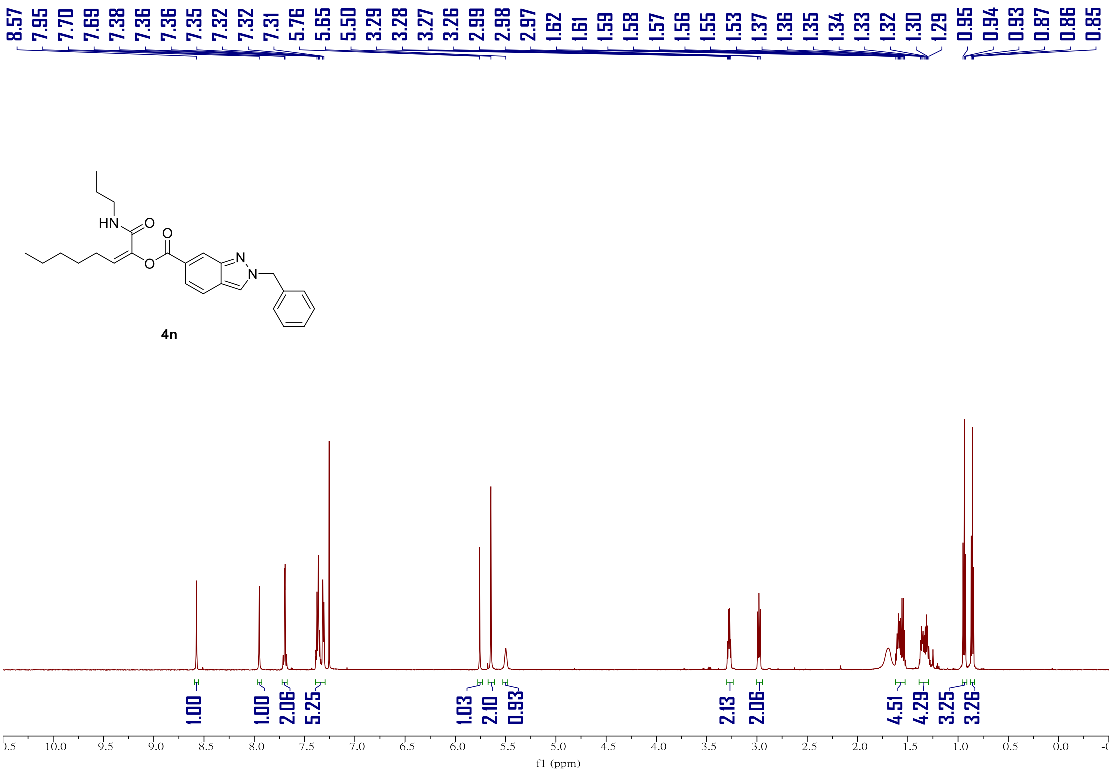

^{1}H spectrum (600 MHz) of compound **4n** in $CDCl_3$

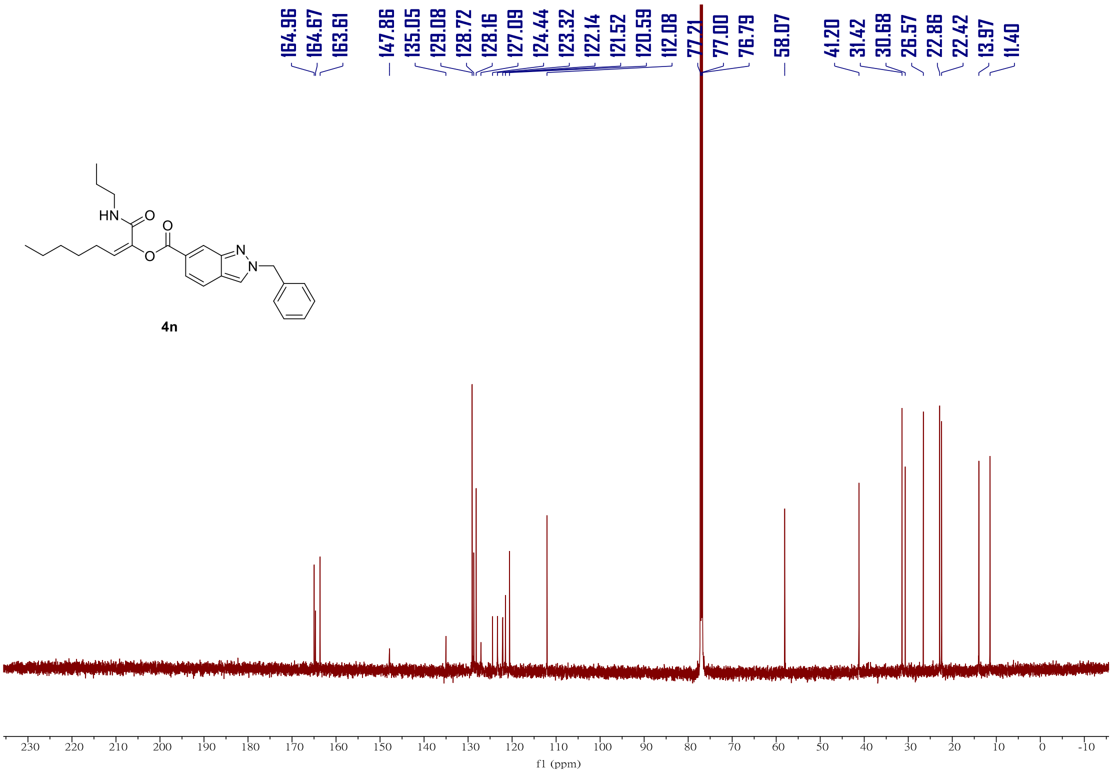

$^{13}C\{^{1}H\}$ spectrum (151 MHz) of compound **4n** in $CDCl_3$

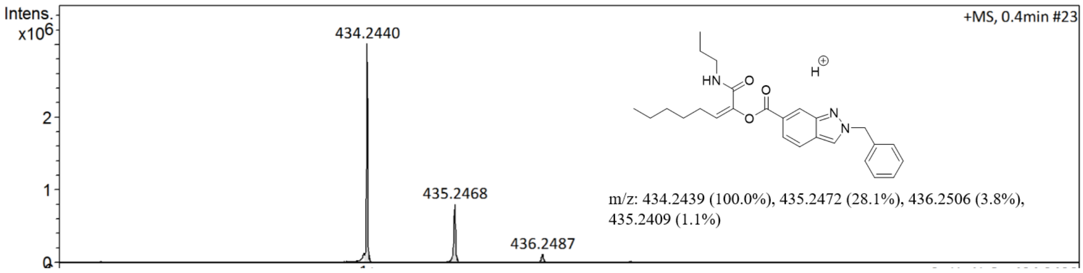

Display Report

Meas. m/z	#	Ion Formula	m/z	err [ppm]	mSigma	# Sigma	Score	rdb	e⁻ Conf	N-Rule	Adduct
434.2440	1	C26H32N3O3	434.2438	0.4	15.3	1	100.00	12.5	even	ok	M+H

HRMS Mass (ESI) spectrum of compound **4n**

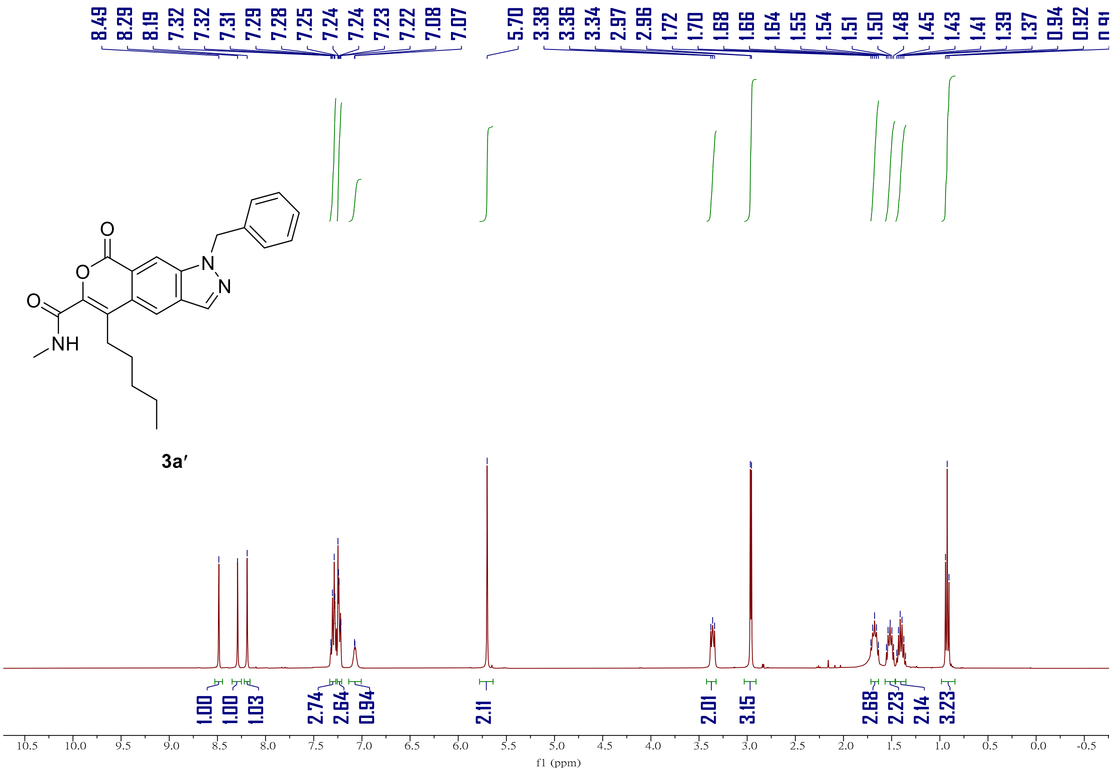

^{1}H spectrum (400 MHz) of compound **3a'** in $CDCl_3$

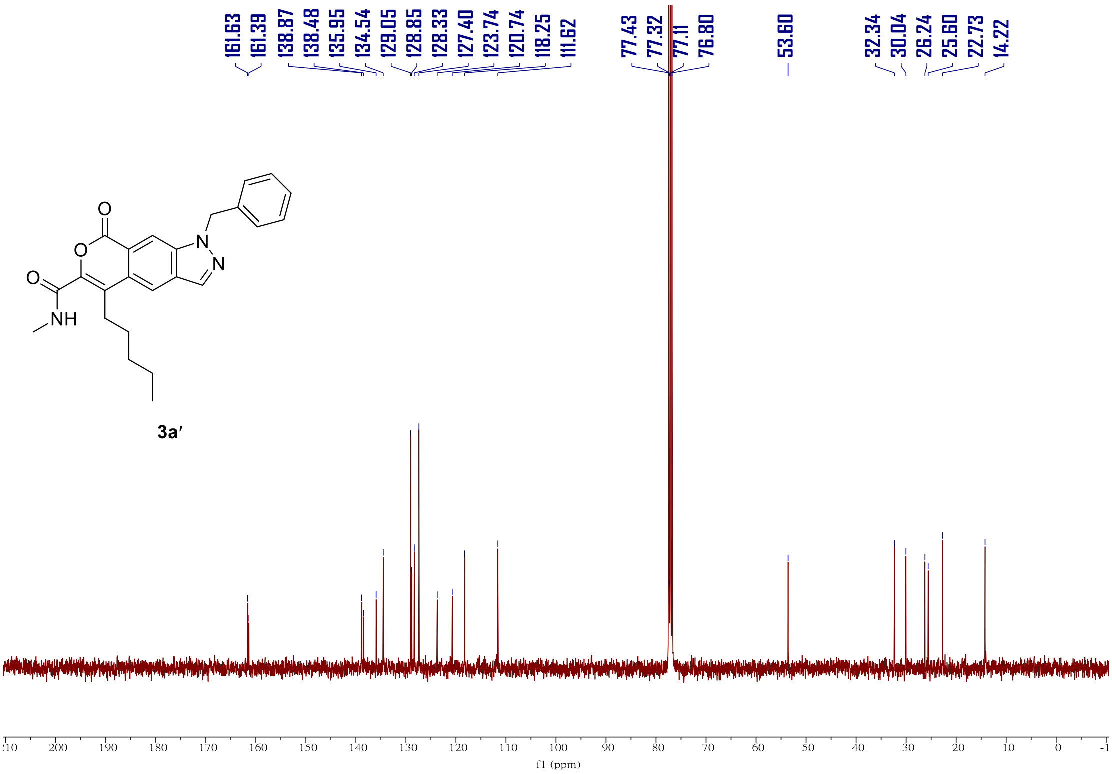

$^{13}C\{^1H\}$ spectrum (101 MHz) of compound **3a'** in $CDCl_3$

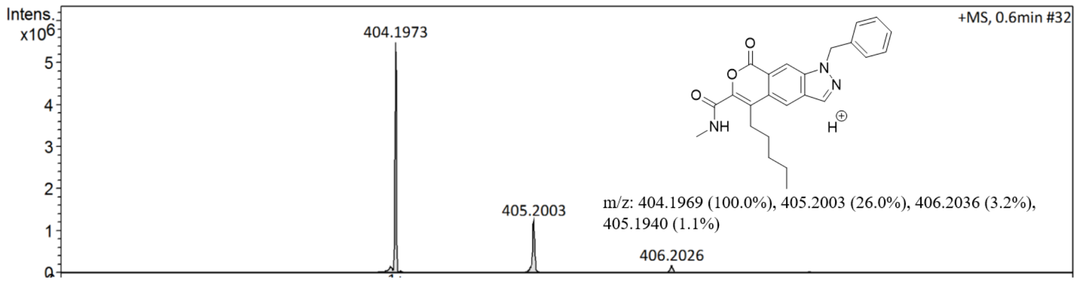

Display Report

Meas. m/z	#	Ion Formula	m/z	err [ppm]	mSigma	# Sigma	Score	rdb	e⁻ Conf	N-Rule	Adduct
404.1973	1	C24H26N3O3	404.1969	1.1	25.4	1	100.00	13.5	even	ok	M+H

HRMS Mass (ESI) spectrum of compound **3a'**

X-ray single crystallographic data of the compounds 3o

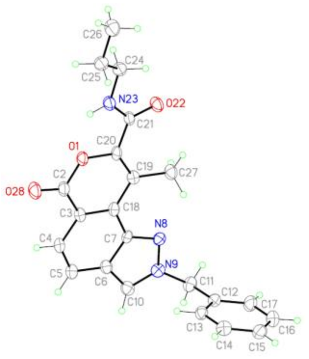

CCDC 2430337

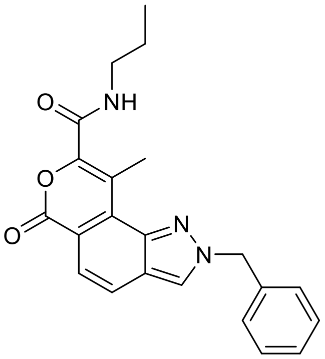

3o

ORTEP diagram of compound 3o. Atomic displacement ellipsoids are drawn at the 50% probability level.

Table S2 Crystal data and structure refinement for 241135lt2_auto.

Identification code	241135lt2_auto
Empirical formula	$C_{44}H_{43}N_6O_7$
Formula weight	767.84
Temperature/K	99.98(16)
Crystal system	monoclinic
Space group	Cc
a/Å	5.14270(10)
b/Å	24.8604(5)
c/Å	30.1032(6)
α/°	90
β/°	99.967(2)
γ/°	90
Volume/Å^3	3790.59(13)
Z	4
ρ_{calc}g/cm^3	1.345
μ/mm^{-1}	0.754
F(000)	1620.0
Crystal size/mm^3	0.18 × 0.01 × 0.01
Radiation	Cu Kα (λ = 1.54184)
2Θ range for data collection/°	7.112 to 147.614
Index ranges	$-5 \leq h \leq 6$, $-29 \leq k \leq 29$, $-36 \leq l \leq 37$
Reflections collected	25555

Independent reflections	6297 [R_{int} = 0.0333, R_{sigma} = 0.0311]
Data/restraints/parameters	6297/2/522
Goodness-of-fit on F^2	1.060
Final R indexes [I>=2σ (I)]	R_1 = 0.0412, wR_2 = 0.1024
Final R indexes [all data]	R_1 = 0.0436, wR_2 = 0.1052
Largest diff. peak/hole / e Å^{-3}	0.60/-0.26
Flack parameter	0.05(10)

Table S3 Fractional Atomic Coordinates (×10^4) and Equivalent Isotropic Displacement Parameters (Å^2×10^3) for 241135lt2_auto. U_{eq} is defined as 1/3 of the trace of the orthogonalised U_{IJ} tensor.

Atom	*x*	*y*	*z*	U(eq)
O29	3688(7)	4014.2(12)	5990.4(10)	27.6(7)
O43	-127(7)	3097.4(13)	6489.4(10)	31.7(7)
O56	5960(8)	4562.5(12)	5638.6(10)	34.0(8)
N36	9568(8)	4638.9(14)	7931.6(11)	24.7(8)
N37	7710(8)	4335.6(14)	7671.8(12)	24.4(8)
N44	-193(8)	3397.4(15)	5778.3(12)	28.3(9)
C30	5671(10)	4379.3(17)	5999.0(15)	27.2(10)
C31	7153(9)	4517.5(17)	6440.4(15)	24.2(9)
C32	9195(10)	4900.2(17)	6447.1(16)	26.9(10)
C33	10652(10)	5075.8(18)	6843.5(16)	27.8(10)
C34	10017(10)	4868.5(16)	7246.2(16)	27.1(10)
C35	10981(10)	4957.7(17)	7701.9(15)	28.0(10)
C38	7974(9)	4474.3(16)	7245.2(15)	23.1(9)
C39	6454(9)	4281.7(16)	6826.1(15)	21.0(8)
C40	4373(9)	3875.9(15)	6784.4(14)	22.7(9)
C41	3063(9)	3761.2(17)	6365.5(14)	23.0(9)
C42	790(10)	3390.2(18)	6220.1(15)	25.7(9)
C45	-2375(11)	3054.7(18)	5572.3(15)	30.7(11)
C46	-1402(15)	2507(3)	5434(3)	64.2(19)
C47	351(16)	2527(4)	5105(3)	84(3)

Table S3 Fractional Atomic Coordinates (×10⁴) and Equivalent Isotropic Displacement Parameters (Å²×10³) for 241135lt2_auto. U_{eq} is defined as 1/3 of the trace of the orthogonalised U_{IJ} tensor.

Atom	*x*	*y*	*z*	U(eq)
C48	3713(11)	3608.7(17)	7199.8(14)	26.5(9)
C49	9934(10)	4590.7(17)	8425.3(14)	27.5(10)
C50	11489(9)	4092.3(17)	8599.1(14)	23.5(9)
C51	13524(10)	3897.1(18)	8394.7(15)	26.7(9)
C52	15001(10)	3451.9(18)	8572.7(15)	29.1(10)
C53	14471(11)	3205.4(19)	8960.4(17)	33.5(11)
C54	12438(11)	3393.3(18)	9166.8(15)	32.6(11)
C55	10950(10)	3838.4(19)	8985.3(15)	30.6(10)
O1	392(7)	3729.4(12)	4019.6(10)	27.3(7)
O22	-5094(7)	4489.1(12)	3521.2(10)	30.4(7)
O28	3793(8)	3269.8(14)	4361.1(11)	36.7(8)
N8	-297(8)	3130.2(14)	2349.0(12)	23.0(8)
N9	852(8)	2785.5(14)	2092.0(12)	25.9(8)
N23	-2431(8)	4500.6(15)	4205.4(12)	28.1(8)
C2	2373(10)	3371.0(18)	4003.2(15)	28.7(10)
C3	2487(10)	3130.0(17)	3565.2(15)	25.4(9)
C4	4497(9)	2737.3(17)	3558.3(15)	26.1(10)
C5	4799(11)	2487.2(17)	3170.7(15)	28.1(10)
C6	3037(10)	2640.5(16)	2769.3(15)	25.5(9)
C7	1079(9)	3044.0(16)	2773.4(14)	22.6(9)
C10	2845(10)	2493.1(17)	2318.8(16)	28.3(10)
C11	146(10)	2820.2(18)	1602.1(14)	28.4(10)
C12	1532(9)	3286.8(17)	1417.7(15)	25.9(9)
C13	3885(10)	3492.9(17)	1652.6(15)	27.2(10)
C14	5169(10)	3913.9(18)	1476.6(16)	31.2(10)
C15	4137(11)	4130.0(19)	1058.9(16)	33.8(11)
C16	1783(11)	3930(2)	826.5(17)	39.0(12)
C17	494(11)	3507(2)	1001.3(17)	35.4(11)
C18	754(9)	3303.2(16)	3183.7(14)	23.3(9)
C19	-1195(9)	3719.2(16)	3222.9(15)	24.2(9)
C20	-1314(9)	3908.2(17)	3638.7(14)	23.2(9)

Table S3 Fractional Atomic Coordinates (×10^4) and Equivalent Isotropic Displacement Parameters (Å^2×10^3) for 241135lt2_auto. U_{eq} is defined as 1/3 of the trace of the orthogonalised U_{IJ} tensor.

Atom	*x*	*y*	*z*	U(eq)
C21	-3123(10)	4321.6(17)	3779.1(14)	24.5(9)
C24	-4045(11)	4913.3(18)	4378.5(15)	30.0(10)
C25	-2458(10)	5189.7(18)	4788.3(15)	30.2(10)
C26	-4155(12)	5615(2)	4965.5(17)	39.3(12)
C27	-2972(10)	3930.8(19)	2809.2(14)	29.2(10)
O57	1804(8)	4015.2(14)	4986.6(11)	36.7(8)

Table S4 Anisotropic Displacement Parameters (Å^2×10^3) for 241135lt2_auto. The Anisotropic displacement factor exponent takes the form: -2π^2[$h^2a^{*2}U_{11}$+2hka*b*U_{12}+…].

Atom	U_{11}	U_{22}	U_{33}	U_{23}	U_{13}	U_{12}
O29	33.8(19)	29.6(15)	18.8(14)	-0.1(12)	2.5(14)	-5.4(14)
O43	33.8(18)	34.9(17)	25.6(16)	1.0(14)	2.6(15)	-10.9(15)
O56	50(2)	31.7(17)	20.8(15)	4.0(13)	6.5(15)	-8.8(16)
N36	28(2)	26.4(18)	18.7(17)	-3.5(14)	0.0(16)	-2.8(16)
N37	26(2)	24.2(18)	21.3(17)	-4.4(14)	0.2(16)	-0.6(16)
N44	32(2)	31.0(19)	20.1(18)	-1.3(15)	-1.6(16)	-3.8(17)
C30	35(3)	25(2)	22(2)	4.6(18)	4(2)	-2.3(19)
C31	27(2)	22(2)	24(2)	0.2(16)	4.9(19)	3.9(17)
C32	28(2)	22(2)	30(2)	5.1(17)	3(2)	-3.1(18)
C33	28(2)	25(2)	30(2)	0.8(18)	4(2)	-2.8(18)
C34	30(3)	22(2)	29(2)	-0.9(18)	3(2)	0.4(18)
C35	32(3)	20.2(19)	30(2)	-1.6(17)	0(2)	-4.2(18)
C38	23(2)	21.6(19)	25(2)	-1.1(16)	3.3(19)	1.5(17)
C39	22(2)	20.2(19)	21(2)	2.8(16)	1.3(18)	1.7(16)
C40	26(2)	19.6(19)	22(2)	-0.7(16)	1.2(18)	0.2(17)
C41	25(2)	21.0(19)	23(2)	1.0(15)	2.3(19)	1.7(18)
C42	28(2)	25(2)	23(2)	-0.3(16)	1.3(19)	3.1(18)
C45	37(3)	32(2)	21(2)	-2.1(17)	-3(2)	-4(2)
C46	51(4)	67(4)	73(4)	-23(3)	7(4)	-14(3)

Table S4 Anisotropic Displacement Parameters (Å^2×10^3) for 241135lt2_auto. The Anisotropic displacement factor exponent takes the form: -2π^2[h^{2}a*^{2}U$_{11}$+2hka*b*U$_{12}$+…].

Atom	U_{11}	U_{22}	U_{33}	U_{23}	U_{13}	U_{12}
C47	48(4)	98(6)	109(7)	-66(5)	22(4)	-18(4)
C48	34(2)	26(2)	19.4(19)	-0.8(16)	3.2(19)	-8.5(19)
C49	30(2)	31(2)	20(2)	-2.7(16)	-1(2)	0(2)
C50	25(2)	27(2)	17.7(18)	-5.9(16)	1.3(17)	-2.9(18)
C51	25(2)	30(2)	23(2)	-4.0(17)	0.7(18)	-3.7(18)
C52	26(2)	30(2)	31(2)	-6.1(18)	2(2)	-2(2)
C53	36(3)	28(2)	33(2)	-1.7(19)	-6(2)	-2(2)
C54	39(3)	30(2)	26(2)	0.2(18)	-3(2)	-7(2)
C55	34(3)	35(2)	21(2)	-6.2(18)	0(2)	-8(2)
O1	31.9(17)	30.4(16)	18.1(13)	1.6(12)	-0.1(14)	7.0(14)
O22	29.3(18)	33.9(16)	24.7(15)	-2.3(12)	-4.4(15)	7.3(14)
O28	42(2)	39.2(18)	25.3(16)	0.3(14)	-4.6(15)	10.5(16)
N8	26(2)	22.5(17)	21.0(17)	-0.3(14)	4.8(15)	-0.9(15)
N9	30(2)	22.3(18)	25.9(18)	-3.0(15)	5.1(17)	-3.1(16)
N23	31(2)	28.1(18)	23.9(18)	-1.1(15)	2.8(17)	4.0(17)
C2	33(3)	29(2)	22(2)	4.1(17)	1(2)	5(2)
C3	27(2)	23(2)	25(2)	4.9(16)	0.9(18)	1.6(18)
C4	28(2)	26(2)	22(2)	5.7(16)	-1(2)	3.1(18)
C5	31(2)	25(2)	28(2)	4.6(17)	4(2)	1.2(19)
C6	29(2)	19(2)	28(2)	3.9(16)	5(2)	1.8(18)
C7	27(2)	20.7(18)	21(2)	5.9(16)	7.4(18)	-0.8(17)
C10	29(2)	21(2)	34(2)	2.7(17)	6(2)	-0.1(19)
C11	34(3)	29(2)	21(2)	-3.5(17)	1.0(19)	-3.4(19)
C12	28(2)	28(2)	22(2)	-0.1(17)	5.0(19)	2.2(18)
C13	35(3)	26(2)	20.4(19)	-0.4(17)	4.0(18)	3.7(19)
C14	32(3)	30(2)	32(2)	-3.9(19)	4(2)	-2.7(19)
C15	39(3)	32(2)	32(3)	4(2)	12(2)	3(2)
C16	38(3)	48(3)	29(3)	9(2)	2(2)	8(2)
C17	30(3)	49(3)	25(2)	7(2)	1(2)	0(2)
C18	26(2)	20(2)	23(2)	3.0(15)	0.8(18)	-2.5(17)
C19	25(2)	22(2)	25(2)	1.2(17)	1.3(19)	-1.8(18)

Table S4 Anisotropic Displacement Parameters (Å²×10³) for 241135lt2_auto. The Anisotropic displacement factor exponent takes the form: -2π²[h²a*²U11+2hka*b*U12+…].

Atom	U_{11}	U_{22}	U_{33}	U_{23}	U_{13}	U_{12}
C20	22(2)	27(2)	18(2)	2.6(16)	-3.6(18)	-1.5(17)
C21	27(2)	22.6(19)	23(2)	4.4(16)	2.0(19)	4.1(18)
C24	34(3)	32(2)	24(2)	-4.1(18)	4(2)	5(2)
C25	31(3)	31(2)	28(2)	-1.8(18)	5(2)	2(2)
C26	47(3)	40(3)	28(2)	-6(2)	-2(2)	7(2)
C27	31(3)	31(2)	23(2)	-1.4(18)	-3(2)	2.2(19)
O57	42(2)	42.8(19)	22.8(16)	-1.8(13)	-0.7(16)	6.5(17)

Table S5 Bond Lengths for 241135lt2_auto.

Atom	Atom	Length/Å	Atom	Atom	Length/Å
O29	C30	1.362(5)	O1	C2	1.361(6)
O29	C41	1.378(5)	O1	C20	1.391(5)
O43	C42	1.242(6)	O22	C21	1.237(5)
O56	C30	1.209(6)	O28	C2	1.219(5)
N36	N37	1.354(5)	N8	N9	1.356(5)
N36	C35	1.345(6)	N8	C7	1.366(5)
N36	C49	1.470(5)	N9	C10	1.343(6)
N37	C38	1.359(6)	N9	C11	1.459(5)
N44	C42	1.339(5)	N23	C21	1.347(5)
N44	C45	1.459(6)	N23	C24	1.471(6)
C30	C31	1.454(6)	C2	C3	1.459(6)
C31	C32	1.415(6)	C3	C4	1.425(6)
C31	C39	1.402(6)	C3	C18	1.394(6)
C32	C33	1.366(7)	C4	C5	1.354(7)
C33	C34	1.407(7)	C5	C6	1.431(6)
C34	C35	1.393(6)	C6	C7	1.423(6)
C34	C38	1.437(6)	C6	C10	1.392(7)
C38	C39	1.446(6)	C7	C18	1.428(6)
C39	C40	1.460(6)	C11	C12	1.516(6)
C40	C41	1.353(6)	C12	C13	1.389(7)

Table S5 Bond Lengths for 241135lt2_auto.

Atom	Atom	Length/Å	Atom	Atom	Length/Å
C40	C48	1.506(6)	C12	C17	1.387(6)
C41	C42	1.494(6)	C13	C14	1.390(7)
C45	C46	1.533(9)	C14	C15	1.386(6)
C46	C47	1.450(11)	C15	C16	1.382(8)
C49	C50	1.518(6)	C16	C17	1.393(7)
C50	C51	1.390(7)	C18	C19	1.459(6)
C50	C55	1.392(7)	C19	C20	1.348(6)
C51	C52	1.396(7)	C19	C27	1.506(6)
C52	C53	1.386(7)	C20	C21	1.496(6)
C53	C54	1.386(8)	C24	C25	1.520(6)
C54	C55	1.402(7)	C25	C26	1.525(7)

Table S6 Bond Angles for 241135lt2_auto.

Atom	Atom	Atom	Angle/°	Atom	Atom	Atom	Angle/°
C30	O29	C41	124.6(4)	C2	O1	C20	123.3(4)
N37	N36	C49	119.5(4)	N9	N8	C7	103.2(3)
C35	N36	N37	114.9(3)	N8	N9	C11	118.8(4)
C35	N36	C49	125.6(4)	C10	N9	N8	114.9(4)
N36	N37	C38	103.2(4)	C10	N9	C11	125.5(4)
C42	N44	C45	123.0(4)	C21	N23	C24	119.7(4)
O29	C30	C31	116.6(4)	O1	C2	C3	116.7(4)
O56	C30	O29	116.2(4)	O28	C2	O1	116.2(4)
O56	C30	C31	127.2(4)	O28	C2	C3	127.1(4)
C32	C31	C30	116.3(4)	C4	C3	C2	115.9(4)
C39	C31	C30	119.3(4)	C18	C3	C2	119.9(4)
C39	C31	C32	124.4(4)	C18	C3	C4	124.2(4)
C33	C32	C31	121.4(4)	C5	C4	C3	121.2(4)
C32	C33	C34	117.5(4)	C4	C5	C6	117.1(4)
C33	C34	C38	121.8(4)	C7	C6	C5	121.7(4)
C35	C34	C33	134.0(5)	C10	C6	C5	132.9(4)
C35	C34	C38	104.2(4)	C10	C6	C7	105.3(4)
N36	C35	C34	106.3(4)	N8	C7	C6	110.8(4)

Table S6 Bond Angles for 241135lt2_auto.

Atom	Atom	Atom	Angle/°	Atom	Atom	Atom	Angle/°
N37	C38	C34	111.3(4)	N8	C7	C18	128.2(4)
N37	C38	C39	127.8(4)	C6	C7	C18	120.9(4)
C34	C38	C39	120.8(4)	N9	C10	C6	105.8(4)
C31	C39	C38	114.0(4)	N9	C11	C12	111.4(4)
C31	C39	C40	120.4(4)	C13	C12	C11	121.3(4)
C38	C39	C40	125.5(4)	C17	C12	C11	120.1(4)
C39	C40	C48	120.0(4)	C17	C12	C13	118.6(4)
C41	C40	C39	117.7(4)	C12	C13	C14	120.9(4)
C41	C40	C48	122.2(4)	C15	C14	C13	120.3(5)
O29	C41	C42	108.9(3)	C16	C15	C14	119.0(5)
C40	C41	O29	121.2(4)	C15	C16	C17	120.8(5)
C40	C41	C42	129.9(4)	C12	C17	C16	120.4(5)
O43	C42	N44	122.4(4)	C3	C18	C7	114.8(4)
O43	C42	C41	122.5(4)	C3	C18	C19	120.2(4)
N44	C42	C41	115.1(4)	C7	C18	C19	125.0(4)
N44	C45	C46	111.8(5)	C18	C19	C27	120.5(4)
C47	C46	C45	115.3(7)	C20	C19	C18	117.7(4)
N36	C49	C50	112.5(4)	C20	C19	C27	121.8(4)
C51	C50	C49	121.6(4)	O1	C20	C21	108.7(3)
C51	C50	C55	119.0(4)	C19	C20	O1	122.0(4)
C55	C50	C49	119.3(4)	C19	C20	C21	129.2(4)
C50	C51	C52	120.6(4)	O22	C21	N23	122.6(4)
C53	C52	C51	120.0(5)	O22	C21	C20	122.4(4)
C52	C53	C54	120.1(5)	N23	C21	C20	114.9(4)
C53	C54	C55	119.6(5)	N23	C24	C25	110.3(4)
C50	C55	C54	120.6(5)	C24	C25	C26	109.7(4)

Table S7 Torsion Angles for 241135lt2_auto.

A	B	C	D	Angle/°	A	B	C	D	Angle/°
O29	C30	C31	C32	-179.5(4)	O1	C2	C3	C4	-177.2(4)
O29	C30	C31	C39	-1.1(6)	O1	C2	C3	C18	5.2(6)
O29	C41	C42	O43	177.6(4)	O1	C20	C21	O22	-168.8(4)

Table S7 Torsion Angles for 241135lt2_auto.

A	B	C	D	Angle/°	A	B	C	D	Angle/°
O29	C41	C42	N44	-2.6(6)	O1	C20	C21	N23	11.8(5)
O56	C30	C31	C32	-1.5(7)	O28	C2	C3	C4	-0.5(7)
O56	C30	C31	C39	176.9(5)	O28	C2	C3	C18	-178.1(5)
N36	N37	C38	C34	-0.2(5)	N8	N9	C10	C6	1.5(5)
N36	N37	C38	C39	-178.8(4)	N8	N9	C11	C12	77.8(5)
N36	C49	C50	C51	34.9(6)	N8	C7	C18	C3	-179.4(4)
N36	C49	C50	C55	-148.2(4)	N8	C7	C18	C19	0.5(7)
N37	N36	C35	C34	0.1(5)	N9	N8	C7	C6	-0.7(5)
N37	N36	C49	C50	78.0(5)	N9	N8	C7	C18	179.0(4)
N37	C38	C39	C31	177.9(4)	N9	C11	C12	C13	24.5(6)
N37	C38	C39	C40	-2.7(7)	N9	C11	C12	C17	-157.3(5)
N44	C45	C46	C47	-60.7(7)	N23	C24	C25	C26	-179.8(4)
C30	O29	C41	C40	2.2(7)	C2	O1	C20	C19	2.9(7)
C30	O29	C41	C42	-179.9(4)	C2	O1	C20	C21	-176.6(4)
C30	C31	C32	C33	177.5(4)	C2	C3	C4	C5	-179.8(4)
C30	C31	C39	C38	-176.6(4)	C2	C3	C18	C7	179.1(4)
C30	C31	C39	C40	3.9(6)	C2	C3	C18	C19	-0.8(6)
C31	C32	C33	C34	-1.1(7)	C3	C4	C5	C6	0.9(7)
C31	C39	C40	C41	-3.7(6)	C3	C18	C19	C20	-2.7(6)
C31	C39	C40	C48	177.5(4)	C3	C18	C19	C27	176.6(4)
C32	C31	C39	C38	1.6(6)	C4	C3	C18	C7	1.7(6)
C32	C31	C39	C40	-177.9(4)	C4	C3	C18	C19	-178.2(4)
C32	C33	C34	C35	-178.2(5)	C4	C5	C6	C7	1.0(7)
C32	C33	C34	C38	1.9(7)	C4	C5	C6	C10	176.5(5)
C33	C34	C35	N36	179.9(5)	C5	C6	C7	N8	178.1(4)
C33	C34	C38	N37	-179.9(4)	C5	C6	C7	C18	-1.6(7)
C33	C34	C38	C39	-1.1(7)	C5	C6	C10	N9	-177.8(5)
C34	C38	C39	C31	-0.7(6)	C6	C7	C18	C3	0.2(6)
C34	C38	C39	C40	178.8(4)	C6	C7	C18	C19	-179.8(4)
C35	N36	N37	C38	0.0(5)	C7	N8	N9	C10	-0.5(5)
C35	N36	C49	C50	-100.5(5)	C7	N8	N9	C11	-171.1(4)
C35	C34	C38	N37	0.2(5)	C7	C6	C10	N9	-1.7(5)
C35	C34	C38	C39	179.0(4)	C7	C18	C19	C20	177.4(4)

Table S7 Torsion Angles for 241135lt2_auto.

A	B	C	D	Angle/°	A	B	C	D	Angle/°
C38	C34	C35	N36	-0.2(5)	C7	C18	C19	C27	-3.4(7)
C38	C39	C40	C41	176.9(4)	C10	N9	C11	C12	-91.7(5)
C38	C39	C40	C48	-1.9(6)	C10	C6	C7	N8	1.6(5)
C39	C31	C32	C33	-0.8(7)	C10	C6	C7	C18	-178.2(4)
C39	C40	C41	O29	0.7(6)	C11	N9	C10	C6	171.4(4)
C39	C40	C41	C42	-176.8(4)	C11	C12	C13	C14	178.7(4)
C40	C41	C42	O43	-4.7(8)	C11	C12	C17	C16	-178.9(5)
C40	C41	C42	N44	175.1(5)	C12	C13	C14	C15	-1.0(7)
C41	O29	C30	O56	179.8(4)	C13	C12	C17	C16	-0.6(7)
C41	O29	C30	C31	-2.0(6)	C13	C14	C15	C16	1.6(7)
C42	N44	C45	C46	-87.9(6)	C14	C15	C16	C17	-1.8(8)
C45	N44	C42	O43	-1.5(7)	C15	C16	C17	C12	1.3(8)
C45	N44	C42	C41	178.7(4)	C17	C12	C13	C14	0.5(7)
C48	C40	C41	O29	179.5(4)	C18	C3	C4	C5	-2.3(7)
C48	C40	C41	C42	2.0(7)	C18	C19	C20	O1	1.8(6)
C49	N36	N37	C38	-178.7(4)	C18	C19	C20	C21	-178.8(4)
C49	N36	C35	C34	178.7(4)	C19	C20	C21	O22	11.8(8)
C49	C50	C51	C52	177.2(4)	C19	C20	C21	N23	-167.7(5)
C49	C50	C55	C54	-176.8(4)	C20	O1	C2	O28	176.6(4)
C50	C51	C52	C53	-1.0(7)	C20	O1	C2	C3	-6.3(6)
C51	C50	C55	C54	0.2(7)	C21	N23	C24	C25	-160.3(4)
C51	C52	C53	C54	1.4(7)	C24	N23	C21	O22	-0.3(7)
C52	C53	C54	C55	-1.0(7)	C24	N23	C21	C20	179.2(4)
C53	C54	C55	C50	0.2(7)	C27	C19	C20	O1	-177.4(4)
C55	C50	C51	C52	0.2(7)	C27	C19	C20	C21	2.0(8)

Table S8 Hydrogen Atom Coordinates (Å×10^4) and Isotropic Displacement Parameters (Å^2×10^3) for 241135lt2_auto.

Atom	*x*	*y*	*z*	U(eq)
H44	509.62	3618.98	5604.14	34
H32	9561.41	5038.03	6170.09	32
H33	12042.44	5328.28	6847	33

Table S8 Hydrogen Atom Coordinates (Å×10^4) and Isotropic Displacement Parameters (Å^2×10^3) for 241135lt2_auto.

Atom	*x*	*y*	*z*	U(eq)
H35	12361.16	5196.21	7825.6	34
H45A	-3367.35	3237.11	5302.94	37
H45B	-3595.56	2999.12	5788.97	37
H46A	-473.45	2323.23	5707.26	77
H46B	-2956.97	2285.79	5308.64	77
H47A	-572.31	2691.13	4825.45	126
H47B	899.22	2161.39	5041.95	126
H47C	1911.83	2742.43	5224.66	126
H48A	5330.03	3465.82	7381.94	40
H48B	2467.06	3313.91	7110.66	40
H48C	2913.32	3872.56	7377.09	40
H49A	8182.22	4578.24	8519.04	33
H49B	10871.65	4913.38	8563.69	33
H51	13912.71	4068.07	8131.68	32
H52	16369.7	3317.73	8428.11	35
H53	15502.79	2907.29	9084.88	40
H54	12055.8	3221.18	9429.8	39
H55	9560.09	3968.13	9127.11	37
H4	5647.78	2649.26	3830.65	31
H5	6121.91	2221.74	3165.63	34
H10	3905.26	2238.23	2196.88	34
H11A	-1788.46	2866.57	1517.09	34
H11B	636.11	2480.64	1465.96	34
H13	4625.95	3344.14	1937.11	33
H14	6761.92	4054.07	1643.59	37
H15	5033.36	4411.43	934.33	41
H16	1034.13	4082.35	543.86	47
H17	-1104.53	3369.5	834.55	42
H24A	-5634.07	4744.18	4462.44	36
H24B	-4631.38	5182.98	4140.16	36
H25A	-873.3	5360.77	4704.6	36
H25B	-1867.7	4920.5	5026.87	36

Table S8 Hydrogen Atom Coordinates (Å×10⁴) and Isotropic Displacement Parameters (Å²×10³) for 241135lt2_auto.

Atom	*x*	*y*	*z*	U(eq)
H26A	-4513.95	5908.61	4746.2	59
H26B	-3216.69	5756.59	5252.76	59
H26C	-5826.35	5453.11	5011.28	59
H27A	-1989.86	3948.37	2558.08	44
H27B	-3591.11	4291.49	2870.91	44
H27C	-4490.61	3690.3	2729.38	44
H57A	3047.35	4170.57	5175.92	55
H57B	2640.03	3814.81	4820.46	55

X-ray single crystallographic data of the compounds 3w

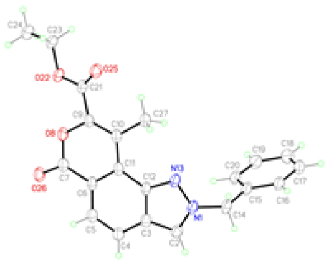

CCDC 2430338

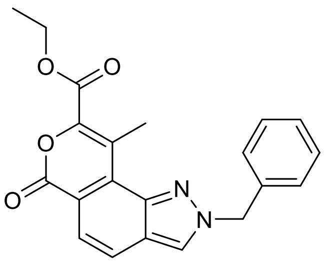

3w

ORTEP diagram of compound 3w. Atomic displacement ellipsoids are drawn at the 50% probability level.

Table S9 Crystal data and structure refinement for 240874lt_auto.

Identification code	240874lt_auto
Empirical formula	$C_{21}H_{18}N_2O_4$
Formula weight	362.37
Temperature/K	100.00(12)
Crystal system	monoclinic
Space group	$P2_1/c$
a/Å	13.2579(6)
b/Å	18.5134(8)

c/Å	6.9129(3)
α/°	90
β/°	99.399(5)
γ/°	90
Volume/$Å^3$	1673.98(13)
Z	4
$\rho_{calc}g/cm^3$	1.438
μ/mm^{-1}	0.827
F(000)	760.0
Crystal size/mm^3	0.1 × 0.02 × 0.01
Radiation	Cu Kα (λ = 1.54184)
2Θ range for data collection/°	6.758 to 146.304
Index ranges	-16 ≤ h ≤ 16, -20 ≤ k ≤ 22, -6 ≤ l ≤ 8
Reflections collected	10881
Independent reflections	3223 [R_{int} = 0.0492, R_{sigma} = 0.0503]
Data/restraints/parameters	3223/0/247
Goodness-of-fit on F^2	1.047
Final R indexes [I>=2σ (I)]	R_1 = 0.0676, wR_2 = 0.1861
Final R indexes [all data]	R_1 = 0.0872, wR_2 = 0.2006
Largest diff. peak/hole / e $Å^{-3}$	0.41/-0.42

Table S10 Fractional Atomic Coordinates (×10^4) and Equivalent Isotropic Displacement Parameters ($Å^2$×10^3) for 240874lt_auto. U_{eq} is defined as 1/3 of the trace of the orthogonalised U_{IJ} tensor.

**Atom**	***x***	***y***	***z***	**U(eq)**
O(8)	7562.3(14)	1771.4(9)	3620(3)	27.7(5)
O(22)	5725.3(14)	1405.8(10)	3371(3)	30.2(5)
O(25)	5249.3(15)	2433.5(10)	4695(3)	31.5(5)
O(26)	9041.0(15)	1321.8(10)	3167(3)	31.5(5)
N(1)	8907.9(17)	5017.1(12)	2836(4)	28.2(5)
N(13)	8179.5(18)	4541.4(12)	3164(3)	27.5(5)
C(2)	9806(2)	4719.1(15)	2642(4)	28.5(6)
C(3)	9687(2)	3976.9(14)	2848(4)	27.0(6)
C(4)	10341(2)	3374.9(15)	2777(4)	29.2(6)
C(5)	9943(2)	2707.7(15)	2974(4)	28.6(6)

Table S10 Fractional Atomic Coordinates (×10[4]) and Equivalent Isotropic Displacement Parameters (Å[2]×10[3]) for 240874lt_auto. U_{eq} is defined as 1/3 of the trace of the orthogonalised U_{IJ} tensor.

Atom	*x*	*y*	*z*	U(eq)
C(6)	8914(2)	2607.1(14)	3249(4)	26.1(6)
C(7)	8546(2)	1868.7(14)	3324(4)	27.9(6)
C(9)	6915(2)	2347.4(14)	3778(4)	25.7(6)
C(10)	7201(2)	3044.8(14)	3661(4)	26.9(6)
C(11)	8242(2)	3187.1(14)	3377(4)	25.6(6)
C(12)	8662(2)	3888.0(13)	3168(4)	26.0(6)
C(14)	8629(2)	5774.6(14)	2591(4)	30.7(6)
C(15)	7976(2)	5939.6(14)	634(4)	29.2(6)
C(16)	7571(2)	6633.3(15)	323(5)	31.9(7)
C(17)	6968(2)	6813.9(16)	-1444(5)	36.4(7)
C(18)	6768(2)	6311.0(17)	-2935(5)	37.7(7)
C(19)	7178(2)	5619.4(16)	-2646(5)	35.8(7)
C(20)	7771(2)	5430.1(15)	-870(4)	30.1(6)
C(21)	5882(2)	2083.7(14)	4015(4)	27.3(6)
C(23)	4715(2)	1121.5(14)	3474(5)	31.1(7)
C(24)	4633(2)	396.2(16)	2492(5)	37.2(7)
C(27)	6466(2)	3663.5(15)	3704(5)	32.6(7)

Table S11 Anisotropic Displacement Parameters (Å[2]×10[3]) for 240874lt_auto. The Anisotropic displacement factor exponent takes the form: -2π[2][h[2]a*[2]U_{11}+2hka*b*U_{12}+…].

Atom	U_{11}	U_{22}	U_{33}	U_{23}	U_{13}	U_{12}
O(8)	32.5(10)	16.7(9)	33.7(11)	0.2(8)	4.5(8)	2.2(7)
O(22)	34.9(11)	16.1(9)	39.8(12)	-1.6(8)	6.3(8)	-2.9(7)
O(25)	35.3(11)	21.9(10)	38.4(12)	-2.7(8)	8.9(9)	-0.6(8)
O(26)	37.8(11)	17.9(10)	38.2(12)	-0.6(8)	4.3(9)	3.9(8)
N(1)	34.6(12)	17.0(11)	32.6(13)	0.5(9)	4.3(10)	-3.6(9)
N(13)	34.4(13)	16.0(11)	31.9(13)	0.4(9)	4.4(10)	-2.5(9)
C(2)	31.5(15)	21.5(13)	32.0(15)	-0.8(11)	3.1(11)	-2.1(10)
C(3)	33.6(15)	21.0(13)	25.2(14)	-1.4(11)	1.1(11)	0.1(11)

Table S11 Anisotropic Displacement Parameters (Å^2×10^3) for 240874lt_auto. The Anisotropic displacement factor exponent takes the form: -2π^2[h^{2}a*^{2}U$_{11}$+2hka*b*U$_{12}$+…].

Atom	U_{11}	U_{22}	U_{33}	U_{23}	U_{13}	U_{12}
C(4)	31.3(14)	23.2(14)	32.2(15)	0.9(11)	2.5(11)	1.5(11)
C(5)	34.7(15)	21.6(13)	28.9(14)	-0.9(11)	3.1(12)	2.6(11)
C(6)	34.6(15)	16.5(13)	26.0(14)	0.0(10)	0.7(11)	0.7(10)
C(7)	34.4(15)	20.3(14)	27.9(15)	0.7(10)	1.8(11)	1.9(11)
C(9)	31.4(14)	17.4(13)	27.2(14)	-1.0(10)	1.7(11)	3.4(10)
C(10)	33.0(15)	20.1(13)	26.4(14)	1.5(10)	1.5(11)	2.0(10)
C(11)	33.5(14)	17.5(13)	24.7(13)	-0.3(10)	1.3(11)	2.6(10)
C(12)	33.7(15)	14.7(12)	28.8(14)	0.3(10)	2.4(11)	0.1(10)
C(14)	41.3(16)	15.5(13)	35.0(15)	-0.8(11)	5.7(12)	-3.0(11)
C(15)	35.0(15)	17.4(13)	35.6(16)	3.0(11)	7.4(12)	-2.1(11)
C(16)	34.4(15)	20.3(14)	43.1(17)	2.3(12)	12.7(13)	0.2(11)
C(17)	36.8(16)	26.2(15)	47.3(18)	12.0(13)	10.4(13)	3.6(12)
C(18)	38.1(16)	36.7(17)	37.0(17)	10.7(13)	2.0(13)	-0.8(13)
C(19)	39.3(17)	31.0(16)	36.8(17)	1.6(13)	5.4(13)	-4.9(12)
C(20)	35.4(15)	20.7(13)	34.2(16)	4.3(12)	5.7(12)	-1.7(11)
C(21)	34.4(15)	17.8(13)	29.4(14)	1.7(11)	4.4(11)	-0.6(11)
C(23)	32.5(15)	19.7(14)	41.3(17)	-1.1(12)	6.1(12)	-4.1(11)
C(24)	41.7(17)	21.3(14)	48.1(19)	-3.9(13)	5.8(14)	-3.2(12)
C(27)	33.2(15)	20.5(14)	44.3(17)	1.8(12)	7.5(13)	3.4(11)

Table S12 Bond Lengths for 240874lt_auto.

Atom	Atom	Length/Å	Atom	Atom	Length/Å
O(8)	C(7)	1.365(3)	C(6)	C(7)	1.455(4)
O(8)	C(9)	1.384(3)	C(6)	C(11)	1.408(4)
O(22)	C(21)	1.336(3)	C(9)	C(10)	1.352(4)
O(22)	C(23)	1.451(3)	C(9)	C(21)	1.489(4)
O(25)	C(21)	1.213(3)	C(10)	C(11)	1.450(4)
O(26)	C(7)	1.221(3)	C(10)	C(27)	1.507(4)
N(1)	N(13)	1.353(3)	C(11)	C(12)	1.429(3)
N(1)	C(2)	1.339(4)	C(14)	C(15)	1.514(4)

Table S12 Bond Lengths for 240874lt_auto.

Atom	Atom	Length/Å	Atom	Atom	Length/Å
N(1)	C(14)	1.453(3)	C(15)	C(16)	1.395(4)
N(13)	C(12)	1.368(3)	C(15)	C(20)	1.397(4)
C(2)	C(3)	1.393(4)	C(16)	C(17)	1.387(4)
C(3)	C(4)	1.418(4)	C(17)	C(18)	1.382(5)
C(3)	C(12)	1.421(4)	C(18)	C(19)	1.392(4)
C(4)	C(5)	1.359(4)	C(19)	C(20)	1.390(4)
C(5)	C(6)	1.419(4)	C(23)	C(24)	1.501(4)

Table S13 Bond Angles for 240874lt_auto.

Atom	Atom	Atom	Angle/°	Atom	Atom	Atom	Angle/°
C(7)	O(8)	C(9)	122.0(2)	C(9)	C(10)	C(27)	122.4(3)
C(21)	O(22)	C(23)	114.7(2)	C(11)	C(10)	C(27)	119.8(2)
N(13)	N(1)	C(14)	118.2(2)	C(6)	C(11)	C(10)	119.8(2)
C(2)	N(1)	N(13)	114.8(2)	C(6)	C(11)	C(12)	115.1(2)
C(2)	N(1)	C(14)	126.8(2)	C(12)	C(11)	C(10)	125.1(2)
N(1)	N(13)	C(12)	103.2(2)	N(13)	C(12)	C(3)	110.9(2)
N(1)	C(2)	C(3)	106.2(2)	N(13)	C(12)	C(11)	127.8(3)
C(2)	C(3)	C(4)	133.7(3)	C(3)	C(12)	C(11)	121.3(2)
C(2)	C(3)	C(12)	104.9(2)	N(1)	C(14)	C(15)	113.3(2)
C(4)	C(3)	C(12)	121.4(2)	C(16)	C(15)	C(14)	118.2(3)
C(5)	C(4)	C(3)	117.4(3)	C(16)	C(15)	C(20)	118.9(3)
C(4)	C(5)	C(6)	122.0(3)	C(20)	C(15)	C(14)	122.9(2)
C(5)	C(6)	C(7)	117.6(2)	C(17)	C(16)	C(15)	120.6(3)
C(11)	C(6)	C(5)	122.7(2)	C(18)	C(17)	C(16)	120.5(3)
C(11)	C(6)	C(7)	119.6(3)	C(17)	C(18)	C(19)	119.3(3)
O(8)	C(7)	C(6)	117.6(2)	C(20)	C(19)	C(18)	120.6(3)
O(26)	C(7)	O(8)	116.4(2)	C(19)	C(20)	C(15)	120.0(3)
O(26)	C(7)	C(6)	126.0(3)	O(22)	C(21)	C(9)	111.5(2)
O(8)	C(9)	C(21)	110.5(2)	O(25)	C(21)	O(22)	123.4(3)
C(10)	C(9)	O(8)	123.2(2)	O(25)	C(21)	C(9)	125.1(2)
C(10)	C(9)	C(21)	126.3(2)	O(22)	C(23)	C(24)	107.6(2)
C(9)	C(10)	C(11)	117.6(2)				

Table S14 Torsion Angles for 240874lt_auto.

A	B	C	D	Angle/°	A	B	C	D	Angle/°
O(8)	C(9)	C(10)	C(11)	-0.7(4)	C(7)	C(6)	C(11)	C(10)	2.4(4)
O(8)	C(9)	C(10)	C(27)	175.9(2)	C(7)	C(6)	C(11)	C(12)	-176.3(2)
O(8)	C(9)	C(21)	O(22)	-21.0(3)	C(9)	O(8)	C(7)	O(26)	-178.5(2)
O(8)	C(9)	C(21)	O(25)	159.3(3)	C(9)	O(8)	C(7)	C(6)	2.3(4)
N(1)	N(13)	C(12)	C(3)	0.2(3)	C(9)	C(10)	C(11)	C(6)	-0.5(4)
N(1)	N(13)	C(12)	C(11)	-177.4(3)	C(9)	C(10)	C(11)	C(12)	178.2(3)
N(1)	C(2)	C(3)	C(4)	179.3(3)	C(10)	C(9)	C(21)	O(22)	156.9(3)
N(1)	C(2)	C(3)	C(12)	0.0(3)	C(10)	C(9)	C(21)	O(25)	-22.8(5)
N(1)	C(14)	C(15)	C(16)	173.1(2)	C(10)	C(11)	C(12)	N(13)	-1.0(5)
N(1)	C(14)	C(15)	C(20)	-7.4(4)	C(10)	C(11)	C(12)	C(3)	-178.5(3)
N(13)	N(1)	C(2)	C(3)	0.1(3)	C(11)	C(6)	C(7)	O(8)	-3.3(4)
N(13)	N(1)	C(14)	C(15)	-73.8(3)	C(11)	C(6)	C(7)	O(26)	177.6(3)
C(2)	N(1)	N(13)	C(12)	-0.2(3)	C(12)	C(3)	C(4)	C(5)	1.7(4)
C(2)	N(1)	C(14)	C(15)	101.3(3)	C(14)	N(1)	N(13)	C(12)	175.4(2)
C(2)	C(3)	C(4)	C(5)	-177.5(3)	C(14)	N(1)	C(2)	C(3)	-175.1(3)
C(2)	C(3)	C(12)	N(13)	-0.2(3)	C(14)	C(15)	C(16)	C(17)	180.0(3)
C(2)	C(3)	C(12)	C(11)	177.7(3)	C(14)	C(15)	C(20)	C(19)	-179.0(3)
C(3)	C(4)	C(5)	C(6)	-0.3(4)	C(15)	C(16)	C(17)	C(18)	-0.8(5)
C(4)	C(3)	C(12)	N(13)	-179.6(2)	C(16)	C(15)	C(20)	C(19)	0.5(4)
C(4)	C(3)	C(12)	C(11)	-1.7(4)	C(16)	C(17)	C(18)	C(19)	0.1(5)
C(4)	C(5)	C(6)	C(7)	176.4(3)	C(17)	C(18)	C(19)	C(20)	0.8(5)
C(4)	C(5)	C(6)	C(11)	-1.3(4)	C(18)	C(19)	C(20)	C(15)	-1.1(4)
C(5)	C(6)	C(7)	O(8)	179.0(2)	C(20)	C(15)	C(16)	C(17)	0.5(4)
C(5)	C(6)	C(7)	O(26)	-0.2(4)	C(21)	O(22)	C(23)	C(24)	173.6(2)
C(5)	C(6)	C(11)	C(10)	-180.0(2)	C(21)	C(9)	C(10)	C(11)	-178.4(2)
C(5)	C(6)	C(11)	C(12)	1.3(4)	C(21)	C(9)	C(10)	C(27)	-1.8(4)
C(6)	C(11)	C(12)	N(13)	177.7(3)	C(23)	O(22)	C(21)	O(25)	2.3(4)
C(6)	C(11)	C(12)	C(3)	0.2(4)	C(23)	O(22)	C(21)	C(9)	-177.4(2)
C(7)	O(8)	C(9)	C(10)	-0.3(4)	C(27)	C(10)	C(11)	C(6)	-177.2(3)
C(7)	O(8)	C(9)	C(21)	177.7(2)	C(27)	C(10)	C(11)	C(12)	1.5(4)

Table S15 Hydrogen Atom Coordinates (Å×10^4) and Isotropic Displacement Parameters (Å^2×10^3) for 240874lt_auto.

Atom	*x*	*y*	*z*	U(eq)
H(2)	10403.97	4964.43	2409.43	34
H(4)	11031.85	3435.73	2598.78	35
H(5)	10365.47	2295.94	2925.26	34
H(14A)	9261.13	6068.19	2724.37	37
H(14B)	8252.39	5919.19	3653.04	37
H(16)	7710.32	6985.41	1330.83	38
H(17)	6690.15	7286.57	-1629.98	44
H(18)	6356.11	6435.94	-4144.79	45
H(19)	7050.49	5273.46	-3671.6	43
H(20)	8036.7	4954.27	-678.93	36
H(23A)	4183.49	1451.4	2800.06	37
H(23B)	4619.61	1073.1	4858.88	37
H(24A)	4737.46	449.78	1128.3	56
H(24B)	3952.24	193.48	2519.66	56
H(24C)	5153.83	71.72	3185.74	56
H(27A)	6685.73	3958.44	4874.24	49
H(27B)	5778.88	3473.21	3737.95	49
H(27C)	6454.08	3961.23	2528.57	49
